# Sex disparity in acute myeloid leukaemia with *FLT3* internal tandem duplication mutations: implications for prognosis

**DOI:** 10.1002/1878-0261.13035

**Published:** 2021-06-20

**Authors:** Monica Hellesøy, Caroline Engen, Tim Grob, Bob Löwenberg, Peter J. M. Valk, Bjørn T. Gjertsen

**Affiliations:** ^1^ Haematology Section Department of Medicine Haukeland University Hospital Helse Bergen HF Norway; ^2^ Department of Clinical Science Center for Cancer Biomarkers CCBIO University of Bergen Norway; ^3^ Department of Hematology Erasmus University Medical Center Rotterdam The Netherlands

**Keywords:** acute myeloid leukaemia, context‐dependency, *FLT3*, *FLT3‐*ITD, sex disparity

## Abstract

Incidence, molecular presentation and outcome of acute myeloid leukaemia (AML) are influenced by sex, but little attention has been directed at untangling sex‐related molecular and phenotypic differences between female and male patients. While increased incidence and poor risk are generally associated with a male phenotype, the poor prognostic *FLT3* internal tandem duplication (*FLT3*‐ITD) mutation and co‐mutations with *NPM1* and *DNMT3A* are overrepresented in female AML. Here, we have investigated the relationship between sex and *FLT3*‐ITD mutation status by comparing clinical data, mutational profiles, gene expression and *ex vivo* drug sensitivity in four cohorts: Beat AML, LAML‐TCGA and two independent HOVON/SAKK cohorts, comprising 1755 AML patients in total. We found prevalent sex‐associated molecular differences. Co‐occurrence of *FLT3*‐ITD, *NPM1* and *DNMT3A* mutations was overrepresented in females, while males with *FLT3*‐ITDs were characterized by additional mutations in RNA splicing and epigenetic modifier genes. We observed diverging expression of multiple leukaemia‐associated genes as well as discrepant *ex vivo* drug responses, suggestive of discrete functional properties. Importantly, significant prognostication was observed only in female *FLT3*‐ITD‐mutated AML. Thus, we suggest optimization of *FLT3*‐ITD mutation status as a clinical tool in a sex‐adjusted manner and hypothesize that prognostication, prediction and development of therapeutic strategies in AML could be improved by including sex‐specific considerations.

## Graphical abstract

Internal tandem duplications (ITDs) in the FMS‐like tyrosine kinase 3 (*FLT3*) gene are linked to poor prognosis in acute myeloid leukaemia (AML). Here, we investigated sex‐related differences in mutation, gene expression and drug sensitivity profiles in *FLT3*‐ITD‐mutated AML. We demonstrated significant sex‐associated molecular and functional differences, and surprisingly identified significant prognostication related to *FLT3*‐ITD only in female AML patients.

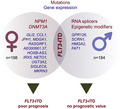

**Abbreviations**
AML: acute myeloid leukaemia
AR: allelic ratio
AUC: area under the curve
BM: bone marrow
CE: capillary electrophoresis
DGE: differential gene expression
FLT3: Fms‐like tyrosine kinase 3
ITD: internal tandem duplication
MDS: myelodysplastic syndrome
NGS: next‐generation sequencing
VAF: variant allele frequency

## 1 Introduction

Sex influences regulatory mechanisms of the haematopoietic system as well as innate and adaptive immune responses [1, 2]. Strong age‐ and sex‐specific discrepancies in incidence of autoimmune conditions [3] and cancer [4], including haematological malignancies [5], suggest fundamental genetic and endocrine sex‐related variability.

Androgens have been used to treat various bone marrow (BM) failure syndromes since the 1960s [6], and the presence of hormone receptors on haematopoietic cells, including malignant cell populations, has been recognized for decades [7]. Yet, little is known about the molecular mechanisms modulating haematopoiesis through sex hormone pathways or their contribution in haematopoietic malignancies. It has been indicated that sex hormone receptors significantly contribute in regulation of haematopoietic cell subsets, including stem and progenitor cells [8]. Sex disparity in acute myeloid leukaemia (AML) incidence is known, with a progressive male excess with increasing age [9]. It has also been shown that male AML patients have significantly inferior outcomes compared to females, both in adult and in paediatric AML [10]. Sex‐specific mutational profiles in AML have also been described; *FLT3*‐ITD, *NPM1* and *DNMT3A* mutations are overrepresented in females [11, 12], while mutations in *RUNX1*, *ASXL1*, *SRSF2*, *STAG2*, *BCOR*, *U2AF1* and *EZH2* are more prevalent in males [12]; and female overrepresentation among AML patients with co‐occurrence of *DNMT3A*, *NPM1* and *FLT3*‐ITD mutations has also been reported [13].

Despite the demographic differences in survival and somatic mutation profiles, sex‐specific considerations are currently not made in therapeutic assessment or clinical risk stratification. Among the mutations with reported sex‐dependent discrepancies are internal tandem duplications (ITDs) of the FMS‐like tyrosine kinase 3 (*FLT3*). *FLT3*‐ITD is present in approximately 25% of the AML cases and is a negative prognostic marker that is integrated in standard risk stratification guidelines in AML as well as guiding FLT3‐targeted therapy [11, 14]. Yet, the association between sex and *FLT3*‐ITD mutations has not been explored in depth. Here, we present results from genomic, functional and time‐to‐event‐analyses of four well‐annotated cohorts, including the Beat AML cohort [15], the LAML‐TCGA cohort [16] and two independent HOVON/SAKK cohorts, in sum comprising 1755 AML patients. Cohort compositions with regard to sex and *FLT3* mutation status are described in Table 1, and comparative analyses of sex differences related to clinical parameters are presented in Tables S1–S3.

**Table 1:** Composition of all cohorts in relation to sex, age and FLT3 status.

	Beat AML	HOVON1	HOVON2	LAML‐TCGA
Total				
All				
Number	496^a^	432	625	200
Median age (range)	61 (2–87)	46 (15–77)	53 (18–65)	57 (18–88)
Female				
Number	222	215	273	92
Median age (range)	57.5 (2–85)	46 (16–77)	53 (19–65)	57 (21–88)
Male				
Number	276	217	352	108
Median age (range)	63 (5–87)	46 (15–75)	55 (18–65)	58 (18–83)
ITD+				
All				
Number	123	117	146	39
Median age (range)	61 (10–85)	47 (18–77)	50 (18–65)	57 (22–83)
Female				
Number	62	67	74	18
Median age (range)	60 (22–79)	45 (18–77)	53 (19–65)	58.5 (25–68)
Male				
Number	61	50	72	21
Median age (range)	61 (10–85)	47.5 (19–71)	48.5 (18–65)	57 (22–83)
ITD−				
All				
Number	373	315	479	161
Median age (range)	62 (2–87)	45 (15–75)	55 (18–65)	57 (18–88)
Female				
Number	160	148	199	74
Median age (range)	55.5 (2–85)	46 (16–74)	52 (19–65)	57 (21–88)
Male				
Number	213	167	280	87
Median age (range)	64 (5–87)	45 (15–75)	65 (18–65)	58 (18–81)

^a^ The sample selection analysed from the Beat AML cohort comprises a total of 498 samples, but age for two of these samples is not annotated.

## 2 Methods

### 2.1 Patient sample selection

We analysed four independent patient cohorts: Beat AML, LAML‐TCGA, HOVON 1 and HOVON 2. The Beat AML and LAML‐TCGA cohorts were used for descriptive analyses of somatic variant profiles. The Beat AML cohort was further investigated by differential gene expression (DGE) analyses and exploration of *ex vivo* drug response profiles. All four cohorts were included for time‐to‐event analyses. Data from Beat AML [15] and LAML‐TCGA [16] are publicly available. All patients in the two HOVON cohorts provided written informed consent at trial inclusion.

The Beat AML sample cohort comprises 672 specimens from 562 individuals. We restricted our analysis to samples identified in the All.variants.csv file, downloaded from http://www.vizome.org/aml/geneset/ on the 01.11.2018. This included 608 samples from 519 individuals. At inclusion, 15 individuals had two samples acquired from different tissues. The sample with the lowest number of recurrent mutations was discarded. We filtered the remaining samples based on the variable ‘dxAtSpecimenAcquisition’ and retained samples annotated as ‘Acute myeloid leukaemia (AML) and related precursor neoplasms’, resulting in a total of 571 samples. For descriptive analysis of somatic variants, we included only the first sample when serial samples were present, resulting in 498 samples from 498 individuals. For DGE and drug response, we analysed 390 samples from 360 individuals and 359 samples from 322 individuals, respectively, including only samples with sample IDs overlapping with the exome sequencing data set (Fig. S1A). For outcome assessment, we restricted the analysis to diagnostic samples, denoted ‘Initial Acute Leukemia Diagnosis’, where survival data was present, resulting in 303 individuals (Fig. S1B). Notably, we constructed an extended *FLT3*‐ITD annotation based on the combination of the consensus *FLT3*‐ITD variable from the clinical summary table (Table S5‐Clinical Summary) and Pindel call of *FLT3*‐ITDs, as reported in the All.variants.csv file.

The HOVON 1 and HOVON 2 cohorts comprise (non‐APL) newly diagnosed AML patients aged 15–80 years treated on various study protocols of the Dutch‐Belgian Hemato‐Oncology Cooperative Group (HOVON) and the Leukaemia Group of the Swiss Group for Clinical Cancer Research (SAKK) during the period 1987 to 2013. The sample selection analysed here was restricted to treatment naïve non‐APL AML patients with known *FLT3*‐ITD status. This includes patients treated in the protocols HO04, HO04a [17], HO29 [18, 19], HO42 [20, 21], HO43 [22] and HO102 [23]. The studies are previously published, and sampling and data acquisition were performed as previously described [24, 25]. Patients included in HO102 are referred to as HOVON2, while the remaining patients comprise HOVON1. Detailed information on the individual trials is available at http://www.hovon.nl.

LAML‐TCGA is a strongly selected sample cohort, composed to cover the major cytomorphologic and cytogenetic groups recognized in AML prior to 2013 [16]. The cohort comprises 200 *de novo* AML patients, including 92 females and 108 males ranging from 18 to 88 years. Due to its selective and unrepresentative composition, we have mainly used this cohort for integrated survival analyses. Single cohort comparative analyses of sex differences related to clinical parameters were not performed. The TCGA‐AML data analysis is based exclusively on variables as presented in the file ‘SuppTable01.xlsx’, downloaded from https://gdc.cancer.gov/node/876. For survival analysis, the variable ‘OS months 3.31.12’ is used.

### 2.2 Ethics

All clinical trials were approved by local ethics committees and performed in accordance with the Declaration of Helsinki.

### 2.3 Statistics

For comparison of continuous variables, we applied the nonparametric Wilcoxon rank sum test/Mann–Whitney test. The Fisher exact test was applied for comparison of categorical data. For DGE analysis, we log‐transformed the CPM matrix provided in ‘Table S9‐Gene Counts CPM’ by the formula: CPM(log2+0.1). Analyses were performed in accordance with the Linear Models for Microarray Data pipeline in the bioconductor r package (limma version 3.38.3) [26]. For exploration of the drug sensitivity data from the Beat AML cohort, we analysed the area under the curve (AUC) values provided in ‘Table S10‐Drug Responses’, applying categories from ‘Table S11‐Drug Families’. Time‐to‐event analyses were performed by generation of Kaplan–Meier survival curves and compared for differences using the log‐rank test. For continuous variables, impact on outcome was evaluated by univariate Cox models followed by multivariate logistic regression models. *P*‐values were adjusted by the Benjamini–Hochberg method, and threshold was set at *P* ≤ 0.05. All statistical analyses were performed in r‐studio (version 1.1.453) and r (version 3.5.0) (R Foundation for Statistical Computing, Vienna, Austria). Graphs were made with ggplot2 (version 3.1.0, https://ggplot2.tidyverse.org) [27] and figures made in Adobe illustrator CS6 (version 16.0.0. Adobe Inc., San Jose, CA, USA).

## 3 Results

### 3.1 Comparative genomic architecture

**Fig. 1:**
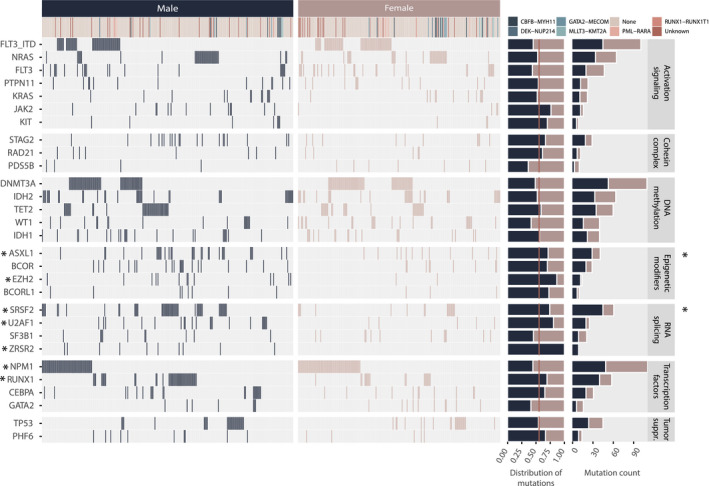
Sex‐separated overview of genes mutated in more than 2% (≥ 10 patients) of the subsampled Beat AML sample cohort (*n* = 498), identified by analysing the final curated exome sequencing variant list downloaded at http://www.vizome.org/aml/geneset/. Of note, the result of the *FLT3* gene is separated by ITD and non‐ITD *FLT3* mutations on separate rows. In all other analyses in this report, *FLT3*‐ITD annotations include additional samples where *FLT3*‐ITDs were identified exclusively by conventional methods. * indicates single genes (left panel) and gene classes (right panel) with a significantly different mutation frequency between male and female patients. Statistical significance was evaluated using the Fisher exact test, *P > 0.05.

To provide a context for sex‐related variant patterns, we compared the sex‐related distribution of somatic variants annotated in the Beat AML cohort. We found that the number of somatic variants did not differ significantly across sexes, although male individuals tended to have higher numbers (Fig. S2A). Comparing the number of highly recurrently mutated genes (mutated in > 2% of the cohort), no significant differences were observed (Fig. S2B). There were no sex‐related differences within the *FLT3*‐ITD‐mutated subgroup. We identified 28 highly recurrently mutant genes, of which 23 genes are autosomal and five are X‐linked, including *BCOR*, *BCORL1*, *PHF6*, *STAG2* and *ZRSR2*. The frequency of *FLT3* (F: 82/222, M: 79/275) and *DNMT3A* (F: 57/222, M: 54/276) mutations was higher in females, although not statistically significant. However, seven other genes were significantly different; *NPM1* (F: 62/222, M: 50/275, *P* = 0.01) was overrepresented in females, while *RUNX1* (F: 18/222 vs M: 41/275, *P* = 0.025), *ZRSR2* (F: 0/222, M: 10/275, *P* = 0.003), *SRSF2* (F: 16/222, M: 46/275, *P* = 0.002), *U2AF1* (F: 5/222, M: 21/275, *P* = 0.008), *ASXL1* (F: 12/222, M: 30/275, *P* = 0.035) and *EZH2* (F: 2/222, M: 13/275, *P* = 0.016) were overrepresented in males (Fig. 1). Notably, six of these seven genes are autosomal.

When expanding the selection to genes mutated in > 1% of the total Beat AML selection, we found mutations in *ZNF711* (F: 5/222, M: 0/276, *P* = 0.0172) exclusively in females, while *BRCC3* (F: 0/222, M: 6/276, *P* = 0.0358) and *SMC3* (F: 1/222, M: 8/276, *P* = 0.047) were both significantly overrepresented in males (Fig. S3 and Table S4A). Within the *FLT3*‐ITD‐positive group, we identified mutations in both *BCOR* (F: 0/62, M: 4/61, *P* = 0.057) and *CCND3* (F: 0/62, M: 4/61, *P* = 0.057) only in male *FLT3*‐ITD AML (Table S4B). Interestingly, the *FLT3*‐ITD‐negative male and female groups were significantly different: while mutations in *WT1* (F: 19/160, M: 11/215, *P* = 0.0207) were significantly more frequent in females, *RUNX1* (F: 9/160, M: 31/215, *P* = 0.0066), *SRSF2* (F: 14/160, M: 40/215, *P* = 0.0074), *U2AF1* (F: 2/160, M: 15/215, *P* = 0.0102), *ZRSR2* (F: 0/160, M: 7/215, *P* = 0.0219) and *EZH2* (F: 2/160, M: 12/215, *P* = 0.0298) were significantly characteristic of males (Table S4C). This pattern is largely attributable to the excess of male samples annotated as ‘transformed’ (from a prior haematological malignancy). Excluding these cases, only the relationship between male sex and *SRSF2* (F: 11/197, M: 28/215, *P* = 0.0112) and *U2AF1* (F: 4/197, M: 14/215, *P* = 0.0301) remained significant. Additionally, *MXRA5* (F: 5/197, M: 0/215, *P* = 0.0243) and *NF1* (F: 5/197, M: 0/215, *P* = 0.0243) were identified as more frequently mutated in females, while *PHF6* (F: 2/197, M: 10/215, *P* = 0.0378) was significantly overrepresented in males (Table S4D).

For comparison, the sex‐related distribution of somatic variants in the LAML‐TCGA cohort is presented in Table S5. Due to the selective composition of this cohort, integrated analyses of somatic variants were not performed.

The 28 recurrently mutated genes in the Beat AML cohort were subsequently categorized by gene product functional properties: DNA methylation, activating signalling, transcriptional regulation, tumour suppressor function, epigenetic modification, cohesion complex regulation and RNA splicing. We found that 14% of female vs 31% of male individuals had at least one mutation in one of the four RNA splicing genes: *SRSF2*, *U2AF1*, *SF3B1* and *ZRSR2* (F: 32/222, M: 85/275, *P* = 1.847e‐05, adj *P* = 0.0001). Similarly, 10% female vs 23% male individuals had at least one mutation in one of the four epigenetic modifier genes: *ASXL1*, *BCOR*, *EZH2* and *BCORL1* (F: 20/222, M: 60/275, *P* = 0.0001, adj *P* = 0.0004). The relationships remained significant in the nontransformed group for both RNA splicing genes (F: 23/197, M: 52/215, *P* = 0.0013, adj *P* = 0.009) and epigenetic modifier genes (F: 15/197, M: 35/215, *P* = 0.01, adj *P* = 0.03). The association was also significant within the *FLT3*‐ITD‐negative group, where both epigenetic modifier genes (F: 22/160, M: 65/215, *P* = 0.0001, adj *P* = 0.001) and RNA splicing genes (F: 18/160, M: 50/215, *P* = 0.002, adj *P* = 0.009) remained significantly associated with males. In the *FLT3*‐ITD‐positive group, the same trend was apparent (epigenetic modifiers – F: 2/62, M: 11/61, *P* = 0.0085, RNA splicing genes – F: 10/62, M: 20/61, *P* = 0.037), although not significant by adjusted *P*‐value (Table S6; Fig. S4).

We note that none of the sex discrepancies in somatic mutations of *single genes* reported as statistically significant here were significant by adjusted *P*‐value. However, many of our findings, including the association of *FLT3*‐ITD, *NPM1* and *DNMT3A* mutations in females and *RUNX1*, *ASXL1*, *SRSF2*, *STAG2*, *U2AF1* and *EZH2* mutations in males, have been reported by others [11, 12, 13]. Furthermore, when categorizing individual genes by functional gene class, the findings are significant by adjusted *P*‐value. This significance is driven by the same individual genes significant by nonadjusted *P*‐value. Therefore, findings on the single gene level are also reported here.

We further explored the distribution of mutations of the frequently *FLT3*‐ITD co‐mutated genes *DNMT3A* and *NPM1*. As this information was available for all four cohorts, integrated analysis was performed. Mutation status of all three genes was known in 1624 cases. We found that significantly more females than males had a mutation in at least one of these three genes (F: 401/748, M: 397/876, *P* = 0.001). The combination of *FLT3*‐ITD and *NPM1* mutation without *DNMT3A* mutation (F: 56/748, M: 40/876, *P* = 0.015, adj *P* = 0.04) and the combination of *FLT3*‐ITD, *NPM1* and *DNMT3A* mutation (F: 70/74, M: 43/876, *P* = 0.0006, adj *P* = 0.004) were both significantly associated with females (Table S7; Fig. S5).

We subsequently compared the distribution of variant allele frequencies (VAF) of the genes mutated at least 10 times in the Beat AML cohort and found that VAF was significantly higher in male compared to female individuals in five genes. As expected, this included the X‐linked genes *STAG2, PHF6* and *BCOR*, but also the autosomal gene *ASXL1*. *BCORL1*, despite being X‐bound, did not have significantly higher VAF in males. *ZRSR2* was mutated exclusively in males (Fig. S6).

*FLT3* was not among the genes identified with significantly different VAF across the sexes. It has previously been shown that the allelic ratio (AR) of *FLT3*‐ITD mutations has prognostic implications [28, 29]. To investigate whether there were sex discrepancies in *FLT3*‐ITD AR among these patients, we calculated the AR from the VAF (the approach is described in the figure legend of Fig. S7). We note that although there is no standardized approach to measure *FLT3*‐ITD AR, it is commonly measured by DNA fragment analysis by capillary electrophoresis (CE) [30, 31]. Here, we have used the VAF from next‐generation sequencing (NGS) analyses to calculate AR, as it has previously been shown that there is high concordance between CE and NGS assays in measuring *FLT3* mutational burden in AML patients [32]. As was the case for VAF, we did not find significant differences in AR between males and females in this cohort (Fig. S7).

### 3.2 Gene expression analysis

**Fig. 2:**
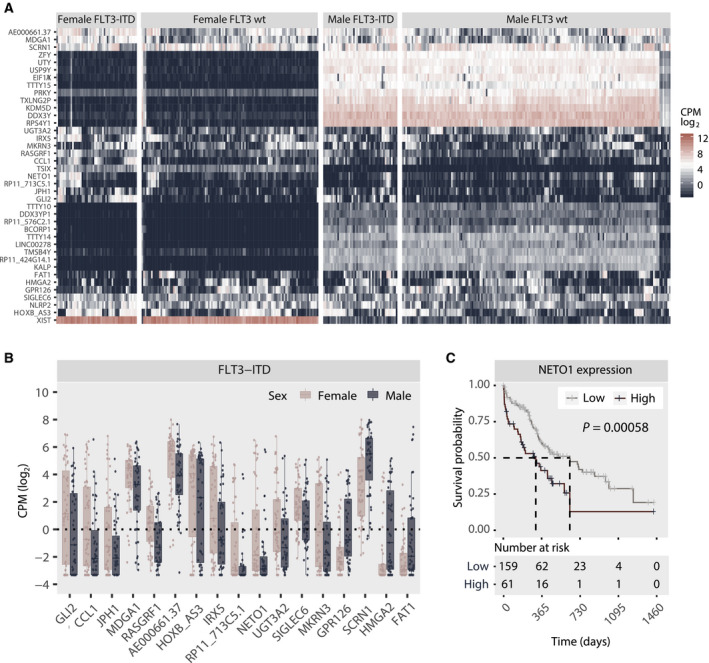
(A) Heatmap depicting unsupervised clustering of CPM‐log2 values of all transcripts differentially expressed between female and male *FLT3*‐ITD‐positive individuals. Clustering was done by columns and rows and then manually faceted by *FLT3*‐ITD status and sex. (B) Pairwise comparison of the 17 transcripts identified as differentially expressed between female and male *FLT3*‐ITD‐positive samples based on the results of the DGE analysis, excluding samples also significantly different in the *FLT3*‐wt subgroup. Only statistically significant results are shown. Statistical significance was evaluated using the Wilcoxon rank sum test/Mann–Whitney test. Each dot represents an individual specimen, and the box plot graphically presents the median and the spread. The lower and upper hinges correspond the 25th and 75th percentiles, respectively, and the upper and lower whisker extends to the largest and smaller values (no further than 1.5 times the total interquartile range from the hinges). Only *FLT3*‐positive samples are presented. The same comparison for *FLT3*‐ITD‐negative samples is available in Fig. S9. (C) Kaplan–Meier curve comparing the outcome of patients characterized by high (*n* = 61) and low (*n* = 159) mRNA expression (log2 CPM) of *NETO1*. Statistical significance is evaluated using the log‐rank test. *P* = 0.00058.

Based on the sex‐related pattern of *FLT3*‐ITD and co‐mutations, we questioned whether there was sex disparity in gene expression in the *FLT3*‐ITD‐mutated group. We analysed 380 specimens in the Beat AML cohort, comprising 163 female and 217 male samples, respectively, of which 51 female and 47 male were *FLT3*‐ITD‐positive samples. We identified a total of 39 differentially expressed genes: 16 upregulated and 23 downregulated in the female *FLT3*‐ITD‐positive subgroup, including 14 Y‐linked, 2 X‐linked, 7 nonannotated and 16 autosomal genes (Fig. 2A; Table S8). Subtracting genes also differentially expressed in the *FLT3*‐ITD‐negative group, we identified a total of 17 mRNA transcripts mapped to 16 genes, of which *GLI2, CCL1, JPH1, MDGA1, RASGRF1, AE000661.37, HOXB‐AS3, IRX5, NETO1/RP11‐713C5.1, UGT3A2, SIGLEC6* and *MKRN3* were all significantly higher expressed in *FLT3*‐ITD‐positive females, while *GPR126, SCRN1, HMGA2* and *FAT1* were significantly higher expressed in *FLT3*‐ITD‐positive males (Fig. 2B; Figs S8 and S9). Comparing *FLT3*‐ITD‐positive (*n* = 98) and *FLT3*‐ITD‐negative specimens (*n* = 282) irrespective of sex, we found that *GLI2, CCL1, JPH1, MDGA1, RASGRF1, AE000661.37, HOXB‐AS3, IRX5* and *RP11‐713C5.1* were all significantly upregulated in *FLT3*‐ITD‐positive AML, while *GPR126* was significantly downregulated.

To explore the functional relevance of genes identified as differentially expressed, we explored their inter‐relationship with disease outcome in the 220 individuals in the Beat AML cohort where survival and expression data were available. By univariate analysis, five of the 39 differentially expressed genes were identified as significantly associated with outcome (Table S9): *NETO1*, *TMSB4Y*, *TTTY10*, *HMGA2* and *FAT1*. In multivariate analysis including these five transcripts, only *NETO1* remained significant (Fig. S10). High expression of *NETO1* significantly correlated with poor outcome (Fig. 2C). Stratifying the patients by *FLT3*‐ITD status and sex, we found that high expression of *NETO1* was significantly associated with poor prognosis in both *FLT3*‐ITD‐positive and *FLT3*‐ITD‐negative patients and in both sexes, although a stronger negative correlation was observed in the male subpopulation (Fig. S11). Analyses splitting the patients by both FLT3‐ITD status and sex were not performed due to small effect sizes.

### 3.3 Drug sensitivity and resistance testing

To further explore the functional consequences of the sex‐discrepant leukemic architecture, we assessed the variation in drug sensitivity profiles in the Beat AML cohort (Table S10). We focused on the 113 compounds tested in a minimum of 100 specimens, and samples overlapping with our previous analyses, resulting in 348 specimens from 311 individuals. 96/113 compounds were annotated by mechanism of action and categorized into one or more of 39 different groups (Fig. S12).

Comparing drug sensitivity across the 39 various groups between female and male individuals irrespective of ITD status, we identified a weak but significant relationship between RTK‐RET inhibitors (1/39) and higher sensitivity in females. When comparing *FLT3*‐ITD‐positive cases, six inhibitor families differed significantly: PI3K‐AKT‐MTOR, PI3K‐MTOR, RTK‐ERBB, RTK‐INSR‐IGF1R, SYK and CAMK inhibitors all demonstrated higher sensitivity in male samples (Fig. S13). Comparing female and male *FLT3*‐ITD‐negative specimens, only MEK inhibitors were significantly different, and more potent in female samples.

**Fig. 3:**
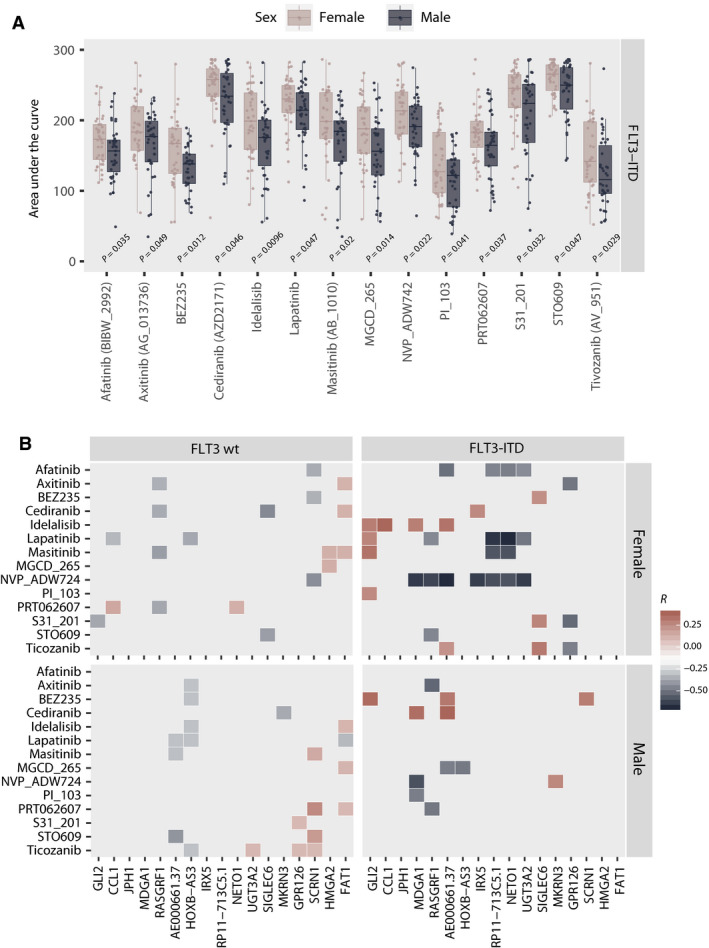
(A) Comparison of AUC between *FLT3*‐ITD‐mutated female and male specimens for the 14 compounds identified to demonstrate significant sex‐related divergence. The same comparison for *FLT3*‐ITD‐negative samples is available in Fig. S13. Statistical significance (*P* < 0.05) was evaluated using the Wilcoxon rank sum test/Mann–Whitney test. *P*‐values for each pairwise comparison are indicated in the plot. Additional statistical results for all compounds included in the analysis as well as sample size for all compared groups are reported in Tables S10 and S11. (B) Heatmap graphically presenting the pairwise correlation between the CPM‐log2 values of the differentially expressed genes and the AUC distribution of compounds identified as differentially potent across female and male *FLT3*‐ITD‐positive samples. The heatmap represents the correlation coefficient. Only statistically significant correlations are plotted.

When examining individual compounds, we identified five compounds with significantly sex‐discrepant potency, irrespective of *FLT3*‐ITD status: Females were more sensitive to AT7519, palbociclib and quizartinib (AC220), while males were more sensitive to cediranib (AZD2171) and pazopanib (GW786034). Further, we identified three compounds that were significantly more potent in female *FLT3*‐ITD‐negative samples, including AT7519, palbociclib and trametinib (GSK1120212). Importantly, we identified 14 inhibitors that demonstrated significantly lower potency in female *FLT3*‐ITD‐positive samples, with an overrepresentation of inhibitors targeting tyrosine kinase receptors and downstream targets known to be influenced by FLT3‐ITD signalling, including idelalisib, BEZ235, MGCD‐265, masitinib (AB 1010), NVP‐ADW742, tivozanib (AV‐951), S31‐201, afatinib (BIBW‐2992), PRT062607, PI‐103, cediranib (AZD2171), lapatinib, STO609 and axitinib (AG‐013736) (Fig. 3A; Table S11; Fig. S14).

To assess whether variation in gene expression correlated with variation in drug sensitivity, we further examined the pairwise relationships of the differentially expressed genes and the compounds with sex‐discrepant potency in *FLT3*‐ITD‐positive samples. We found that sensitivity to NVP‐ADW742 correlated with increasing gene expression of 7/13 RNA transcripts identified as upregulated in female *FLT3*‐ITD‐positive AML. Conversely, increasing *GLI2* expression correlated with reduced sensitivity to 4/14 drugs that were less potent in female *FLT3*‐ITD‐positive AML (Fig. 3B).

### 3.4 Survival

Finally, we investigated the relationships between sex and the prognostic strength of *FLT3*‐ITD mutation status on all cohorts combined as well as separately (Table S12a–e). HOVON 1 (*n* = 432) had a median follow‐up of 113.7 months, while HOVON 2 (*n* = 625) had a median follow‐up of 42.3 months. The Beat AML cohort had a short median follow‐up of 15.2 months. For survival analyses, only newly diagnosed cases were included (*n* = 303). LAML‐TCGA (*n* = 200) had a median follow‐up 47.2 months.

**Fig. 4:**
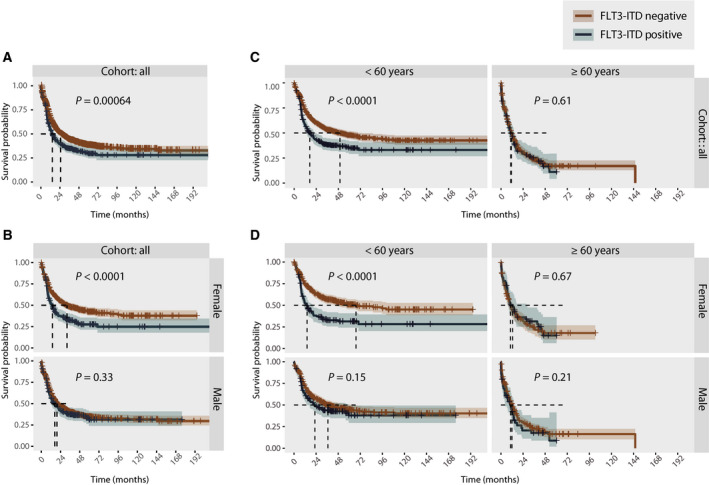
(A) Kaplan–Meier curve comparing the outcome of *FLT3*‐ITD‐mutated (*n* = 382) and non‐*FLT3*‐ITD‐mutated (*n* = 1178) patients across the four cohorts analysed together. (B) Kaplan–Meier curve comparing the outcome of *FLT3*‐ITD‐mutated and non‐*FLT3*‐ITD‐mutated patients analysed across the four cohorts but analysed individually for females (*FLT3*‐ITD negative *n* = 523; *FLT3*‐ITD positive *n* = 198) and males (*FLT3*‐ITD negative *n* = 655; *FLT3*‐ITD positive *n* = 184). (C) Kaplan–Meier curve comparing the outcome of *FLT3*‐ITD‐mutated and non‐*FLT3*‐ITD‐mutated patients across the four cohorts analysed together, separated by age (*FLT3*‐ITD‐negative patients: > 60 years *n* = 788; ≥ 60 years *n* = 390. *FLT3*‐ITD‐positive patients: > 60 years *n* = 278; ≥ 60 years *n* = 104). (D) Kaplan–Meier curve comparing the outcome of *FLT3*‐ITD‐mutated and non‐*FLT3*‐ITD‐mutated patients across the four cohorts combined, separated by sex and age (female FLT3‐ITD negative > 60 years *n* = 379; ≥ 60 years *n* = 144, female FLT3‐ITD positive > 60 years *n* = 143; ≥ 60 years *n* = 55; male FLT3‐ITD negative > 60 years *n* = 409; ≥ 60 years *n* = 246, male FLT3‐ITD positive > 60 years *n* = 135; ≥ 60 years *n* = 49). Statistical significance is evaluated using the log‐rank test. *P*‐values are indicated in each individual plot.

Despite *FLT3*‐ITD status being a recognized negative prognostic marker in AML, we found a significant association to poor outcome only in the HOVON 1 cohort (Fig. S15). When overall survival was analysed in the cohorts combined (*n* = 1560), we found as expected that *FLT3*‐ITD mutation status was associated with significantly lower survival (Fig. 4A). However, when survival analysis for all cohorts combined was split by sex, the significant prognostic association of *FLT3*‐ITD remained only in the female subpopulation (Fig. 4B). Analysing the cohorts independently, the same observation was apparent; *FLT3*‐ITD was significantly associated with poor outcome only in female patients in the HOVON1 cohort, and the same trend was observed in the HOVON2, Beat AML and LAML‐TCGA cohorts, although not significant (Figs S15 and S16; Table S12a–e).

A recent report suggests that the prognostic strength of *FLT3*‐ITD is age‐dependent [11]. Therefore, we performed age‐adjusted survival analyses, dichotomizing the population using a cut‐off of 60 years. Similarly, we found a significant association of *FLT3*‐ITD mutations with poor overall survival in the younger population only (> 60 years) (Fig. 4C). However, when the analysis was split by sex, we found an association between younger age and poor overall survival in the female subpopulation only. In the male subpopulation, there was no significant difference in overall survival between *FLT3*‐wt‐ and *FLT3*‐ITD‐mutated patients in the young (> 60 years) nor older (≥ 60 years) patients (Fig. 4D).

## 4 Discussion

In this work, we demonstrated sex disparity in somatic variant composition, gene expression and *ex vivo* drug response patterns in *FLT3*‐ITD‐mutated AML cases across four well‐characterized AML cohorts. *FLT3*‐ITD mutation status is integrated in standard risk stratification guidelines in AML. Yet, in the datasets we explored, *FLT3*‐ITD mutation status separated the disease outcome only in female AML. This may indicate that the prognostic utility of *FLT3*‐ITD mutation status is different across sexes. Of note, the prognostic value of *FLT3*‐ITD mutations is reportedly influenced by the mutational burden. Several studies have shown that a high *FLT3*‐ITD AR is linked to poor prognosis [28, 29]. Furthermore, it has also been shown that AML patients with a high *FLT3*‐ITD AR lacking *NPM1* mutations have a worse prognosis [33]. In the latter study, an excess of males was reported in this subgroup, although no statistically significant sex disparity was observed. In our study, we did not identify *FLT3*‐ITD VAF nor AR as significantly different across sexes, suggesting that a difference in the mutational burden of *FLT3* is likely not the cause of the sex discrepancies observed in outcome.

Another plausible explanation is related to discrepant distribution of age, disease presentation pattern and poor risk molecular features. We identified significant differences in the distribution of co‐mutations within the *FLT3*‐ITD‐positive subgroup. While males more frequently present with somatic variants in epigenetic modifier genes and/or RNA splicing genes, co‐mutations of *FLT3*‐ITD, *NPM1* and *DNMT3A* were overrepresented in females. The mutation pattern characterizing female patients in this subgroup is previously shown to be associated with adverse prognosis in *FLT3*‐ITD‐mutated AML [34]. Importantly, significant differences were also observed within the *FLT3*‐ITD‐negative subgroup, characterized by an abundance of *WT1* mutations in female specimens contrasted by overrepresentation of mutations in *RUNX1*, *SRSF2*, *U2AF1*, *ZRSR2* and *EZH2* in the male subgroup. These differences are most pronounced in the Beat AML cohort, where the mutated genes dominating the male *FLT3*‐ITD‐negative subpopulation are associated with poor outcome and with myelodysplastic syndrome (MDS) and secondary AML [35]. Whether this relationship represents inclusion asymmetry or a natural distribution is unknown. A characterization of the population‐based Swedish Acute Leukemia Registry, however, suggested that AML with an antecedent haematological disease is more frequent in males [36]. Similar to AML, there is female excess of MDS among younger individuals, and men with MDS have comparably inferior outcome [9]. Additionally, mutations in several genes overrepresented in males in the Beat AML cohort, including *SRSF2* and *U2AF1*, are reportedly overrepresented in male MDS [37].

Sex disparity in AML demography is well known, with a progressive male excess with increasing age [9]. As the male population is generally older, one could speculate whether older age is a contributing factor to the inferior outcome of male patients, within both the *FLT3*‐wt‐ and *FLT3*‐ITD‐mutated subgroups. Specifically, age differences could potentially result in sex disparities of treatment (i.e. proportion of patients fit to receive intensive chemotherapy and/or allogeneic haematopoietic stem cell transplantation), which might be an important confounder for this study. In the HOVON cohorts, however, we found no significant differences between males and females in the proportion of transplanted patients (Table S2). Due to insufficient annotation across the cohorts, additional effects of treatment were unfortunately not evaluated in this study, and the impact of such therefore remains uncertain. Interestingly, it was recently shown that the prognostic impact of *FLT3*‐ITD mutations is also age‐dependent, with poor overall survival observed in younger *FLT3*‐ITD‐mutated (< 60 years) AML patients, but not within the older population (60–74 years) [11]. We corroborate this observation in age‐adjusted survival analyses in this study. Interestingly, when splitting the analysis by sex, this finding was significant only within the female subpopulation, further emphasizing the poor prognostic association between female sex and *FLT3*‐ITD mutations.

The sex‐specific age distribution characterizing AML demography suggests that cohort composition is an important confounder. The age composition of the Beat AML cohort resembles the reported demographic distribution of AML far better than the strongly selected LAML‐TCGA cohort, in part explaining the lack of sex‐biased mutations in this cohort. One could argue that age‐matching and/or use of a population‐based sample selection is the optimal condition for comparison. What remains, however, is to identify the cell‐intrinsic and cell‐extrinsic mechanisms underlying the sex disparity in AML incidence and molecular presentation. We hypothesize a significant contribution of sex‐specific leukaemia‐host interactions related to disease development. Mutations are stochastic events, and there are thus no apparent reasons to suggest that male and female haematopoietic stem cells acquire discrete mutations. What is plausible, however, is that sex is an important contextual contributor in determining the translational effect in comparative fitness of novel gene variants. Interestingly, one of the very first papers characterizing the FLT3 receptor suggested FLT3‐mediated regulatory function not only in the haematopoietic compartment but also in the gonads, placenta and brain [38]; tissues characterized by sexual dimorphism. This suggests that downstream effects of FLT3 signalling may be influenced by sex variation. This hypothesis has been substantiated by studies demonstrating functional relevance of FLT3 expression in germinal tissue [39, 40] and by investigation of the oestrogen receptor in FLT3‐positive haematopoietic cells, progenitor cells and mature cellular subsets like dendritic cells [8, 41]. Together, these observations suggest inter‐regulatory pathways between sex steroid receptors and FLT3.

The DGE analysis showed distinct expression of leukaemogenesis‐associated genes, suggesting functionally relevant cell‐intrinsic sex‐dependent differences. Of the genes more highly expressed in female *FLT3*‐ITD‐positive AML, the hedgehog signalling mediator *GLI2* has been associated with *FLT3*‐ITD‐mutated leukaemia [42], while *HOXB‐AS3* is reportedly upregulated in *NPM1*‐mutated AML, and has been implied to contribute in regulation of AML cell‐cycle progression [43]. NETO1, however, the only differentially expressed gene with prognostic impact in this cohort, is predominantly studied in the central nervous system where it is found to regulate kainate receptor signalling [44], and a role in haematopoiesis has to our knowledge not been investigated. The significance of this finding is therefore uncertain. Of the genes more highly expressed in male *FLT3*‐ITD‐positive AML, *FAT1* was previously shown to be somatically mutated in *FLT3*‐ITD‐positive AML and significantly in combination with *NPM1* and *DNMT3A* [45]. This study also showed that *FAT1* exerted tumour suppressor activity specifically in *FLT3*‐ITD‐positive AML, perhaps providing a partial explanation for the lack of prognostic impact of *FLT3*‐ITD in this subpopulation.

The sum of the *ex vivo* drug response patterns and clinical outcome analysis suggests that response to therapeutic intervention in *FLT3*‐ITD‐mutated AML may be influenced by sex. Interestingly, this is in line with observations from the phase III clinical trials RATIFY and QuANTUM‐R, treating *FLT3*‐ITD‐positive *de novo* AML with FLT3‐targeting inhibitors (midostaurin and quizartinib, respectively), both reporting significant survival benefit in the male subpopulation only [14, 46]. Unfortunately, neither of these trials were powered to address potential sex differences in therapeutic response, and additional trials would be necessary to validate this observation. Our *ex vivo* drug sensitivity analyses did not, however, show a similar sex difference in the sensitivity to FLT3 inhibitors. It has previously been shown that *FLT3*‐ITD dependency in *ex vivo* assays is contingent on the availability of growth factors, and thus, the response to FLT3‐targeting drugs is permissive of the culture conditions [47]. This could provide a partial explanation for this discrepancy. However, the long incubation time is also an important confounder for *ex vivo* drug screens, and conclusions regarding the translational value of such data should be drawn with caution. Notably, sex‐related *ex vivo* drug response patterns have also been reported in the study of the Cancer Cell Line Encyclopedia [48] and in clinical trials of the tyrosine kinase inhibitor sunitinib in other malignancies [49]. Thus, these observations may be of great importance for the field of precision haemato‐oncology, as novel targeted therapies may benefit female and male individuals differently. Although men and women in an unstratified AML population have similar prospects, this picture may be significantly different within discrete molecularly defined strata.

The impact of sex on disease presentation and outcome is likely a composite picture involving multiple factors, including societal influence and socialization (e.g. risk behaviours, occupation, stress and inclination to seek health care). Most importantly, however, sex is associated with major physiological differences, the impact of which has been insufficiently explored in haematology and in medicine in general. The field of cardiovascular disease is a noteworthy exception, where sex disparity is now widely recognized and where sex considerations are becoming integrated into research, diagnostics and clinical disease management [50, 51]. Similarly, we hypothesize that including sex‐specific considerations in preclinical and clinical research in AML could advance the pathophysiological understanding, which could ultimately lead to more precise prognostication and improved therapeutic options for these patients.

## 5 Conclusions

Assessment of mutation status is becoming an integrated part of the diagnostic characterization of adult AML patients. This information is subsequently incorporated into risk stratification models and clinical decision‐making. *FLT3*‐ITD mutation status is well established as a biomarker in AML. However, important questions remain regarding its optimal application and utility. Our observations suggest that *FLT3*‐ITD mutation status could be optimized as a clinical tool in a sex‐adjusted manner. Furthermore, we suggest that sex‐specific considerations should be considered in preclinical and clinical experimental design and biomarker analyses in AML. Sex should represent an independent stratification factor when randomizing to clinical trials, and be systematically included when analysing and reporting on clinical data in AML. We hypothesize that addressing sex‐related regulation of molecularly defined subgroups of AML could advance pathophysiological understanding, perhaps ultimately revealing new therapeutic possibilities.

## Conflict of interest

The authors declare no conflict of interest.

## Author contributions

The study was designed by CE and BTG. CE analysed and interpreted data, prepared figures and wrote the paper. MH interpreted data, prepared figures and wrote the paper. TG, BL and PJMV collected data from the HOVON cohorts and contributed with data interpretation. All authors contributed in manuscript preparation.

### Peer Review

The peer review history for this article is available at https://publons.com/publon/10.1002/1878‐0261.13035.

## Supporting information

**Fig. S1.** Overview of the Beat AML sample selection analysed.
**Fig. S2.** Distribution of somatic variants in the Beat AML sample selection.
**Fig. S3.** Sex‐specific distribution of somatic mutations in the Beat AML cohort.
**Fig. S4.** Age‐ and sex distribution of samples with somatic mutations, presented by gene class.
**Fig. S5.** Sex‐specific distribution of comutations of *FLT3‐ITD, NPM1* and *DNMT3A* in the HOVON1, HOVON2, Beat AML and LAML‐TCGA cohorts.
**Fig. S6.** Sex‐specific distribution of VAF of mutations detected in a minimum of 10 samples in the Beat AML sample selection.
**Fig. S7.** Sex‐specific distribution of *FLT3*‐ITD allelic ratio.
**Fig. S8.** Expression level of genes identified as differentially expressed between male and female *FLT3*‐ITD‐positive samples.
**Fig. S9.** Pairwise comparison of gene expression in *FLT3*‐ITD and non‐FLT3‐ITD samples in genes identified as differentially expressed between male and female *FLT3*‐ITD‐positive samples.
**Fig. S10.** Cox Proportional‐Hazards model including the five genes where expression was identified as significantly correlated with outcome by univariate analysis.
**Fig. S11.** Kaplan–Meier curves comparing patients with high and low expression of *NETO1*, split by *FLT3*‐ITD mutation status and sex.
**Fig. S12.** Overview of drugs and drug classes.
**Fig. S13.** Comparison of drug sensitivity scores between *FLT3*‐ITD‐mutated male and female samples, presented by drug class.
**Fig. S14.** Comparison of drug sensitivity scores of drugs identified with significantly different potency in male and female *FLT3*‐ITD‐mutated samples.
**Fig. S15.** Kaplan–Meier curves comparing the outcome of male and female *FLT3*‐ITD and *FLT3*‐wt patients in the HOVON1, HOVON2, Beat AML and LAML‐TCGA cohorts.
**Fig. S16.** Kaplan‐Meier curve comparing the outcome of female and male patients across the four cohorts separated by FLT3‐ITD mutation status.
**Table S1.** (A) Cohort Composition – Beat AML sample cohort (all). (B) Cohort Composition – Beat AML sample cohort (no *FLT3*‐ITD). (C) Cohort Composition – Beat AML sample cohort (*FLT3*‐ITD). (D) Cohort Composition – Beat AML sample cohort (all) split by *FLT3*‐ITD.
**Table S2.** Cohort Composition HOVON1.
**Table S3.** Cohort Composition HOVON2.
**Table S4.** (A) Somatic mutations in the Beat AML sample cohort (all). (B) Somatic mutations in the Beat AML sample cohort (*FLT3*‐ITD). (C) Somatic mutations in the Beat AML sample cohort (no *FLT3*‐ITD). (D) Somatic mutations in the Beat AML sample cohort (not transformed).
**Table S5.** Somatic mutations in the LAML‐TCGA cohort (all).
**Table S6.** Mutations in Beat AML cohort categorized by gene product function.
**Table S7.***FLT3*‐ITD, *DNMT3A* and *NPM1* comutations in the Beat AML, LAML‐TCGA HOVON1 and HOVON2 cohorts.
**Table S8.** Beat AML cohort – Differential gene expression analysis.
**Table S9.** Univariate Cox regression analysis of differentially expressed genes in the Beat AML cohort.
**Table S10.** Number of *FLT3*‐ITD and *FLT3*‐ITD wt male and female samples screened for each drug in the in drug screen.
**Table S11.** P‐values for comparison of drug sensitivity.
**Table S12.** (a) Survival analysis of all cohorts combined. (b) Survival analysis of the Beat AML cohort. (c) Survival analysis of the LAML‐TCGA cohort. (d) Survival analysis of the HOVON1 cohort. (e) Survival analysis of the HOVON2 cohort.
