# Supplementary material for: Sex disparity in acute myeloid leukaemia with FLT3 internal tandem duplication mutations: implications for prognosis
Source: Mol Oncol. 2021 Jun 20;15(9):2285–99. doi: 10.1002/1878-0261.13035 (PMC8410575; doi:10.1002/1878-0261.13035)
Supplement: Supplementary file 1 — Fig. S1. Overview of the Beat AML sample selection analysed. Fig. S2. Distribution of somatic variants in the Beat AML sample selection. Fig. S3. Sex‐specific distribution of somatic mutations in the Beat AML cohort. Fig. S4. Age‐ and sex distribution of samples with somatic mutations, presented by gene class. Fig. S5. Sex‐specific distribution of comutations of FLT3‐ITD, NPM1 and DNMT3A in the HOVON1, HOVON2, Beat AML and LAML‐TCGA cohorts. Fig. S6. Sex‐specific distribution of VAF of mutations detected in a minimum of 10 samples in the Beat AML sample selection. Fig. S7. Sex‐specific distribution of FLT3‐ITD allelic ratio. Fig. S8. Expression level of genes identified as differentially expressed between male and female FLT3‐ITD‐positive samples. Fig. S9. Pairwise comparison of gene expression in FLT3‐ITD and non‐FLT3‐ITD samples in genes identified as differentially expressed between male and female FLT3‐ITD‐positive samples. Fig. S10. Cox Proportional‐Hazards model including the five genes where expression was identified as significantly correlated with outcome by univariate analysis. Fig. S11. Kaplan–Meier curves comparing patients with high and low expression of NETO1, split by FLT3‐ITD mutation status and sex. Fig. S12. Overview of drugs and drug classes. Fig. S13. Comparison of drug sensitivity scores between FLT3‐ITD‐mutated male and female samples, presented by drug class. Fig. S14. Comparison of drug sensitivity scores of drugs identified with significantly different potency in male and female FLT3‐ITD‐mutated samples. Fig. S15. Kaplan–Meier curves comparing the outcome of male and female FLT3‐ITD and FLT3‐wt patients in the HOVON1, HOVON2, Beat AML and LAML‐TCGA cohorts. Fig. S16. Kaplan‐Meier curve comparing the outcome of female and male patients across the four cohorts separated by FLT3‐ITD mutation status. Table S1. (A) Cohort Composition – Beat AML sample cohort (all). (B) Cohort Composition – Beat AML sample cohort (no FLT3‐ITD). (C) Cohort Composit [file MOL2-15-2285-s001.docx]

**­**

**Sex disparity in acute myeloid leukaemia with *FLT3* internal tandem duplication mutations: implications for prognosis**

Supplementary material

A


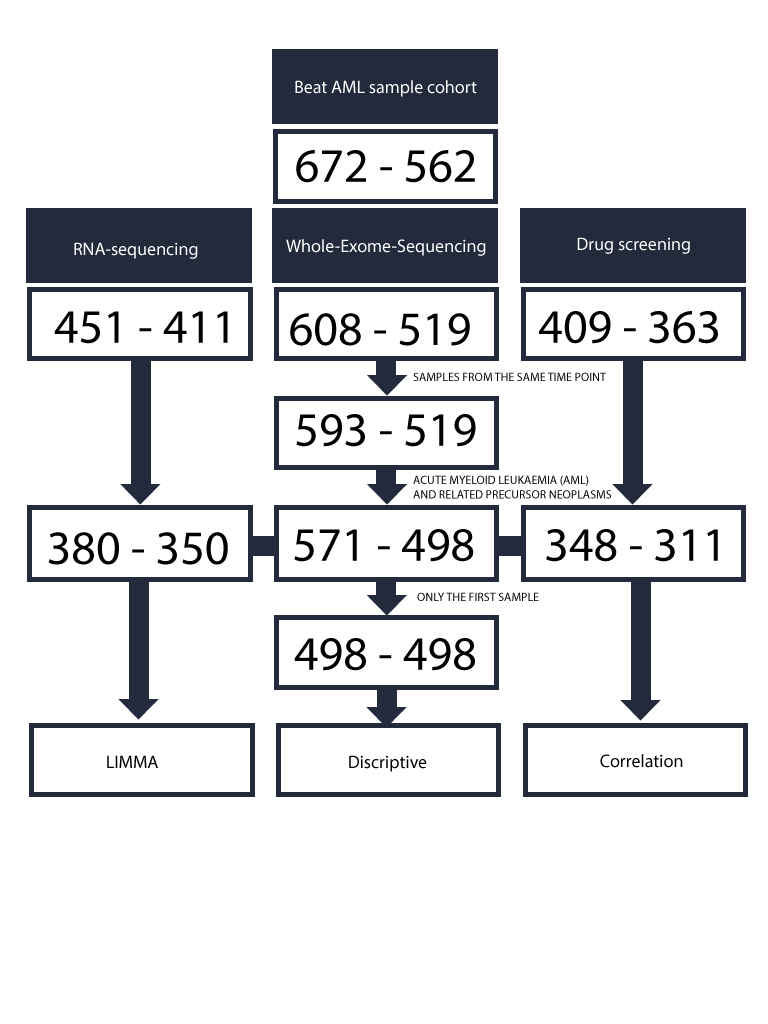


B

Supplementary figure 1: A. Overview of the Beat AML sample cohort selection flow resulting in the restricted sample collection presented in the paper. B. Distribution of the 498 samples included in the descriptive analysis in accordance with sample type (specimenGroups) as annotated in the original Beat AML material. The survival analysis was further restricted to the 333 samples annotated as “Initial Acute Leukemia Diagnosis”, excluding samples where vital status was not annotated as “unknown”, resulting in a total of 303 samples.

| Supplementary table 1A  Cohort composition  Beat AML sample cohort (All) | | | | | | | | | | | | | |  |
| --- | --- | --- | --- | --- | --- | --- | --- | --- | --- | --- | --- | --- | --- | --- |
| **Variable** | | **Female** | | **Median (Range)** | | **Male** | | **Median (Range)** | | **p-value** | | **Adj. P-value** | |  |
|  | |  | |  | |  | |  | |  | |  | |  |
| Age (Diagnosis) | | 222(222) | | 56.5 (1-83) | | 273(276) | | 63 (5-87) | | 3.53276E-05 | | 0.000388603 | |  |
| Age (Specimen acquisition) | | 222(222) | | 57.5 (2-85) | | 274(276) | | 63 (5-87) | | 5.71085E-05 | | 0.000418795 | |  |
|  | |  | |  | |  | |  | |  | |  | |  |
| De novo AML | | 117/222 (52.7%) | | | | 133/276 (48.19%) | | | | 0.322909025 | | 0.33634536 | | * |
| Transformed | | 25/222 (11.26%) | | | | 61/276 (22.1%) | | | | 0.001814697 | | 0.012702882 | |  |
| Prior MDS | | 14/222 (6.31%) | | | | 34/276 (12.32%) | | | | 0.031622179 | | 0.104757722 | |  |
| Prior MPN | | 9/222 (4.05%) | | | | 17/276 (6.16%) | | | | 0.318633666 | | 0.33634536 | |  |
| Prior MDS/MPN | | 2/222 (0.9%) | | | | 11/276 (3.99%) | | | | 0.044896166 | | 0.104757722 | |  |
| Relapse | | 16/222 (7.21%) | | | | 12/276 (4.35%) | | | | 0.176764033 | | 0.309337058 | |  |
| Prior non-myeloid malignancy | | 31/222 (13.96%) | | | | 30/276 (10.87%) | | | | 0.33634536 | | 0.33634536 | |  |
|  | |  | |  | |  | |  | |  | |  | |  |
| Blasts in BM | | 154(222) | | 68.5 (1-98) | | 192(276) | | 60 (0-97) | | 0.097887897 | | 0.307647676 | |  |
| Blasts in PB | | 152(222) | | 42.05 (0-99) | | 194(276) | | 39 (0-99.2) | | 0.942579173 | | 0.970513379 | |  |
| Monocytes in PB | | 156(222) | | 5.55 (0-89.8) | | 196(276) | | 5.2 (0-70) | | 0.970513379 | | 0.970513379 | |  |
| Lymphocytes in PB | | 140(222) | | 22.35 (0.8-94.3) | | 180(276) | | 20.25 (0-100) | | 0.847390644 | | 0.970513379 | |  |
| Immature Granulocytes in PB | | 71(222) | | 0.8 (0-44) | | 77(276) | | 0.8 (0-46) | | 0.616580651 | | 0.847798395 | |  |
| Neutrophils in PB | | 137(222) | | 11 (0-71.9) | | 180(276) | | 12.95 (0-84.7) | | 0.41303478 | | 0.75723043 | |  |
| Basophils in PB | | 124(222) | | 0 (0-7.6) | | 156(276) | | 0 (0-10.8) | | 0.355210273 | | 0.710420545 | |  |
| Eosinophils in PB | | 131(222) | | 0 (0-30) | | 170(276) | | 0 (0-24) | | 0.073693875 | | 0.270210875 | |  |
| Nucleated RBCs in PB | | 80(222) | | 0.2 (0-32.3) | | 97(276) | | 0.1 (0-50) | | 0.692001022 | | 0.895530734 | |  |
|  | |  | |  | |  | |  | |  | |  | |  |
| Hemoglobin | | 141(222) | | 8.5 (4.8-13.4) | | 180(276) | | 8.85 (3.7-16.1) | | 0.164290178 | | 0.401598214 | |  |
| WBC count | | 189(222) | | 19.67 (0.1-206.64) | | 239(276) | | 11.4 (0.1-427.46) | | 0.14940087 | | 0.401598214 | |  |
| Platelet count | | 158(222) | | 36 (5-426) | | 200(276) | | 46 (3-916) | | 0.021281064 | | 0.09363668 | |  |
| MCV | | 114(222) | | 93.55 (70.8-112.6) | | 142(276) | | 93.95 (77-132.4) | | 0.590890861 | | 0.847798395 | |  |
| Hematocrit | | 140(222) | | 25.85 (14.3-41.1) | | 178(276) | | 26.25 (11.1-48.8) | | 0.2194257 | | 0.482736539 | |  |
| LDH | | 92(222) | | 402.5 (125-3587) | | 112(276) | | 404 (88-16499) | | 0.809762306 | | 0.970513379 | |  |
| Albumin | | 133(222) | | 3.2 (1.8-4.6) | | 176(276) | | 3.4 (1.7-4.8) | | 0.01641737 | | 0.090295537 | |  |
| Total protein | | 132(222) | | 6.7 (4.6-8.7) | | 174(276) | | 6.6 (4.3-8.8) | | 0.537232481 | | 0.844222471 | |  |
| Creatinine | | 131(222) | | 0.73 (0.21-4.22) | | 167(276) | | 0.93 (0.44-2.67) | | 3.46901E-12 | | 7.63182E-11 | |  |
| ALT | | 135(222) | | 27 (8-393) | | 175(276) | | 26 (4-463) | | 0.481182286 | | 0.814308484 | |  |
| AST | | 135(222) | | 27 (8-350) | | 176(276) | | 26 (7-477) | | 0.921418664 | | 0.970513379 | |  |
|  | |  | |  | |  | |  | |  | |  | |  |
| Age (Diagnosis): Age at diagnosis (years), Age (Specimen acquisition): Age at time of specimen acquisition (years), Blasts in BM: % Blast in the bone marrow, Blasts in PB : % Blast in the peripheral blood, Monocytes in PB: % Monocytes in the peripheral blood, Lymphocytes in PB :% Lymphocytes in the peripheral blood, Immature Granulocytes in PB : % Immature Granulocytes in the peripheral blood, Neutrophils in PB: % Neutrophils in the peripheral blood, Basophils in PB: % Basophils in the peripheral blood, Eosinophils in PB: % Eosinophils in the peripheral blood, Nucleated RBCs in PB: % Nucleated RBCs in the peripheral blood, Hemoglobin: Hemoglobin levels (%), WBC count: White blood cell count expressed in international units (x10^9^ cells/litre), Platelet count: Number of platelets (x10^9^ cells/litre), MCV: Mean corpuscular volume (fL), Hematocrit: Hemocrit levels (%), LDH: Lactic acid dehydrogenase levels (Units/Litre), Albumin: Albumin levels in the peripheral blood (g/dL), Total protein: Total protein levels in the blood (g/dL), Creatinine: Creatinine levels in the peripheral blood (mg/dL), ALT: Alanine aminotransferase levels in the peripheral blood (units/litre), AST: Aspartate aminotransferase levels in the peripheral blood (units/litre), MDS: myelodyplastic syndrome, MPN: myeloproliferative neoplasm. *p-value adjustment performed separately. | | | | | | | | | | | | | |  |
| Supplementary table 1B  Cohort composition  Beat AML sample cohort (No FLT3-ITD) | | | | | | | | | | | | |  |  |
| **Variable** | **Female** | | **Median (Range)** | | **Male** | | **Median (Range)** | | **p-value** | | **Adj. P-value** | |  |  |
|  |  | |  | |  | |  | |  | |  | |  |  |
| Age (Diagnosis) | 160(160) | | 54.5 (1-83) | | 212(215) | | 63.5 (5-87) | | 0.00029567 | | 0.003252372 | |  |  |
| Age (Specimen acquisition) | 160(160) | | 55.5 (2-85) | | 213(215) | | 64 (5-87) | | 0.000511517 | | 0.003751127 | |  |  |
|  |  | |  | |  | |  | |  | |  | |  |  |
| Blasts in BM | 116(160) | | 62.5 (1-98) | | 146(215) | | 52.5 (0-97) | | 0.055673304 | | 0.30620317 | |  |  |
| Blasts in PB | 109(160) | | 29 (0-97) | | 151(215) | | 28 (0-99) | | 0.831199245 | | 0.963867449 | |  |  |
| Monocytes in PB | 117(160) | | 7.6 (0-89.8) | | 156(215) | | 6.4 (0-70) | | 0.946228951 | | 0.963867449 | |  |  |
| Lymphocytes in PB | 100(160) | | 28.95 (1-94.3) | | 143(215) | | 24 (0-100) | | 0.644225383 | | 0.963867449 | |  |  |
| Immature Granulocytes in PB | 52(160) | | 0.8 (0-44) | | 66(215) | | 0.7 (0-46) | | 0.678416642 | | 0.963867449 | |  |  |
| Neutrophils in PB | 99(160) | | 12.2 (0-71.9) | | 143(215) | | 16 (0-84) | | 0.569468402 | | 0.963715757 | |  |  |
| Basophils in PB | 90(160) | | 0 (0-7.6) | | 122(215) | | 0 (0-10.8) | | 0.336396806 | | 0.822303303 | |  |  |
| Eosinophils in PB | 95(160) | | 0 (0-30) | | 136(215) | | 0 (0-24) | | 0.285876864 | | 0.786161376 | |  |  |
| Nucleated RBCs in PB | 59(160) | | 0.1 (0-32.3) | | 83(215) | | 0 (0-43.4) | | 0.873656348 | | 0.963867449 | |  |  |
|  |  | |  | |  | |  | |  | |  | |  |  |
| Hemoglobin | 101(160) | | 8.8 (4.8-13.4) | | 143(215) | | 8.8 (4.6-16.1) | | 0.285791713 | | 0.786161376 | |  |  |
| WBC count | 136(160) | | 10.34 (0.1-206.64) | | 189(215) | | 8.21 (0.1-427.46) | | 0.560032168 | | 0.963715757 | |  |  |
| Platelet count | 113(160) | | 38 (5-426) | | 162(215) | | 43 (3-916) | | 0.133307612 | | 0.488794576 | |  |  |
| MCV | 81(160) | | 93.6 (79-112.6) | | 119(215) | | 93.5 (77-118.6) | | 0.943452242 | | 0.963867449 | |  |  |
| Hematocrit | 101(160) | | 26.1 (14.3-41.1) | | 143(215) | | 26 (14-48.8) | | 0.395894166 | | 0.849613299 | |  |  |
| LDH | 64(160) | | 391.5 (125-2008) | | 89(215) | | 398 (88-16499) | | 0.807142778 | | 0.963867449 | |  |  |
| Albumin | 93(160) | | 3.2 (1.8-4.6) | | 141(215) | | 3.4 (1.7-4.8) | | 0.070929648 | | 0.31209045 | |  |  |
| Total protein | 92(160) | | 6.7 (4.8-8.7) | | 139(215) | | 6.6 (4.3-8.7) | | 0.963867449 | | 0.963867449 | |  |  |
| Creatinine | 93(160) | | 0.7 (0.21-4.22) | | 135(215) | | 0.94 (0.44-2.67) | | 8.95627E-12 | | 1.97038E-10 | |  |  |
| ALT | 93(160) | | 26 (9-393) | | 140(215) | | 24.5 (4-463) | | 0.42480665 | | 0.849613299 | |  |  |
| AST | 93(160) | | 25 (8-350) | | 141(215) | | 25 (7-477) | | 0.947264745 | | 0.963867449 | |  |  |
|  |  | |  | |  | |  | |  | |  | |  |  |
| Age (Diagnosis): Age at diagnosis (years), Age (Specimen acquisition): Age at time of specimen acquisition (years), Blasts in BM: % Blast in the bone marrow, Blasts in PB : % Blast in the peripheral blood, Monocytes in PB: % Monocytes in the peripheral blood, Lymphocytes in PB :% Lymphocytes in the peripheral blood, Immature Granulocytes in PB : % Immature Granulocytes in the peripheral blood, Neutrophils in PB: % Neutrophils in the peripheral blood, Basophils in PB: % Basophils in the peripheral blood, Eosinophils in PB: % Eosinophils in the peripheral blood, Nucleated RBCs in PB: % Nucleated RBCs in the peripheral blood, Hemoglobin: Hemoglobin levels (%), WBC count: White blood cell count expressed in international units (x10^9^ cells/litre), Platelet count: Number of platelets (x10^9^ cells/litre), MCV: Mean corpuscular volume (fL), Hematocrit: Hemocrit levels (%), LDH: Lactic acid dehydrogenase levels (Units/Litre), Albumin: Albumin levels in the peripheral blood (g/dL), Total protein: Total protein levels in the blood (g/dL), Creatinine: Creatinine levels in the peripheral blood (mg/dL), ALT: Alanine aminotransferase levels in the peripheral blood (units/litre), AST: Aspartate aminotransferase levels in the peripheral blood (units/litre). | | | | | | | | | | | | |  |  |

| Supplementary table 1C  Cohort composition  Beat AML sample cohort (FLT3-ITD positive) | | | | | | |
| --- | --- | --- | --- | --- | --- | --- |
| **Variable** | **Female** | **Median (Range)** | **Male** | **Median (Range)** | **p-value** | **Adj. P-value** |
|  |  |  |  |  |  |  |
| Age (Diagnosis) | 62(62) | 59.5 (22-79) | 61(61) | 61 (10-85) | 0.073228252 | 0.343614467 |
| Age (Specimen acquisition) | 62(62) | 60 (22-79) | 61(61) | 61 (10-85) | 0.066202447 | 0.343614467 |
|  |  | |  | |  |  |
| De novo AML | 35/62 (56.45%) | | 35/61 (57.38%) | | 1 | 1 |
| Transformed | 6/62 (9.68%) | | 8/61 (13.11%) | | 0.58310021 | 0.889802447 |
| Prior MPN | 3/62 (4.84%) | | 1/61 (1.64%) | | 0.618826849 | 0.889802447 |
| Prior MDS | 2/62 (3.23%) | | 3/61 (4.92%) | | 0.679686428 | 0.889802447 |
| Prior MDS/MPN | 1/62 (1.61%) | | 4/61 (6.56%) | | 0.20722391 | 0.889802447 |
| Prior non-myeloid malignancy | 5/62 (8.06%) | | 6/61 (9.84%) | | 0.762687812 | 0.889802447 |
| Relapse | 7/62 (11.29%) | | 4/61 (6.56%) | | 0.529538995 | 0.889802447 |
|  |  |  |  |  |  |  |
| Blasts in BM | 38(62) | 81.5 (1.5-98) | 46(61) | 81 (9-96) | 0.906902426 | 0.948484637 |
| Blasts in PB | 43(62) | 66 (0-99) | 43(61) | 67 (0-99.2) | 0.574469457 | 0.838240469 |
| Monocytes in PB | 39(62) | 3.5 (0-85.2) | 40(61) | 2.3 (0-60.9) | 0.533572474 | 0.838240469 |
| Lymphocytes in PB | 40(62) | 14.4 (0.8-40) | 37(61) | 9.5 (0-69) | 0.350768802 | 0.804219472 |
| Immature Granulocytes in PB | 19(62) | 0.7 (0-28.8) | 11(61) | 0.8 (0-19.6) | 0.655621717 | 0.838240469 |
| Neutrophils in PB | 38(62) | 5.85 (0-69) | 37(61) | 8 (0-84.7) | 0.78268204 | 0.860950244 |
| Basophils in PB | 34(62) | 0 (0-2) | 34(61) | 0 (0-1.4) | 0.948484637 | 0.948484637 |
| Eosinophils in PB | 36(62) | 0 (0-1.9) | 34(61) | 0 (0-8.5) | 0.232662874 | 0.654188266 |
| Nucleated RBCs in PB | 21(62) | 0.3 (0-2.8) | 14(61) | 0.2 (0-50) | 0.464530281 | 0.804219472 |
|  |  |  |  |  |  |  |
| Hemoglobin | 40(62) | 8.2 (5.3-13.2) | 37(61) | 9.1 (3.7-14) | 0.475220597 | 0.804219472 |
| WBC count | 53(62) | 41.91 (0.8-190) | 50(61) | 31.55 (1.8-282.1) | 0.656026437 | 0.838240469 |
| Platelet count | 45(62) | 35 (5-299) | 38(61) | 49 (9-330) | 0.07026415 | 0.343614467 |
| MCV | 33(62) | 93.5 (70.8-108.3) | 23(61) | 96 (85.6-132.4) | 0.083229338 | 0.343614467 |
| Hematocrit | 39(62) | 24.6 (14.5-40) | 35(61) | 26.3 (11.1-42.1) | 0.448474831 | 0.804219472 |
| LDH | 28(62) | 438.5 (184-3587) | 23(61) | 497 (91-4559) | 0.686568649 | 0.838240469 |
| Albumin | 40(62) | 3.15 (1.9-4.2) | 35(61) | 3.3 (1.8-4.2) | 0.093713036 | 0.343614467 |
| Total protein | 40(62) | 6.5 (4.6-7.7) | 35(61) | 6.7 (5.1-8.8) | 0.237886642 | 0.654188266 |
| Creatinine | 38(62) | 0.78 (0.46-1.87) | 32(61) | 0.92 (0.46-1.85) | 0.048899883 | 0.343614467 |
| ALT | 42(62) | 27 (8-195) | 35(61) | 30 (12-391) | 0.72393495 | 0.838240469 |
| AST | 42(62) | 30.5 (9-90) | 35(61) | 30 (15-343) | 0.43661279 | 0.804219472 |
|  |  |  |  |  |  |  |
| Age (Diagnosis): Age at diagnosis (years), Age (Specimen acquisition): Age at time of specimen acquisition (years), Blasts in BM: % Blast in the bone marrow, Blasts in PB : % Blast in the peripheral blood, Monocytes in PB: % Monocytes in the peripheral blood, Lymphocytes in PB :% Lymphocytes in the peripheral blood, Immature Granulocytes in PB : % Immature Granulocytes in the peripheral blood, Neutrophils in PB: % Neutrophils in the peripheral blood, Basophils in PB: % Basophils in the peripheral blood, Eosinophils in PB: % Eosinophils in the peripheral blood, Nucleated RBCs in PB: % Nucleated RBCs in the peripheral blood, Hemoglobin: Hemoglobin levels (%), WBC count: White blood cell count expressed in international units (x10^9^ cells/litre), Platelet count: Number of platelets (x10^9^ cells/litre), MCV: Mean corpuscular volume (fL), Hematocrit: Hemocrit levels (%), LDH: Lactic acid dehydrogenase levels (Units/Litre), Albumin: Albumin levels in the peripheral blood (g/dL), Total protein: Total protein levels in the blood (g/dL), Creatinine: Creatinine levels in the peripheral blood (mg/dL), ALT: Alanine aminotransferase levels in the peripheral blood (units/litre), AST: Aspartate aminotransferase levels in the peripheral blood (units/litre) MDS: myelodyplastic syndrome, MPN: myeloproliferative neoplasm. | | | | | | |

| Supplementary table 1D  Cohort composition  Beat AML sample cohort (All) | | | | | | |
| --- | --- | --- | --- | --- | --- | --- |
| **Variable** | **FLT3-ITD** | **Median (Range)** | **No FLT3-ITD** | **Median (Range)** | **p-value** | **Adj. P-value** |
|  |  |  |  |  |  |  |
| Age (Diagnosis) | 123(123) | 61 (10-85) | 372(375) | 61.5 (1-87) | 0.280410939 | 0.420349637 |
| Age (Specimen acquisition) | 123(123) | 61 (10-85) | 373(375) | 62 (2-87) | 0.286602025 | 0.420349637 |
|  |  |  |  |  |  |  |
| Blasts in BM | 84(123) | 81 (1.5-98) | 262(375) | 55 (0-98) | 2.72787E-08 | 1.50033E-07 |
| Blasts in PB | 86(123) | 66.15 (0-99.2) | 260(375) | 28.75 (0-99) | 7.75056E-11 | 8.52562E-10 |
| Monocytes in PB | 79(123) | 3 (0-85.2) | 273(375) | 7 (0-89.8) | 0.001313853 | 0.005780952 |
| Lymphocytes in PB | 77(123) | 11.4 (0-69) | 243(375) | 26.3 (0-100) | 3.75847E-09 | 2.75621E-08 |
| Immature Granulocytes in PB | 30(123) | 0.75 (0-28.8) | 118(375) | 0.8 (0-46) | 0.823448997 | 0.862660854 |
| Neutrophils in PB | 75(123) | 8 (0-84.7) | 242(375) | 14.1 (0-84) | 0.004551498 | 0.014304709 |
| Basophils in PB | 68(123) | 0 (0-2) | 212(375) | 0 (0-10.8) | 0.135705102 | 0.271410205 |
| Eosinophils in PB | 70(123) | 0 (0-8.5) | 231(375) | 0 (0-30) | 0.003454186 | 0.01266535 |
| Nucleated RBCs in PB | 35(123) | 0.2 (0-50) | 142(375) | 0.1 (0-43.4) | 0.097653863 | 0.214838498 |
|  |  |  |  |  |  |  |
| Hemoglobin | 77(123) | 8.6 (3.7-14) | 244(375) | 8.8 (4.6-16.1) | 0.477718843 | 0.583878586 |
| WBC count | 103(123) | 34 (0.8-282.1) | 325(375) | 8.8 (0.1-427.46) | 4.69475E-11 | 8.52562E-10 |
| Platelet count | 83(123) | 42 (5-330) | 275(375) | 42 (3-916) | 0.390860284 | 0.505819191 |
| MCV | 56(123) | 95.4 (70.8-132.4) | 200(375) | 93.5 (77-118.6) | 0.224398322 | 0.379751006 |
| Hematocrit | 74(123) | 25.4 (11.1-42.1) | 244(375) | 26.1 (14-48.8) | 0.333491518 | 0.458550837 |
| LDH | 51(123) | 451 (91-4559) | 153(375) | 394 (88-16499) | 0.054168162 | 0.132411063 |
| Albumin | 75(123) | 3.3 (1.8-4.2) | 234(375) | 3.3 (1.7-4.8) | 0.870647832 | 0.870647832 |
| Total protein | 75(123) | 6.5 (4.6-8.8) | 231(375) | 6.7 (4.3-8.7) | 0.682569855 | 0.75082684 |
| Creatinine | 70(123) | 0.83 (0.46-1.87) | 228(375) | 0.86 (0.21-4.22) | 0.650128033 | 0.75082684 |
| ALT | 77(123) | 27 (8-391) | 233(375) | 25 (4-463) | 0.166546486 | 0.305335224 |
| AST | 77(123) | 30 (9-343) | 234(375) | 25 (7-477) | 0.016228835 | 0.044629295 |
|  |  |  |  |  |  |  |
| Age (Diagnosis): Age at diagnosis (years), Age (Specimen acquisition): Age at time of specimen acquisition (years), Blasts in BM: % Blast in the bone marrow, Blasts in PB : % Blast in the peripheral blood, Monocytes in PB: % Monocytes in the peripheral blood, Lymphocytes in PB :% Lymphocytes in the peripheral blood, Immature Granulocytes in PB : % Immature Granulocytes in the peripheral blood, Neutrophils in PB: % Neutrophils in the peripheral blood, Basophils in PB: % Basophils in the peripheral blood, Eosinophils in PB: % Eosinophils in the peripheral blood, Nucleated RBCs in PB: % Nucleated RBCs in the peripheral blood, Hemoglobin: Hemoglobin levels (%), WBC count: White blood cell count expressed in international units (x10^9^ cells/litre), Platelet count: Number of platelets (x10^9^ cells/litre), MCV: Mean corpuscular volume (fL), Hematocrit: Hemocrit levels (%), LDH: Lactic acid dehydrogenase levels (Units/Litre), Albumin: Albumin levels in the peripheral blood (g/dL), Total protein: Total protein levels in the blood (g/dL), Creatinine: Creatinine levels in the peripheral blood (mg/dL), ALT: Alanine aminotransferase levels in the peripheral blood (units/litre), AST: Aspartate aminotransferase levels in the peripheral blood (units/litre) | | | | | | |
|  |  |  |  |  |  |  |

| Supplementary table 2  Cohort composition  HOVON 1 - All – Sex | | | | | | |  |
| --- | --- | --- | --- | --- | --- | --- | --- |
| **Variable** | **Female** | **Median (Range)** | **Male** | **Median (Range)** | **p-value** | **Adj. p-value** |  |
|  |  |  |  |  |  |  |  |
| Age | 215 (215) | 46 (16-77) | 217 (217) | 46 (15-75) | 0.352093502 | 0.704187005 | * |
| BM blasts | 211 (215) | 66 (0-97) | 215 (217) | 70 (10-98) | 0.962955957 | 0.990591615 |  |
| PLT | 215 (215) | 60 (3-998) | 216 (217) | 53 (5-742) | 0.183032216 | 0.704187005 |  |
| WBC | 215 (215) | 25 (0.6-278) | 215 (217) | 34.4 (0.6-510) | 0.708632879 | 0.990591615 |  |
|  |  |  |  |  |  |  |  |
| Allo HSCT | 62/215 (28.84%) | | 65/217 (29.95%) | | 0.833094282 | 1 | * |
| Auto HSCT | 23/215 (10.7%) | | 37/217 (17.05%) | | 0.070061953 | 0.425123 |  |
| No HSCT | 130/215 (60.47%) | | 115/217 (53%) | | 0.121463714 | 0.425123 |  |
| Prior chrt | 3/215 (1.4%) | | 6/217 (2.76%) | | 0.503239537 | 1 |  |
| Prior MDS | 10/215 (4.65%) | | 10/217 (4.61%) | | 1 | 1 |  |
|  |  |  |  |  |  |  |  |
|  |  |  |  |  |  |  |  |
| HOVON 1 - No FLT3-ITD - Sex | | | | | | |  |
| **Variable** | **Female** | **Median (Range)** | **Male** | **Median (Range)** | **p-value** | **Adj. p-value** |  |
|  |  |  |  |  |  |  |  |
| Age | 148 (148) | 46 (16-74) | 167 (167) | 45 (15-75) | 0.212726149 | 0.283634865 | * |
| BM blasts | 145 (148) | 63 (0-97) | 167 (167) | 66 (10-98) | 0.562662009 | 0.562662009 |  |
| PLT | 148 (148) | 60 (3-998) | 166 (167) | 49.5 (5-742) | 0.081695257 | 0.216691723 |  |
| WBC | 148 (148) | 17.65 (0.6-269) | 165 (167) | 25.1 (0.6-244) | 0.108345862 | 0.216691723 |  |
|  |  |  |  |  |  |  |  |
| Allo HSCT | 45/148 (30.41%) | | 53/167 (31.74%) | | 0.808637509 | 1 | * |
| Auto HSCT | 17/148 (11.49%) | | 28/167 (16.77%) | | 0.199627978 | 0.90326173 |  |
| No HSCT | 86/148 (58.11%) | | 86/167 (51.5%) | | 0.25807478 | 0.90326173 |  |
| Prior chrt | 3/148 (2.03%) | | 6/167 (3.59%) | | 0.508806747 | 1 |  |
| Prior MDS | 7/148 (4.73%) | | 10/167 (5.99%) | | 0.803629313 | 1 |  |
|  |  |  |  |  |  |  |  |
|  |  |  |  |  |  |  |  |
| HOVON 1 - FLT3-ITD - Sex | | | | | | |  |
| **Variable** | **Female** | **Median (Range)** | **Male** | **Median (Range)** | **p-value** | **Adj. p-value** |  |
|  |  |  |  |  |  |  |  |
| Age | 67 (67) | 45 (18-77) | 50 (50) | 47.5 (19-71) | 0.639325204 | 0.84971713 | * |
| BM blasts | 66 (67) | 82 (27-95) | 48 (50) | 79.5 (14-98) | 0.84971713 | 0.84971713 |  |
| PLT | 67 (67) | 60 (10-603) | 50 (50) | 75 (21-687) | 0.39000674 | 0.780013479 |  |
| WBC | 67 (67) | 69 (2.2-278) | 50 (50) | 46 (2.7-510) | 0.339089739 | 0.780013479 |  |
|  |  |  |  |  |  |  |  |
| Allo HSCT | 17/67 (25.37%) | | 12/50 (24%) | | 1 | 1 | * |
| Auto HSCT | 6/67 (8.96%) | | 9/50 (18%) | | 0.170506535 | 0.90826702 |  |
| No HSCT | 44/67 (65.67%) | | 29/50 (58%) | | 0.443361055 | 1 |  |
| Prior chrt | 0/67 (0%) | | 0/50 (0%) | | 1 | 1 |  |
| Prior MDS | 3/67 (4.48%) | | 0/50 (0%) | | 0.259504863 | 0.90826702 |  |
|  |  |  |  |  |  |  |  |
|  |  |  |  |  |  |  |  |
| HOVON 1 -All - FLT3-ITD status | | | | | | |  |
| **Variable** | **FLT3-ITD** | **Median (Range)** | **no FLT3-ITD** | **Median (Range)** | **p-value** | **Adj. p-value** |  |
|  |  |  |  |  |  |  |  |
| Age | 117 (117) | 47 (18-77) | 315 (315) | 45 (15-75) | 0.328283736 | 0.328283736 |  |
| BM blasts | 114 (117) | 81 (14-98) | 312 (315) | 64.5 (0-98) | 2.03433E-05 | 4.06867E-05 |  |
| PLT | 117 (117) | 64 (10-687) | 314 (315) | 54 (3-998) | 0.056070055 | 0.074760073 |  |
| WBC | 117 (117) | 57.8 (2.2-510) | 313 (315) | 22.1 (0.6-269) | 1.46971E-08 | 5.87885E-08 |  |
|  |  |  |  |  |  |  |  |
| BM blasts: Bone marrow blast %, PLT: platelet count (x10^9^/L), WBC: white blood cell count × (x10^9^/L)  Allo HSCT: allogeneic hematopoietic stem cell transplantation. Auto HSCT: autologous hematopoietic stem cell transplantation. No HSCT: no hematopoietic stem cell transplantation. Prior chrt: prior chemotherapy. Prior MDS: prior myelodysplastic syndrome. *p-value adjustment performed separately. | | | | | | |  |
| Supplementary table 3  Cohort composition  HOVON 2 - All - Sex | | | | | | |  |
| **Variable** | **Female** | **Median (Range)** | **Male** | **Median (Range)** | **p-value** | **Adj. p-value** |  |
|  |  |  |  |  |  |  |  |
| Age | 273 (273) | 53 (19-65) | 352 (352) | 55 (18-65) | 0.12347199 | 0.271665644 | * |
| PLT | 272 (273) | 61 (6-550) | 350 (352) | 55 (4-889) | 0.18111043 | 0.271665644 |  |
| WBC | 272 (273) | 10.8 (0.4-255.4) | 351 (352) | 8.7 (0.5-268.7) | 0.471857572 | 0.471857572 |  |
|  |  |  |  |  |  |  |  |
| Allo HSCT | 135/272 (49.63%) | | 178/352 (50.57%) | | 0.871754624 | 0.871754624 | * |
| Auto HSCT | 24/272 (8.82%) | | 37/352 (10.51%) | | 0.500262924 | 0.607902962 |  |
| No HSCT | 113/272 (41.54%) | | 137/352 (38.92%) | | 0.511175985 | 0.607902962 |  |
| Prior chrt | 8/265 (3.02%) | | 14/344 (4.07%) | | 0.521059682 | 0.607902962 |  |
| Prior dis | 28/272 (10.29%) | | 27/352 (7.67%) | | 0.258257714 | 0.602601332 |  |
| Prior ins | 0/194 (0%) | | 3/242 (1.24%) | | 0.257386903 | 0.602601332 |  |
| Prior MDS | 19/271 (7.01%) | | 11/351 (3.13%) | | 0.03634444 | 0.254411079 |  |
|  |  |  |  |  |  |  |  |
|  |  |  |  |  |  |  |  |
| HOVON 2 - No FLT3-ITD - Sex | | | | | | |  |
| **Variable** | **Female** | **Median (Range)** | **Male** | **Median (Range)** | **p-value** | **Adj. p-value** |  |
|  |  |  |  |  |  |  |  |
| Age | 199 (199) | 52 (19-65) | 280 (280) | 56 (18-65) | 0.01594678 | 0.04784034 | * |
| PLT | 198 (199) | 61.5 (6-550) | 279 (280) | 54 (7-889) | 0.395634953 | 0.59345243 |  |
| WBC | 198 (199) | 6.55 (0.4-255.4) | 279 (280) | 6.1 (0.5-228.7) | 0.911428382 | 0.911428382 |  |
|  |  |  |  |  |  |  |  |
| Allo HSCT | 85/198 (42.93%) | | 134/280 (47.86%) | | 0.306064522 | 0.42849033 | * |
| Auto HSCT | 17/198 (8.59%) | | 29/280 (10.36%) | | 0.637085579 | 0.743266508 |  |
| No HSCT | 96/198 (48.48%) | | 117/280 (41.79%) | | 0.161469615 | 0.382375325 |  |
| Prior chrt | 6/193 (3.11%) | | 10/274 (3.65%) | | 0.80257171 | 0.80257171 |  |
| Prior dis | 24/198 (12.12%) | | 23/280 (8.21%) | | 0.163875139 | 0.382375325 |  |
| Prior ins | 0/147 (0%) | | 3/192 (1.56%) | | 0.261035765 | 0.42849033 |  |
| Prior MDS | 14/198 (7.07%) | | 9/279 (3.23%) | | 0.080553512 | 0.382375325 |  |
|  |  |  |  |  |  |  |  |
|  |  |  |  |  |  |  |  |
| HOVON 2 - FLT3-ITD - Sex | | | | | | |  |
| **Variable** | **Female** | **Median (Range)** | **Male** | **Median (Range)** | **p-value** | **Adj. p-value** |  |
|  |  |  |  |  |  |  |  |
| Age | 74 (74) | 53 (19-65) | 72 (72) | 48.5 (18-65) | 0.130016327 | 0.390048982 | * |
| PLT | 74 (74) | 60 (9-310) | 71 (72) | 62 (4-574) | 0.331493865 | 0.497240797 |  |
| WBC | 74 (74) | 43 (0.8-191.5) | 72 (72) | 35.3 (0.7-268.7) | 1 | 1 |  |
|  |  |  |  |  |  |  |  |
| Allo HSCT | 50/74 (67.57%) | | 44/72 (61.11%) | | 0.49004791 | 0.997699496 | * |
| Auto HSCT | 7/74 (9.46%) | | 8/72 (11.11%) | | 0.790676461 | 1 |  |
| No HSCT | 17/74 (22.97%) | | 20/72 (27.78%) | | 0.570113998 | 0.997699496 |  |
| Prior chrt | 2/72 (2.78%) | | 4/70 (5.71%) | | 0.438057878 | 0.997699496 |  |
| Prior dis | 4/74 (5.41%) | | 4/72 (5.56%) | | 1 | 1 |  |
| Prior ins | 0/47 (0%) | | 0/50 (0%) | | 1 | 1 |  |
| Prior MDS | 5/73 (6.85%) | | 2/72 (2.78%) | | 0.441570169 | 0.997699496 |  |
|  |  |  |  |  |  |  |  |
|  |  |  |  |  |  |  |  |
| HOVON 2 -All - FLT3-ITD status | | | | | | |  |
| **Variable** | **FLT3-ITD** | **Median (Range)** | **no FLT3-ITD** | **Median (Range)** | **p-value** | **Adj. p-value** |  |
|  |  |  |  |  |  |  |  |
| WBC | 146 (146) | 37.7 (0.7-268.7) | 477 (479) | 6.2 (0.4-255.4) | 3.31691E-21 | 9.95074E-21 | * |
| Age | 146 (146) | 50 (18-65) | 479 (479) | 55 (18-65) | 0.041655503 | 0.062483254 |  |
| PLT | 145 (146) | 60 (4-574) | 477 (479) | 58 (6-889) | 0.607974164 | 0.607974164 |  |
|  |  |  |  |  |  |  |  |
| PLT: platelet count (x10^9^/L), WBC: white blood cell count × (x10^9^/L)  Allo HSCT: allogeneic hematopoietic stem cell transplantation. Auto HSCT: autologous hematopoietic stem cell transplantation. No HSCT: no hematopoietic stem cell transplantation. Prior chrt: prior chemotherapy. Prior MDS: prior myelodysplastic syndrome. Prior ins: Prior exposure to insecticide. Prior dis: Prior hematological or oncological disease. *p-value adjustment performed separately. | | | | | | |  |


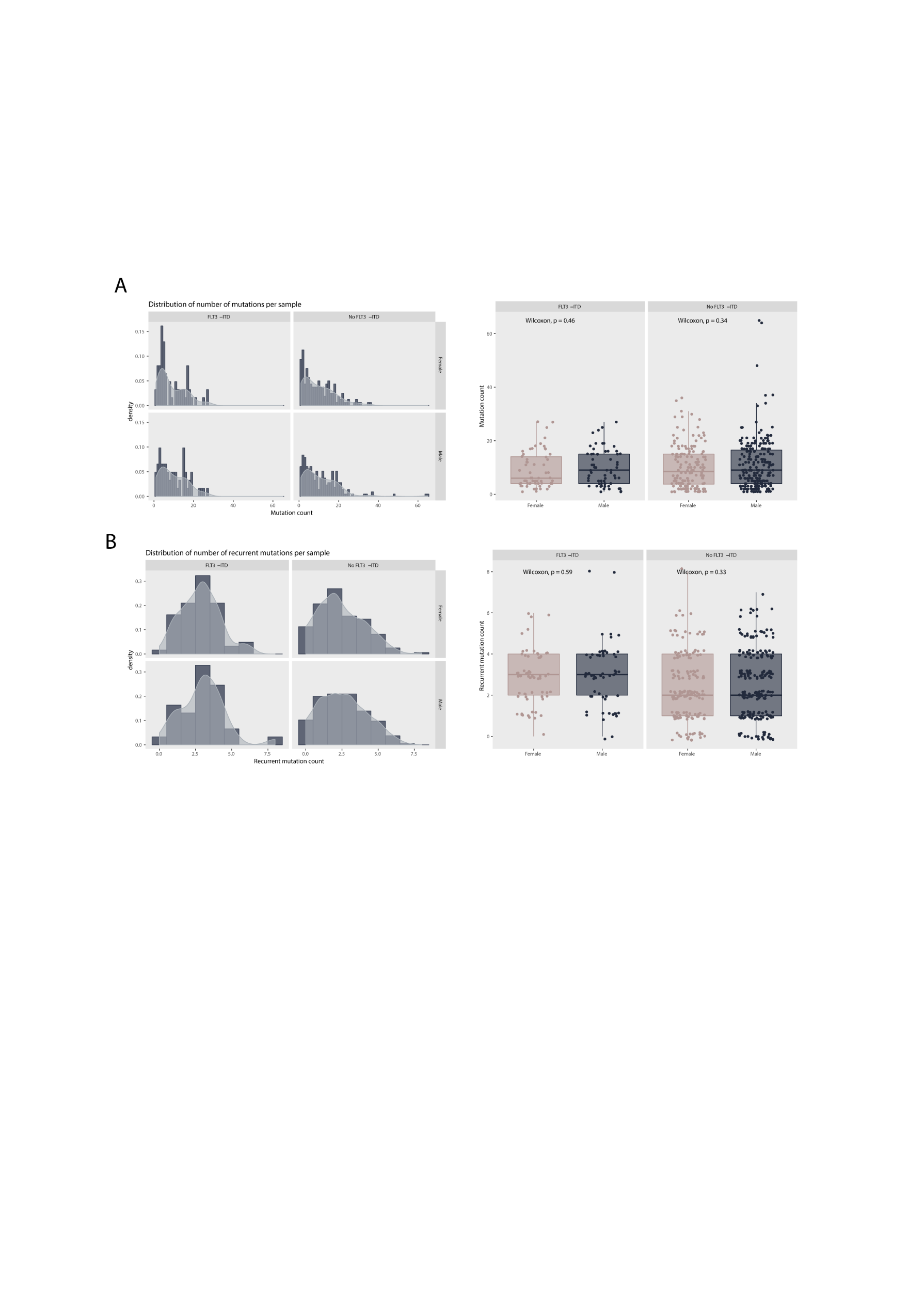


Supplementary figure 2: A. Distribution of exome sequencing somatic variant calls in the sample selection of Beat AML sample cohort (n=498) in accordance with sex and FLT3-ITD mutation status. B. Distribution of exome sequencing somatic variant calls that were within genes where variants were called a minimum of 10 times in the sample selection of Beat AML sample cohort (n=498) in accordance with sex and FLT3-ITD mutation status.


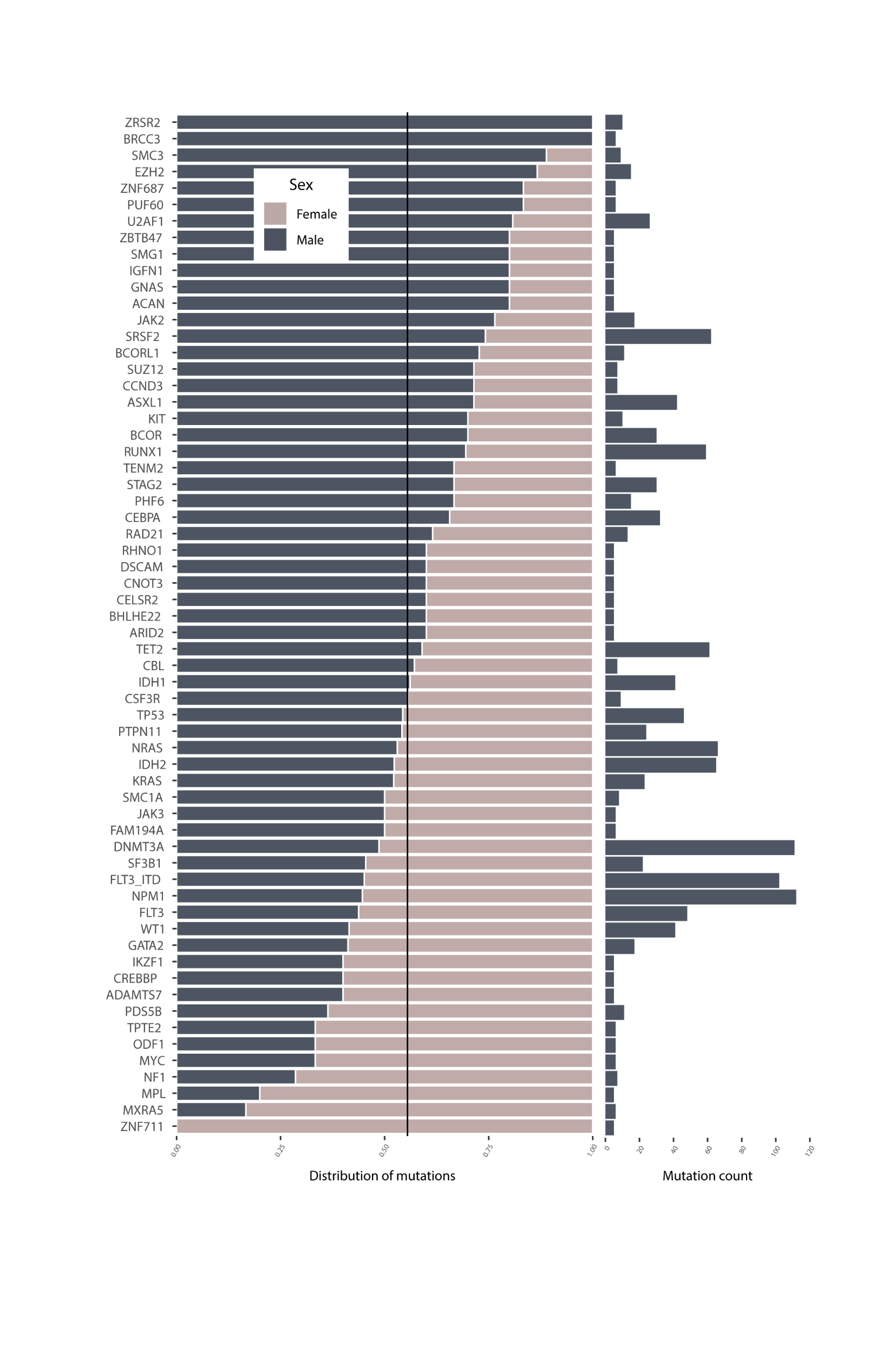


Supplementary figure 3: Sex-specific distribution of somatic mutations in the Beat AML sample cohort. Genes identified as mutated in a minimum of five times are presented. The vertical line represents the expected distribution assuming equal mutation frequency. The right panel shows the total number of mutated samples.

| Supplementary table 4A  Somatic mutations in the Beat AML sample cohort (All) | | | | |
| --- | --- | --- | --- | --- |
| **Gene** | **Female** | **Male** | **p-value** | **Adj. p-value** |
| ACAN | 1/222 (0.45%) | 4/276 (1.45%) | 0.387206 | 0.743353 |
| ADAMTS7 | 3/222 (1.35%) | 2/276 (0.72%) | 0.660110 | 0.936436 |
| ARID2 | 2/222 (0.9%) | 3/276 (1.09%) | 1.000000 | 1.000000 |
| ASXL1 | 12/222 (5.41%) | 30/276 (10.87%) | 0.034511 | 0.242873 |
| BCOR | 9/222 (4.05%) | 21/276 (7.61%) | 0.128880 | 0.491357 |
| BCORL1 | 3/222 (1.35%) | 8/276 (2.9%) | 0.359977 | 0.743353 |
| BHLHE22 | 2/222 (0.9%) | 3/276 (1.09%) | 1.000000 | 1.000000 |
| BRCC3 | 0/222 (0%) | 6/276 (2.17%) | 0.035834 | 0.242873 |
| CBL | 3/222 (1.35%) | 4/276 (1.45%) | 1.000000 | 1.000000 |
| CCND3 | 2/222 (0.9%) | 5/276 (1.81%) | 0.469138 | 0.773443 |
| CEBPA | 11/222 (4.95%) | 21/276 (7.61%) | 0.272037 | 0.721491 |
| CELSR2 | 2/222 (0.9%) | 3/276 (1.09%) | 1.000000 | 1.000000 |
| CNOT3 | 2/222 (0.9%) | 3/276 (1.09%) | 1.000000 | 1.000000 |
| CREBBP | 3/222 (1.35%) | 2/276 (0.72%) | 0.660110 | 0.936436 |
| CSF3R | 4/222 (1.8%) | 5/276 (1.81%) | 1.000000 | 1.000000 |
| DNMT3A | 57/222 (25.68%) | 54/276 (19.57%) | 0.105827 | 0.430362 |
| DSCAM | 2/222 (0.9%) | 3/276 (1.09%) | 1.000000 | 1.000000 |
| EZH2 | 2/222 (0.9%) | 13/276 (4.71%) | 0.015788 | 0.174521 |
| FAM194A | 3/222 (1.35%) | 3/276 (1.09%) | 1.000000 | 1.000000 |
| FLT3 | 82/222 (36.94%) | 79/276 (28.62%) | 0.054084 | 0.299919 |
| GATA2 | 10/222 (4.5%) | 7/276 (2.54%) | 0.320919 | 0.743353 |
| GNAS | 1/222 (0.45%) | 4/276 (1.45%) | 0.387206 | 0.743353 |
| IDH1 | 18/222 (8.11%) | 23/276 (8.33%) | 1.000000 | 1.000000 |
| IDH2 | 31/222 (13.96%) | 34/276 (12.32%) | 0.595278 | 0.907799 |
| IGFN1 | 1/222 (0.45%) | 4/276 (1.45%) | 0.387206 | 0.743353 |
| IKZF1 | 3/222 (1.35%) | 2/276 (0.72%) | 0.660110 | 0.936436 |
| JAK2 | 4/222 (1.8%) | 13/276 (4.71%) | 0.086235 | 0.404639 |
| JAK3 | 3/222 (1.35%) | 3/276 (1.09%) | 1.000000 | 1.000000 |
| KIT | 3/222 (1.35%) | 7/276 (2.54%) | 0.523395 | 0.840187 |
| KRAS | 11/222 (4.95%) | 12/276 (4.35%) | 0.831107 | 1.000000 |
| MPL | 4/222 (1.8%) | 1/276 (0.36%) | 0.177274 | 0.636102 |
| MXRA5 | 5/222 (2.25%) | 1/276 (0.36%) | 0.093495 | 0.407370 |
| MYC | 4/222 (1.8%) | 2/276 (0.72%) | 0.414328 | 0.743353 |
| NF1 | 5/222 (2.25%) | 2/276 (0.72%) | 0.250625 | 0.710151 |
| NPM1 | 62/222 (27.93%) | 50/276 (18.12%) | 0.009735 | 0.148454 |
| NRAS | 31/222 (13.96%) | 35/276 (12.68%) | 0.691766 | 0.944348 |
| ODF1 | 4/222 (1.8%) | 2/276 (0.72%) | 0.414328 | 0.743353 |
| PDS5B | 7/222 (3.15%) | 4/276 (1.45%) | 0.230485 | 0.709077 |
| PHF6 | 5/222 (2.25%) | 10/276 (3.62%) | 0.437722 | 0.762887 |
| PTPN11 | 11/222 (4.95%) | 13/276 (4.71%) | 1.000000 | 1.000000 |
| PUF60 | 1/222 (0.45%) | 5/276 (1.81%) | 0.232484 | 0.709077 |
| RAD21 | 5/222 (2.25%) | 8/276 (2.9%) | 0.780861 | 1.000000 |
| RHNO1 | 2/222 (0.9%) | 3/276 (1.09%) | 1.000000 | 1.000000 |
| RUNX1 | 18/222 (8.11%) | 41/276 (14.86%) | 0.025130 | 0.218990 |
| SF3B1 | 12/222 (5.41%) | 10/276 (3.62%) | 0.383917 | 0.743353 |
| SMC1A | 4/222 (1.8%) | 4/276 (1.45%) | 1.000000 | 1.000000 |
| SMC3 | 1/222 (0.45%) | 8/276 (2.9%) | 0.047315 | 0.288620 |
| SMG1 | 1/222 (0.45%) | 4/276 (1.45%) | 0.387206 | 0.743353 |
| SRSF2 | 16/222 (7.21%) | 46/276 (16.67%) | 0.001564 | 0.085866 |
| STAG2 | 10/222 (4.5%) | 20/276 (7.25%) | 0.256120 | 0.710151 |
| SUZ12 | 2/222 (0.9%) | 5/276 (1.81%) | 0.469138 | 0.773443 |
| TENM2 | 2/222 (0.9%) | 4/276 (1.45%) | 0.696650 | 0.944348 |
| TET2 | 25/222 (11.26%) | 36/276 (13.04%) | 0.584292 | 0.907799 |
| TP53 | 21/222 (9.46%) | 25/276 (9.06%) | 0.877710 | 1.000000 |
| TPTE2 | 4/222 (1.8%) | 2/276 (0.72%) | 0.414328 | 0.743353 |
| U2AF1 | 5/222 (2.25%) | 21/276 (7.61%) | 0.007884 | 0.148454 |
| WT1 | 24/222 (10.81%) | 17/276 (6.16%) | 0.071313 | 0.362506 |
| ZBTB47 | 1/222 (0.45%) | 4/276 (1.45%) | 0.387206 | 0.743353 |
| ZNF687 | 1/222 (0.45%) | 5/276 (1.81%) | 0.232484 | 0.709077 |
| ZNF711 | 5/222 (2.25%) | 0/276 (0%) | 0.017166 | 0.174521 |
| ZRSR2 | 0/222 (0%) | 10/276 (3.62%) | 0.002815 | 0.085866 |
|  |  |  |  |  |

| Supplementary table 4B  Somatic mutations in the Beat AML sample cohort (FLT3-ITD positive) | | | | |
| --- | --- | --- | --- | --- |
| **Gene** | **Female** | **Male** | **p-value** | **Adj. p-value** |
| ACAN | 0/62 (0%) | 1/61 (1.64%) | 0.495935 | 0.945376 |
| ADAMTS7 | 1/62 (1.61%) | 1/61 (1.64%) | 1.000000 | 1.000000 |
| ARID2 | 1/62 (1.61%) | 0/61 (0%) | 1.000000 | 1.000000 |
| ASXL1 | 2/62 (3.23%) | 5/61 (8.2%) | 0.272724 | 0.945376 |
| BCOR | 0/62 (0%) | 4/61 (6.56%) | 0.057482 | 0.945376 |
| BCORL1 | 0/62 (0%) | 1/61 (1.64%) | 0.495935 | 0.945376 |
| BHLHE22 | 1/62 (1.61%) | 0/61 (0%) | 1.000000 | 1.000000 |
| BRCC3 | 0/62 (0%) | 1/61 (1.64%) | 0.495935 | 0.945376 |
| CBL | 0/62 (0%) | 0/61 (0%) | 1.000000 | 1.000000 |
| CCND3 | 0/62 (0%) | 4/61 (6.56%) | 0.057482 | 0.945376 |
| CEBPA | 2/62 (3.23%) | 0/61 (0%) | 0.495935 | 0.945376 |
| CELSR2 | 0/62 (0%) | 2/61 (3.28%) | 0.243902 | 0.945376 |
| CNOT3 | 2/62 (3.23%) | 1/61 (1.64%) | 1.000000 | 1.000000 |
| CREBBP | 1/62 (1.61%) | 0/61 (0%) | 1.000000 | 1.000000 |
| CSF3R | 0/62 (0%) | 1/61 (1.64%) | 0.495935 | 0.945376 |
| DNMT3A | 22/62 (35.48%) | 18/61 (29.51%) | 0.564702 | 1.000000 |
| DSCAM | 1/62 (1.61%) | 0/61 (0%) | 1.000000 | 1.000000 |
| EZH2 | 0/62 (0%) | 1/61 (1.64%) | 0.495935 | 0.945376 |
| FAM194A | 0/62 (0%) | 0/61 (0%) | 1.000000 | 1.000000 |
| FLT3 | 62/62 (100%) | 61/61 (100%) | 1.000000 | 1.000000 |
| GATA2 | 1/62 (1.61%) | 0/61 (0%) | 1.000000 | 1.000000 |
| GNAS | 0/62 (0%) | 1/61 (1.64%) | 0.495935 | 0.945376 |
| IDH1 | 5/62 (8.06%) | 4/61 (6.56%) | 1.000000 | 1.000000 |
| IDH2 | 6/62 (9.68%) | 7/61 (11.48%) | 0.777653 | 1.000000 |
| IGFN1 | 0/62 (0%) | 3/61 (4.92%) | 0.118928 | 0.945376 |
| IKZF1 | 0/62 (0%) | 1/61 (1.64%) | 0.495935 | 0.945376 |
| JAK2 | 0/62 (0%) | 2/61 (3.28%) | 0.243902 | 0.945376 |
| JAK3 | 0/62 (0%) | 0/61 (0%) | 1.000000 | 1.000000 |
| KIT | 0/62 (0%) | 0/61 (0%) | 1.000000 | 1.000000 |
| KRAS | 1/62 (1.61%) | 3/61 (4.92%) | 0.364711 | 0.945376 |
| MPL | 0/62 (0%) | 0/61 (0%) | 1.000000 | 1.000000 |
| MXRA5 | 0/62 (0%) | 0/61 (0%) | 1.000000 | 1.000000 |
| MYC | 1/62 (1.61%) | 0/61 (0%) | 1.000000 | 1.000000 |
| NF1 | 1/62 (1.61%) | 0/61 (0%) | 1.000000 | 1.000000 |
| NPM1 | 29/62 (46.77%) | 20/61 (32.79%) | 0.141343 | 0.945376 |
| NRAS | 0/62 (0%) | 1/61 (1.64%) | 0.495935 | 0.945376 |
| ODF1 | 3/62 (4.84%) | 0/61 (0%) | 0.243902 | 0.945376 |
| PDS5B | 2/62 (3.23%) | 0/61 (0%) | 0.495935 | 0.945376 |
| PHF6 | 1/62 (1.61%) | 3/61 (4.92%) | 0.364711 | 0.945376 |
| PTPN11 | 1/62 (1.61%) | 4/61 (6.56%) | 0.207224 | 0.945376 |
| PUF60 | 1/62 (1.61%) | 0/61 (0%) | 1.000000 | 1.000000 |
| RAD21 | 0/62 (0%) | 3/61 (4.92%) | 0.118928 | 0.945376 |
| RHNO1 | 0/62 (0%) | 1/61 (1.64%) | 0.495935 | 0.945376 |
| RUNX1 | 9/62 (14.52%) | 10/61 (16.39%) | 0.807680 | 1.000000 |
| SF3B1 | 6/62 (9.68%) | 6/61 (9.84%) | 1.000000 | 1.000000 |
| SMC1A | 0/62 (0%) | 3/61 (4.92%) | 0.118928 | 0.945376 |
| SMC3 | 0/62 (0%) | 1/61 (1.64%) | 0.495935 | 0.945376 |
| SMG1 | 1/62 (1.61%) | 2/61 (3.28%) | 0.618827 | 1.000000 |
| SRSF2 | 2/62 (3.23%) | 6/61 (9.84%) | 0.163489 | 0.945376 |
| STAG2 | 0/62 (0%) | 1/61 (1.64%) | 0.495935 | 0.945376 |
| SUZ12 | 1/62 (1.61%) | 1/61 (1.64%) | 1.000000 | 1.000000 |
| TENM2 | 1/62 (1.61%) | 0/61 (0%) | 1.000000 | 1.000000 |
| TET2 | 7/62 (11.29%) | 10/61 (16.39%) | 0.445194 | 0.945376 |
| TP53 | 0/62 (0%) | 0/61 (0%) | 1.000000 | 1.000000 |
| TPTE2 | 2/62 (3.23%) | 1/61 (1.64%) | 1.000000 | 1.000000 |
| U2AF1 | 3/62 (4.84%) | 6/61 (9.84%) | 0.323067 | 0.945376 |
| WT1 | 5/62 (8.06%) | 6/61 (9.84%) | 0.762688 | 1.000000 |
| ZBTB47 | 1/62 (1.61%) | 0/61 (0%) | 1.000000 | 1.000000 |
| ZNF687 | 0/62 (0%) | 2/61 (3.28%) | 0.243902 | 0.945376 |
| ZNF711 | 2/62 (3.23%) | 0/61 (0%) | 0.495935 | 0.945376 |
| ZRSR2 | 0/62 (0%) | 3/61 (4.92%) | 0.118928 | 0.945376 |
|  |  |  |  |  |

| Supplementary table 4C  Somatic mutations in the Beat AML sample cohort (FLT3-ITD negative) | | | | |
| --- | --- | --- | --- | --- |
| **Gene** | **Female** | **Male** | **p-value** | **Adj. p-value** |
| ACAN | 1/160 (0.63%) | 3/215 (1.4%) | 0.639117 | 0.829493 |
| ADAMTS7 | 2/160 (1.25%) | 1/215 (0.47%) | 0.577835 | 0.829493 |
| ARID2 | 1/160 (0.63%) | 3/215 (1.4%) | 0.639117 | 0.829493 |
| ASXL1 | 10/160 (6.25%) | 25/215 (11.63%) | 0.105224 | 0.534888 |
| BCOR | 9/160 (5.63%) | 17/215 (7.91%) | 0.419535 | 0.829493 |
| BCORL1 | 3/160 (1.88%) | 7/215 (3.26%) | 0.526412 | 0.829493 |
| BHLHE22 | 1/160 (0.63%) | 3/215 (1.4%) | 0.639117 | 0.829493 |
| BRCC3 | 0/160 (0%) | 5/215 (2.33%) | 0.074354 | 0.520775 |
| CBL | 3/160 (1.88%) | 4/215 (1.86%) | 1.000000 | 1.000000 |
| CCND3 | 2/160 (1.25%) | 1/215 (0.47%) | 0.577835 | 0.829493 |
| CEBPA | 9/160 (5.63%) | 21/215 (9.77%) | 0.178714 | 0.641268 |
| CELSR2 | 2/160 (1.25%) | 1/215 (0.47%) | 0.577835 | 0.829493 |
| CNOT3 | 0/160 (0%) | 2/215 (0.93%) | 0.509447 | 0.829493 |
| CREBBP | 2/160 (1.25%) | 2/215 (0.93%) | 1.000000 | 1.000000 |
| CSF3R | 4/160 (2.5%) | 4/215 (1.86%) | 0.727976 | 0.863679 |
| DNMT3A | 35/160 (21.88%) | 36/215 (16.74%) | 0.231442 | 0.743051 |
| DSCAM | 1/160 (0.63%) | 3/215 (1.4%) | 0.639117 | 0.829493 |
| EZH2 | 2/160 (1.25%) | 12/215 (5.58%) | 0.029781 | 0.302772 |
| FAM194A | 3/160 (1.88%) | 3/215 (1.4%) | 0.703212 | 0.857919 |
| FLT3 | 20/160 (12.5%) | 18/215 (8.37%) | 0.226398 | 0.743051 |
| GATA2 | 9/160 (5.63%) | 7/215 (3.26%) | 0.306349 | 0.829493 |
| GNAS | 1/160 (0.63%) | 3/215 (1.4%) | 0.639117 | 0.829493 |
| IDH1 | 13/160 (8.13%) | 19/215 (8.84%) | 0.853730 | 0.946864 |
| IDH2 | 25/160 (15.63%) | 27/215 (12.56%) | 0.450719 | 0.829493 |
| IGFN1 | 1/160 (0.63%) | 1/215 (0.47%) | 1.000000 | 1.000000 |
| IKZF1 | 3/160 (1.88%) | 1/215 (0.47%) | 0.316820 | 0.829493 |
| JAK2 | 4/160 (2.5%) | 11/215 (5.12%) | 0.287669 | 0.829493 |
| JAK3 | 3/160 (1.88%) | 3/215 (1.4%) | 0.703212 | 0.857919 |
| KIT | 3/160 (1.88%) | 7/215 (3.26%) | 0.526412 | 0.829493 |
| KRAS | 10/160 (6.25%) | 9/215 (4.19%) | 0.476140 | 0.829493 |
| MPL | 4/160 (2.5%) | 1/215 (0.47%) | 0.168316 | 0.641268 |
| MXRA5 | 5/160 (3.13%) | 1/215 (0.47%) | 0.087714 | 0.527105 |
| MYC | 3/160 (1.88%) | 2/215 (0.93%) | 0.654681 | 0.831991 |
| NF1 | 4/160 (2.5%) | 2/215 (0.93%) | 0.408291 | 0.829493 |
| NPM1 | 33/160 (20.63%) | 30/215 (13.95%) | 0.095052 | 0.527105 |
| NRAS | 31/160 (19.38%) | 34/215 (15.81%) | 0.408769 | 0.829493 |
| ODF1 | 1/160 (0.63%) | 2/215 (0.93%) | 1.000000 | 1.000000 |
| PDS5B | 5/160 (3.13%) | 4/215 (1.86%) | 0.504686 | 0.829493 |
| PHF6 | 4/160 (2.5%) | 7/215 (3.26%) | 0.764568 | 0.863679 |
| PTPN11 | 10/160 (6.25%) | 9/215 (4.19%) | 0.476140 | 0.829493 |
| PUF60 | 0/160 (0%) | 5/215 (2.33%) | 0.074354 | 0.520775 |
| RAD21 | 5/160 (3.13%) | 5/215 (2.33%) | 0.749613 | 0.863679 |
| RHNO1 | 2/160 (1.25%) | 2/215 (0.93%) | 1.000000 | 1.000000 |
| RUNX1 | 9/160 (5.63%) | 31/215 (14.42%) | 0.006575 | 0.206752 |
| SF3B1 | 6/160 (3.75%) | 4/215 (1.86%) | 0.335945 | 0.829493 |
| SMC1A | 4/160 (2.5%) | 1/215 (0.47%) | 0.168316 | 0.641268 |
| SMC3 | 1/160 (0.63%) | 7/215 (3.26%) | 0.145250 | 0.632877 |
| SMG1 | 0/160 (0%) | 2/215 (0.93%) | 0.509447 | 0.829493 |
| SRSF2 | 14/160 (8.75%) | 40/215 (18.6%) | 0.007392 | 0.206752 |
| STAG2 | 10/160 (6.25%) | 19/215 (8.84%) | 0.435867 | 0.829493 |
| SUZ12 | 1/160 (0.63%) | 4/215 (1.86%) | 0.398529 | 0.829493 |
| TENM2 | 1/160 (0.63%) | 4/215 (1.86%) | 0.398529 | 0.829493 |
| TET2 | 18/160 (11.25%) | 26/215 (12.09%) | 0.871844 | 0.949687 |
| TP53 | 21/160 (13.13%) | 25/215 (11.63%) | 0.750642 | 0.863679 |
| TPTE2 | 2/160 (1.25%) | 1/215 (0.47%) | 0.577835 | 0.829493 |
| U2AF1 | 2/160 (1.25%) | 15/215 (6.98%) | 0.010168 | 0.206752 |
| WT1 | 19/160 (11.88%) | 11/215 (5.12%) | 0.020715 | 0.267217 |
| ZBTB47 | 0/160 (0%) | 4/215 (1.86%) | 0.139189 | 0.632877 |
| ZNF687 | 1/160 (0.63%) | 3/215 (1.4%) | 0.639117 | 0.829493 |
| ZNF711 | 3/160 (1.88%) | 0/215 (0%) | 0.076836 | 0.520775 |
| ZRSR2 | 0/160 (0%) | 7/215 (3.26%) | 0.021903 | 0.267217 |
|  |  |  |  |  |

| Supplementary table 4D  Somatic mutations in the Beat AML sample cohort (not-transformed) | | | | |
| --- | --- | --- | --- | --- |
| **Gene** | **Female** | **Male** | **p-value** | **Adj. p-value** |
| ACAN | 1/197 (0.51%) | 3/215 (1.4%) | 0.624613 | 0.929302 |
| ADAMTS7 | 3/197 (1.52%) | 2/215 (0.93%) | 0.673376 | 0.955255 |
| ARID2 | 2/197 (1.02%) | 2/215 (0.93%) | 1.000000 | 1.000000 |
| ASXL1 | 8/197 (4.06%) | 14/215 (6.51%) | 0.283614 | 0.845764 |
| BCOR | 6/197 (3.05%) | 16/215 (7.44%) | 0.051115 | 0.386742 |
| BCORL1 | 1/197 (0.51%) | 7/215 (3.26%) | 0.069740 | 0.386742 |
| BHLHE22 | 1/197 (0.51%) | 2/215 (0.93%) | 1.000000 | 1.000000 |
| BRCC3 | 0/197 (0%) | 5/215 (2.33%) | 0.062173 | 0.386742 |
| CBL | 2/197 (1.02%) | 2/215 (0.93%) | 1.000000 | 1.000000 |
| CCND3 | 2/197 (1.02%) | 5/215 (2.33%) | 0.452283 | 0.919643 |
| CEBPA | 11/197 (5.58%) | 18/215 (8.37%) | 0.336001 | 0.845764 |
| CELSR2 | 1/197 (0.51%) | 3/215 (1.4%) | 0.624613 | 0.929302 |
| CNOT3 | 2/197 (1.02%) | 1/215 (0.47%) | 0.608333 | 0.929302 |
| CREBBP | 3/197 (1.52%) | 2/215 (0.93%) | 0.673376 | 0.955255 |
| CSF3R | 3/197 (1.52%) | 5/215 (2.33%) | 0.726005 | 0.984140 |
| DNMT3A | 51/197 (25.89%) | 47/215 (21.86%) | 0.355778 | 0.845764 |
| DSCAM | 0/197 (0%) | 2/215 (0.93%) | 0.499740 | 0.923762 |
| EZH2 | 2/197 (1.02%) | 6/215 (2.79%) | 0.288050 | 0.845764 |
| FAM194A | 3/197 (1.52%) | 3/215 (1.4%) | 1.000000 | 1.000000 |
| FLT3 | 73/197 (37.06%) | 68/215 (31.63%) | 0.254644 | 0.845764 |
| GATA2 | 9/197 (4.57%) | 5/215 (2.33%) | 0.278322 | 0.845764 |
| GNAS | 1/197 (0.51%) | 0/215 (0%) | 0.478155 | 0.923762 |
| IDH1 | 17/197 (8.63%) | 16/215 (7.44%) | 0.718077 | 0.984140 |
| IDH2 | 25/197 (12.69%) | 26/215 (12.09%) | 0.881836 | 1.000000 |
| IGFN1 | 0/197 (0%) | 2/215 (0.93%) | 0.499740 | 0.923762 |
| IKZF1 | 1/197 (0.51%) | 1/215 (0.47%) | 1.000000 | 1.000000 |
| JAK2 | 1/197 (0.51%) | 3/215 (1.4%) | 0.624613 | 0.929302 |
| JAK3 | 3/197 (1.52%) | 1/215 (0.47%) | 0.352648 | 0.845764 |
| KIT | 3/197 (1.52%) | 7/215 (3.26%) | 0.342493 | 0.845764 |
| KRAS | 9/197 (4.57%) | 7/215 (3.26%) | 0.611908 | 0.929302 |
| MPL | 1/197 (0.51%) | 1/215 (0.47%) | 1.000000 | 1.000000 |
| MXRA5 | 5/197 (2.54%) | 0/215 (0%) | 0.024334 | 0.386742 |
| MYC | 4/197 (2.03%) | 2/215 (0.93%) | 0.431819 | 0.908309 |
| NF1 | 5/197 (2.54%) | 0/215 (0%) | 0.024334 | 0.386742 |
| NPM1 | 56/197 (28.43%) | 46/215 (21.4%) | 0.110101 | 0.559679 |
| NRAS | 29/197 (14.72%) | 27/215 (12.56%) | 0.566302 | 0.929302 |
| ODF1 | 2/197 (1.02%) | 2/215 (0.93%) | 1.000000 | 1.000000 |
| PDS5B | 7/197 (3.55%) | 3/215 (1.4%) | 0.204777 | 0.845764 |
| PHF6 | 2/197 (1.02%) | 10/215 (4.65%) | 0.037977 | 0.386742 |
| PTPN11 | 10/197 (5.08%) | 12/215 (5.58%) | 1.000000 | 1.000000 |
| PUF60 | 1/197 (0.51%) | 4/215 (1.86%) | 0.374354 | 0.845764 |
| RAD21 | 5/197 (2.54%) | 7/215 (3.26%) | 0.773734 | 1.000000 |
| RHNO1 | 2/197 (1.02%) | 3/215 (1.4%) | 1.000000 | 1.000000 |
| RUNX1 | 14/197 (7.11%) | 25/215 (11.63%) | 0.131412 | 0.616626 |
| SF3B1 | 8/197 (4.06%) | 6/215 (2.79%) | 0.589496 | 0.929302 |
| SMC1A | 3/197 (1.52%) | 4/215 (1.86%) | 1.000000 | 1.000000 |
| SMC3 | 1/197 (0.51%) | 7/215 (3.26%) | 0.069740 | 0.386742 |
| SMG1 | 1/197 (0.51%) | 4/215 (1.86%) | 0.374354 | 0.845764 |
| SRSF2 | 11/197 (5.58%) | 28/215 (13.02%) | 0.011230 | 0.386742 |
| STAG2 | 9/197 (4.57%) | 10/215 (4.65%) | 1.000000 | 1.000000 |
| SUZ12 | 2/197 (1.02%) | 3/215 (1.4%) | 1.000000 | 1.000000 |
| TENM2 | 2/197 (1.02%) | 3/215 (1.4%) | 1.000000 | 1.000000 |
| TET2 | 22/197 (11.17%) | 23/215 (10.7%) | 1.000000 | 1.000000 |
| TP53 | 17/197 (8.63%) | 15/215 (6.98%) | 0.583102 | 0.929302 |
| TPTE2 | 4/197 (2.03%) | 2/215 (0.93%) | 0.431819 | 0.908309 |
| U2AF1 | 4/197 (2.03%) | 14/215 (6.51%) | 0.030092 | 0.386742 |
| WT1 | 23/197 (11.68%) | 17/215 (7.91%) | 0.243743 | 0.845764 |
| ZBTB47 | 1/197 (0.51%) | 4/215 (1.86%) | 0.374354 | 0.845764 |
| ZNF687 | 1/197 (0.51%) | 4/215 (1.86%) | 0.374354 | 0.845764 |
| ZNF711 | 4/197 (2.03%) | 0/215 (0%) | 0.051441 | 0.386742 |
| ZRSR2 | 0/197 (0%) | 5/215 (2.33%) | 0.062173 | 0.386742 |
|  |  |  |  |  |
|  |  |  |  |  |

| Supplementary table 5  Somatic mutations in the LAML-TCGA cohort | | | | |
| --- | --- | --- | --- | --- |
| **Gene** | **Female** | **Male** | **p-value** | **Adj. p-value** |
|  |  |  |  |  |
| ASXL1 | 3/92 (3.26%) | 2/108 (1.85%) | 0.662967286 | 0.999319295 |
| CEBPA | 5/92 (5.43%) | 8/108 (7.41%) | 0.774896464 | 0.999319295 |
| DNMT3A | 27/92 (29.35%) | 24/108 (22.22%) | 0.25951164 | 0.875851786 |
| FAM5C | 1/92 (1.09%) | 4/108 (3.7%) | 0.376807434 | 0.924890973 |
| FCGBP | 1/92 (1.09%) | 4/108 (3.7%) | 0.376807434 | 0.924890973 |
| FLT3 | 27/92 (29.35%) | 29/108 (26.85%) | 0.752900268 | 0.999319295 |
| IDH1 | 10/92 (10.87%) | 9/108 (8.33%) | 0.631221142 | 0.999319295 |
| IDH2 | 10/92 (10.87%) | 10/108 (9.26%) | 0.814260167 | 0.999319295 |
| KIT | 5/92 (5.43%) | 3/108 (2.78%) | 0.47417479 | 0.999319295 |
| KRAS | 6/92 (6.52%) | 2/108 (1.85%) | 0.146583639 | 0.791551653 |
| MT.CO2 | 3/92 (3.26%) | 5/108 (4.63%) | 0.728232035 | 0.999319295 |
| MT.CYB | 1/92 (1.09%) | 5/108 (4.63%) | 0.220764711 | 0.851521028 |
| MUC16 | 2/92 (2.17%) | 3/108 (2.78%) | 1 | 1 |
| NPM1 | 29/92 (31.52%) | 25/108 (23.15%) | 0.203434669 | 0.851521028 |
| NRAS | 7/92 (7.61%) | 8/108 (7.41%) | 1 | 1 |
| PHF6 | 0/92 (0%) | 6/108 (5.56%) | 0.03187315 | 0.791551653 |
| PTPN11 | 4/92 (4.35%) | 5/108 (4.63%) | 1 | 1 |
| RAD21 | 2/92 (2.17%) | 3/108 (2.78%) | 1 | 1 |
| RUNX1 | 8/92 (8.7%) | 11/108 (10.19%) | 0.811322445 | 0.999319295 |
| SMC1A | 1/92 (1.09%) | 6/108 (5.56%) | 0.12684558 | 0.791551653 |
| SMC3 | 4/92 (4.35%) | 3/108 (2.78%) | 0.70533024 | 0.999319295 |
| STAG2 | 4/92 (4.35%) | 3/108 (2.78%) | 0.70533024 | 0.999319295 |
| TET2 | 10/92 (10.87%) | 7/108 (6.48%) | 0.314446123 | 0.924890973 |
| TP53 | 4/92 (4.35%) | 12/108 (11.11%) | 0.11540285 | 0.791551653 |
| TTN | 3/92 (3.26%) | 5/108 (4.63%) | 0.728232035 | 0.999319295 |
| U2AF1 | 1/92 (1.09%) | 7/108 (6.48%) | 0.07180716 | 0.791551653 |
| WT1 | 5/92 (5.43%) | 7/108 (6.48%) | 1 | 1 |
|  |  |  |  |  |

| Supplementary table 6  Mutations categorized by gene product function  Beat-AML sample cohort (All) | | | | |
| --- | --- | --- | --- | --- |
| **Class** | **Female** | **Male** | **p-value** | **Adj. p-value** |
|  |  |  |  |  |
| RNA splicing gene | 32/222 (14.41%) | 85/276 (30.8%) | 1.84682E-05 | 0.000129278 |
| Epigenetic modifier gene | 20/222 (9.01%) | 60/276 (21.74%) | 0.000121956 | 0.000426846 |
| Signaling gene | 127/222 (57.21%) | 136/276 (49.28%) | 0.08641913 | 0.201644638 |
| DNA methylation gene | 119/222 (53.6%) | 135/276 (48.91%) | 0.321668112 | 0.562919196 |
| Cohesin complex gene | 22/222 (9.91%) | 32/276 (11.59%) | 0.565819085 | 0.71477474 |
| Tumor suppressors gene | 24/222 (10.81%) | 34/276 (12.32%) | 0.67403369 | 0.71477474 |
| Transcription factor gene | 93/222 (41.89%) | 111/276 (40.22%) | 0.71477474 | 0.71477474 |
|  |  |  |  |  |
|  |  |  |  |  |
|  |  |  |  |  |
| Beat-AML sample cohort (not transformed) | | | | |
| **Class** | **Female** | **Male** | **p-value** | **Adj. p-value** |
|  |  |  |  |  |
| RNA splicing gene | 23/197 (11.68%) | 52/215 (24.19%) | 0.001296133 | 0.009072931 |
| Epigenetic modifier gene | 15/197 (7.61%) | 35/215 (16.28%) | 0.009710185 | 0.033985647 |
| Signaling gene | 113/197 (57.36%) | 105/215 (48.84%) | 0.093291927 | 0.217681164 |
| DNA methylation gene | 107/197 (54.31%) | 103/215 (47.91%) | 0.201137385 | 0.351990423 |
| Cohesin complex gene | 19/197 (9.64%) | 24/215 (11.16%) | 0.632544327 | 0.841599733 |
| Tumor suppressors gene | 21/197 (10.66%) | 20/215 (9.3%) | 0.742283272 | 0.841599733 |
| Transcription factor gene | 83/197 (42.13%) | 88/215 (40.93%) | 0.841599733 | 0.841599733 |
|  |  |  |  |  |
|  |  |  |  |  |
|  |  |  |  |  |
| Beat-AML sample cohort (*FLT3*-ITD negative) | | | | |
| **Class** | **Female** | **Male** | **p-value** | **Adj. p-value** |
|  |  |  |  |  |
| RNA splicing gene | 22/160 (13.75%) | 65/215 (30.23%) | 0.000188435 | 0.001319047 |
| Epigenetic modifier gene | 18/160 (11.25%) | 50/215 (23.26%) | 0.0028385 | 0.00993475 |
| Signaling gene | 84/160 (52.5%) | 98/215 (45.58%) | 0.21021417 | 0.49049973 |
| DNA methylation gene | 68/160 (42.5%) | 83/215 (38.6%) | 0.45801146 | 0.724038472 |
| Cohesin complex gene | 55/160 (34.38%) | 81/215 (37.67%) | 0.517170337 | 0.724038472 |
| Tumor suppressors gene | 20/160 (12.5%) | 28/215 (13.02%) | 1 | 1 |
| Transcription factor gene | 23/160 (14.38%) | 31/215 (14.42%) | 1 | 1 |
|  |  |  |  |  |
|  |  |  |  |  |
|  |  |  |  |  |
| Beat-AML sample cohort (*FLT3*-ITD positive) | | | | |
| **Class** | **Female** | **Male** | **p-value** | **Adj. p-value** |
|  |  |  |  |  |
| RNA splicing gene | 2/62 (3.23%) | 10/61 (16.39%) | 0.016084581 | 0.112592065 |
| Epigenetic modifier gene | 10/62 (16.13%) | 20/61 (32.79%) | 0.037083499 | 0.129792247 |
| Signaling gene | 59/62 (95.16%) | 53/61 (86.89%) | 0.126122631 | 0.294286139 |
| DNA methylation gene | 38/62 (61.29%) | 30/61 (49.18%) | 0.206358703 | 0.36112773 |
| Cohesin complex gene | 1/62 (1.61%) | 3/61 (4.92%) | 0.364711416 | 0.510595982 |
| Tumor suppressors gene | 2/62 (3.23%) | 4/61 (6.56%) | 0.439451249 | 0.512693124 |
| Transcription factor gene | 35/62 (56.45%) | 37/61 (60.66%) | 0.715302299 | 0.715302299 |
|  |  |  |  |  |


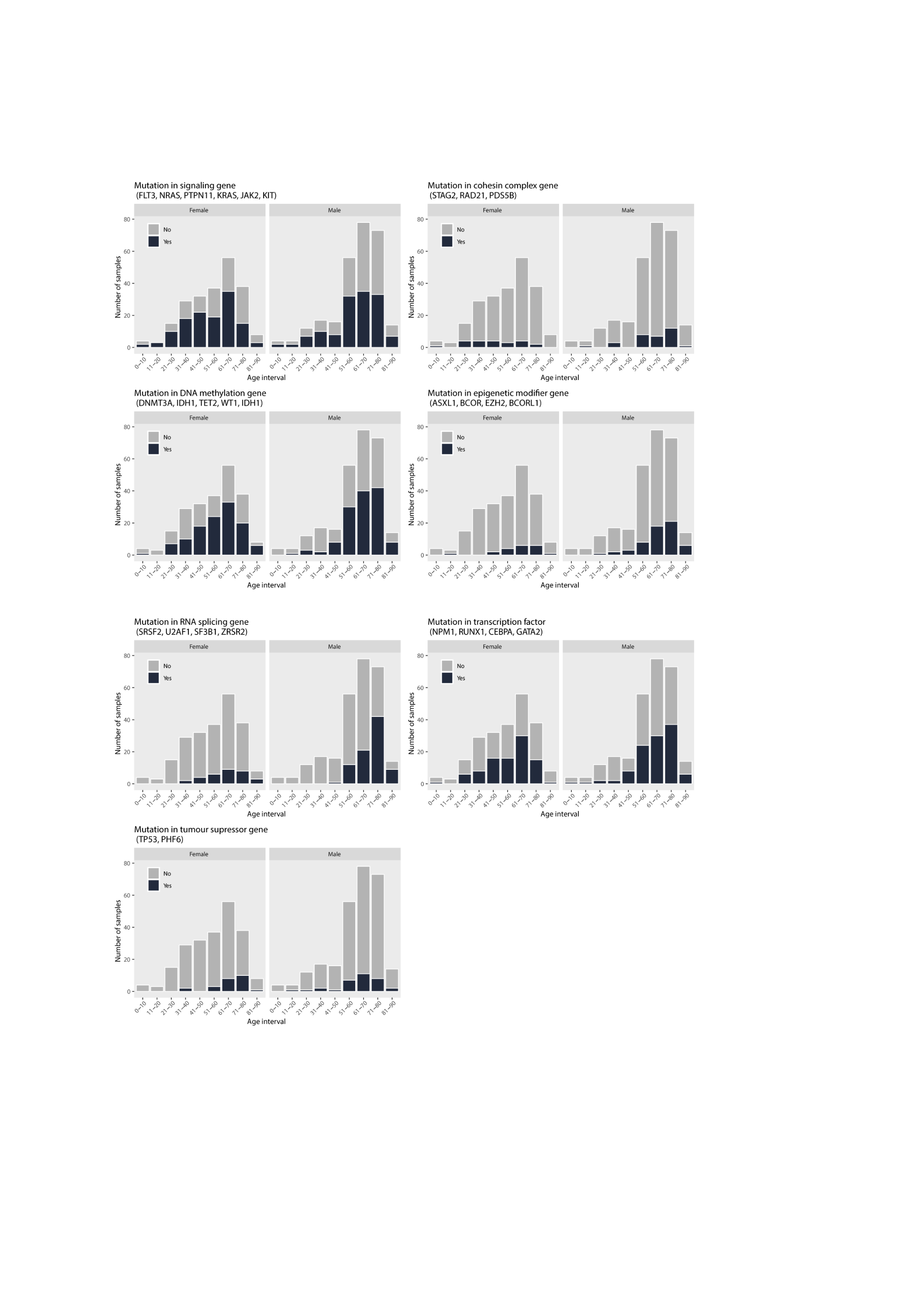


Supplementary figure 4: Age distribution of samples with at least one mutation within each class of mutations separated by female and male individuals in the Beat AML sample cohort. Raw values of this figure are presented in the tables on the following page.

| Activation signaling | | | | |  | Cohesin complex | | | | |
| --- | --- | --- | --- | --- | --- | --- | --- | --- | --- | --- |
| Age interval | Female | Male | p-value | Adj. P-value |  | Age interval | Female | Male | p-value | Adj. P-value |
| 0-10 | 2/4 (50%) | 2/4 (50%) | 1 | 1 |  | 0-10 | 1/4 (25%) | 0/4 (0%) | 1 | 1 |
| 11-20 | 3/3 (100%) | 2/4 (50%) | 0.428571429 | 1 |  | 11-20 | 0/3 (0%) | 1/4 (25%) | 1 | 1 |
| 21-30 | 10/15 (66.7%) | 7/12 (58.3%) | 0.706331045 | 1 |  | 21-30 | 4/15 (26.7%) | 0/12 (0%) | 0.105982906 | 0.936359306 |
| 31-40 | 18/29 (62.1%) | 10/17 (58.8%) | 1 | 1 |  | 31-40 | 4/29 (13.8%) | 3/17 (17.6%) | 1 | 1 |
| 41-50 | 22/32 (68.8%) | 8/16 (50%) | 0.225765254 | 0.990144928 |  | 41-50 | 4/32 (12.5%) | 0/16 (0%) | 0.28625758 | 1 |
| 51-60 | 19/37 (51.4%) | 32/56 (57.1%) | 0.671732856 | 1 |  | 51-60 | 3/37 (8.1%) | 8/56 (14.3%) | 0.516593262 | 1 |
| 61-70 | 35/56 (62.5%) | 35/78 (44.9%) | 0.054280264 | 0.936359306 |  | 61-70 | 4/56 (7.1%) | 7/78 (9%) | 0.76131245 | 1 |
| 71-80 | 15/38 (39.5%) | 33/73 (45.2%) | 0.686862226 | 1 |  | 71-80 | 2/38 (5.3%) | 12/73 (16.4%) | 0.132919361 | 0.936359306 |
| 81-90 | 3/8 (37.5%) | 7/14 (50%) | 0.674922601 | 1 |  | 81-90 | 0/8 (0%) | 1/14 (7.1%) | 1 | 1 |
|  |  |  |  |  |  |  |  |  |  |  |
|  |  |  |  |  |  |  |  |  |  |  |
| DNA methylation | | | | |  | Epigenetic modifiers | | | | |
| Age interval | Female | Male | p-value | Adj. P-value |  | Age interval | Female | Male | p-value | Adj. P-value |
| 0-10 | 1/4 (25%) | 0/4 (0%) | 1 | 1 |  | 0-10 | 0/4 (0%) | 0/4 (0%) | 1 | 1 |
| 11-20 | 0/3 (0%) | 1/4 (25%) | 1 | 1 |  | 11-20 | 1/3 (33.3%) | 0/4 (0%) | 0.428571429 | 1 |
| 21-30 | 7/15 (46.7%) | 3/12 (25%) | 0.424408848 | 1 |  | 21-30 | 0/15 (0%) | 1/12 (8.3%) | 0.444444444 | 1 |
| 31-40 | 10/29 (34.5%) | 2/17 (11.8%) | 0.163147115 | 0.936359306 |  | 31-40 | 0/29 (0%) | 2/17 (11.8%) | 0.131400966 | 0.936359306 |
| 41-50 | 18/32 (56.3%) | 8/16 (50%) | 0.763676736 | 1 |  | 41-50 | 2/32 (6.3%) | 3/16 (18.8%) | 0.316383072 | 1 |
| 51-60 | 24/37 (64.9%) | 30/56 (53.6%) | 0.293813921 | 1 |  | 51-60 | 4/37 (10.8%) | 8/56 (14.3%) | 0.757328396 | 1 |
| 61-70 | 33/56 (58.9%) | 40/78 (51.3%) | 0.481968061 | 1 |  | 61-70 | 6/56 (10.7%) | 18/78 (23.1%) | 0.072352619 | 0.936359306 |
| 71-80 | 20/38 (52.6%) | 42/73 (57.5%) | 0.688932234 | 1 |  | 71-80 | 6/38 (15.8%) | 21/73 (28.8%) | 0.164596622 | 0.936359306 |
| 81-90 | 6/8 (75%) | 8/14 (57.1%) | 0.649398005 | 1 |  | 81-90 | 1/8 (12.5%) | 6/14 (42.9%) | 0.193217 | 0.936359306 |
|  |  |  |  |  |  |  |  |  |  |  |
|  |  |  |  |  |  |  |  |  |  |  |
| RNA splicing | | | | |  | Transcription factor | | | | |
| Age interval | Female | Male | p-value | Adj. P-value |  | Age interval | Female | Male | p-value | Adj. P-value |
| 0-10 | 0/4 (0%) | 0/4 (0%) | 1 | 1 |  | 0-10 | 1/4 (25%) | 1/4 (25%) | 1 | 1 |
| 11-20 | 0/3 (0%) | 0/4 (0%) | 1 | 1 |  | 11-20 | 0/3 (0%) | 1/4 (25%) | 1 | 1 |
| 21-30 | 0/15 (0%) | 0/12 (0%) | 1 | 1 |  | 21-30 | 6/15 (40%) | 2/12 (16.7%) | 0.235748792 | 0.990144928 |
| 31-40 | 2/29 (6.9%) | 0/17 (0%) | 0.523671498 | 1 |  | 31-40 | 8/29 (27.6%) | 2/17 (11.8%) | 0.282021014 | 1 |
| 41-50 | 4/32 (12.5%) | 1/16 (6.3%) | 0.652398172 | 1 |  | 41-50 | 16/32 (50%) | 8/16 (50%) | 1 | 1 |
| 51-60 | 6/37 (16.2%) | 12/56 (21.4%) | 0.600736315 | 1 |  | 51-60 | 16/37 (43.2%) | 24/56 (42.9%) | 1 | 1 |
| 61-70 | 9/56 (16.1%) | 21/78 (26.9%) | 0.148534562 | 0.936359306 |  | 61-70 | 30/56 (53.6%) | 30/78 (38.5%) | 0.112698095 | 0.936359306 |
| 71-80 | 8/38 (21.1%) | 42/73 (57.5%) | **0.000273976** | **0.017260461** |  | 71-80 | 15/38 (39.5%) | 37/73 (50.7%) | 0.317917699 | 1 |
| 81-90 | 3/8 (37.5%) | 9/14 (64.3%) | 0.377708978 | 1 |  | 81-90 | 1/8 (12.5%) | 6/14 (42.9%) | 0.193217 | 0.936359306 |
|  |  |  |  |  |  |  |  |  |  |  |
|  |  |  |  |  |  |  |  |  |  |  |
| Tumor suppressors | | | | |  |  |  |  |  |  |
| Age interval | Female | Male | p-value | Adj. P-value |  |  |  |  |  |  |
| 0-10 | 0/4 (0%) | 0/4 (0%) | 1 | 1 |  |  |  |  |  |  |
| 11-20 | 0/3 (0%) | 1/4 (25%) | 1 | 1 |  |  |  |  |  |  |
| 21-30 | 0/15 (0%) | 1/12 (8.3%) | 0.444444444 | 1 |  |  |  |  |  |  |
| 31-40 | 2/29 (6.9%) | 2/17 (11.8%) | 0.619340013 | 1 |  |  |  |  |  |  |
| 41-50 | 0/32 (0%) | 1/16 (6.3%) | 0.333333333 | 1 |  |  |  |  |  |  |
| 51-60 | 3/37 (8.1%) | 7/56 (12.5%) | 0.734587682 | 1 |  |  |  |  |  |  |
| 61-70 | 8/56 (14.3%) | 11/78 (14.1%) | 1 | 1 |  |  |  |  |  |  |
| 71-80 | 10/38 (26.3%) | 8/73 (11%) | 0.055879789 | 0.936359306 |  |  |  |  |  |  |
| 81-90 | 1/8 (12.5%) | 2/14 (14.3%) | 1 | 1 |  |  |  |  |  |  |
|  |  |  |  |  |  |  |  |  |  |  |

| Supplementary table 7  All cohorts | | | | |
| --- | --- | --- | --- | --- |
| **Composition** | **Female** | **Male** | **p-value** | **Adj. p-value** |
|  |  |  |  |  |
| DNMT3A | 63/748 (8.42%) | 78/876 (8.9%) | 0.790966684 | 0.903961925 |
| FLT3-ITD | 74/748 (9.89%) | 95/876 (10.84%) | 0.568520413 | 0.758027217 |
| FLT3-ITD + DNMT3A | 13/748 (1.74%) | 16/876 (1.83%) | 1 | 1 |
| FLT3-ITD + NPM1 | 56/748 (7.49%) | 40/876 (4.57%) | 0.014952179 | 0.039872477 |
| FLT3-ITD + NPM1 + DNMT3A | 70/748 (9.36%) | 43/876 (4.91%) | 0.00056669 | 0.004037681 |
| FLT3-ITD, NPM1 and DNMT3A negative | 347/748 (46.39%) | 479/876 (54.68%) | 0.00100942 | 0.004037681 |
| NPM1 | 62/748 (8.29%) | 63/876 (7.19%) | 0.455129014 | 0.728206422 |
| NPM1 + DNMT3A | 63/748 (8.42%) | 62/876 (7.08%) | 0.350418967 | 0.700837934 |
|  |  |  |  |  |
|  |  |  |  |  |
| Beat AML sample cohort | | | | |
| **Composition** | **Female** | **Male** | **p-value** | **Adj. p-value** |
|  |  |  |  |  |
| DNMT3A | 21/222 (9.46%) | 22/276 (7.97%) | 0.630890704 | 0.680521527 |
| FLT3-ITD | 29/222 (13.06%) | 32/276 (11.59%) | 0.680521527 | 0.680521527 |
| FLT3-ITD + DNMT3A | 4/222 (1.8%) | 9/276 (3.26%) | 0.401811449 | 0.680521527 |
| FLT3-ITD + NPM1 | 11/222 (4.95%) | 11/276 (3.99%) | 0.663778999 | 0.680521527 |
| FLT3-ITD + NPM1 + DNMT3A | 18/222 (8.11%) | 9/276 (3.26%) | 0.026849035 | 0.107396138 |
| FLT3-ITD, NPM1 and DNMT3A negative | 106/222 (47.75%) | 163/276 (59.06%) | 0.014529124 | 0.107396138 |
| NPM1 | 19/222 (8.56%) | 16/276 (5.8%) | 0.290055356 | 0.680521527 |
| NPM1 + DNMT3A | 14/222 (6.31%) | 14/276 (5.07%) | 0.563540687 | 0.680521527 |
|  |  |  |  |  |
|  |  |  |  |  |
| LAML-TCGA | | | | |
| **Composition** | **Female** | **Male** | **p-value** | **Adj. p-value** |
|  |  |  |  |  |
| DNMT3A | 10/92 (10.87%) | 11/108 (10.19%) | 1 | 1 |
| FLT3-ITD | 6/92 (6.52%) | 12/108 (11.11%) | 0.325057011 | 1 |
| FLT3-ITD + DNMT3A | 1/92 (1.09%) | 1/108 (0.93%) | 1 | 1 |
| FLT3-ITD + NPM1 | 5/92 (5.43%) | 4/108 (3.7%) | 0.735026234 | 1 |
| FLT3-ITD + NPM1 + DNMT3A | 6/92 (6.52%) | 4/108 (3.7%) | 0.517683005 | 1 |
| FLT3-ITD, NPM1 and DNMT3A negative | 46/92 (50%) | 59/108 (54.63%) | 0.570617347 | 1 |
| NPM1 | 8/92 (8.7%) | 9/108 (8.33%) | 1 | 1 |
| NPM1 + DNMT3A | 10/92 (10.87%) | 8/108 (7.41%) | 0.461428454 | 1 |
|  |  |  |  |  |
|  |  |  |  |  |
| HOVON 1 | | | | |
| **Composition** | **Female** | **Male** | **p-value** | **Adj. p-value** |
|  |  |  |  |  |
| DNMT3A | 7/214 (3.27%) | 16/212 (7.55%) | 0.056014565 | 0.22405826 |
| FLT3-ITD | 19/214 (8.88%) | 22/212 (10.38%) | 0.625574877 | 0.839200514 |
| FLT3-ITD + DNMT3A | 2/214 (0.93%) | 2/212 (0.94%) | 1 | 1 |
| FLT3-ITD + NPM1 | 27/214 (12.62%) | 14/212 (6.6%) | 0.04770093 | 0.22405826 |
| FLT3-ITD + NPM1 + DNMT3A | 19/214 (8.88%) | 12/212 (5.66%) | 0.263035523 | 0.701428061 |
| FLT3-ITD, NPM1 and DNMT3A negative | 108/214 (50.47%) | 112/212 (52.83%) | 0.629400386 | 0.839200514 |
| NPM1 | 14/214 (6.54%) | 17/212 (8.02%) | 0.581134133 | 0.839200514 |
| NPM1 + DNMT3A | 18/214 (8.41%) | 17/212 (8.02%) | 1 | 1 |
|  |  |  |  |  |
|  |  |  |  |  |
| HOVON 2 | | | | |
| **Composition** | **Female** | **Male** | **p-value** | **Adj. p-value** |
|  |  |  |  |  |
| DNMT3A | 25/220 (11.36%) | 29/280 (10.36%) | 0.772280631 | 0.772280631 |
| FLT3-ITD | 20/220 (9.09%) | 29/280 (10.36%) | 0.653277574 | 0.746602941 |
| FLT3-ITD + DNMT3A | 6/220 (2.73%) | 4/280 (1.43%) | 0.347300383 | 0.67483001 |
| FLT3-ITD + NPM1 | 13/220 (5.91%) | 11/280 (3.93%) | 0.399721363 | 0.67483001 |
| FLT3-ITD + NPM1 + DNMT3A | 27/220 (12.27%) | 18/280 (6.43%) | 0.027435861 | 0.109743445 |
| FLT3-ITD, NPM1 and DNMT3A negative | 87/220 (39.55%) | 145/280 (51.79%) | 0.006822489 | 0.054579909 |
| NPM1 | 21/220 (9.55%) | 21/280 (7.5%) | 0.421768756 | 0.67483001 |
| NPM1 + DNMT3A | 21/220 (9.55%) | 23/280 (8.21%) | 0.635415799 | 0.746602941 |
|  |  |  |  |  |

Supplementary figure 5: Distribution of various combinations of FLT3-ITD, NPM1 and DNMT3A mutations separated by sex and by cohort.


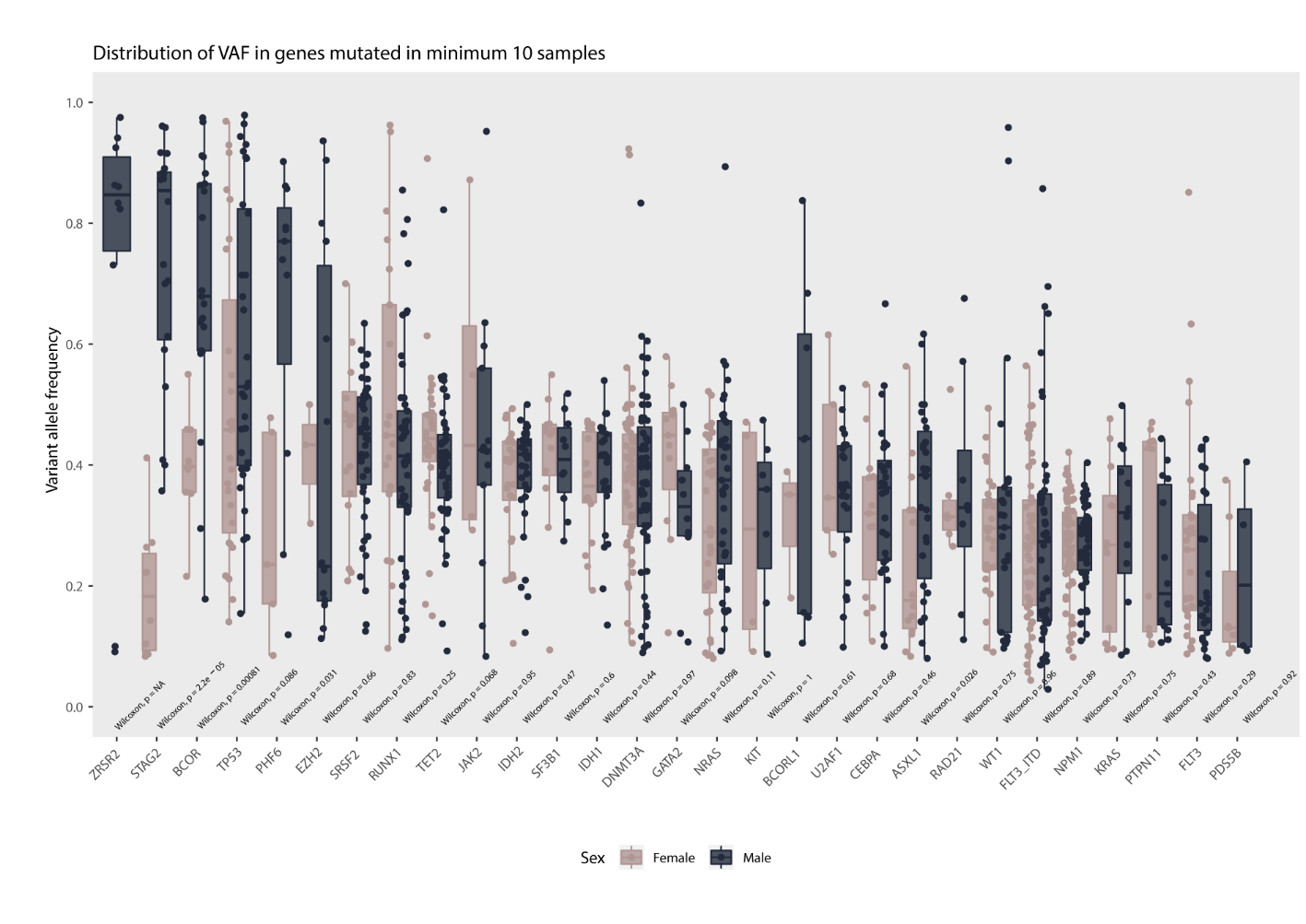


Supplementary figure 6: Box plot representing distribution of variant allele frequencies in the genes called as mutated at least 10 times in the sample selection. Each dot represents one variant within one sample. Pairwise comparison (Wilcoxon rank sum test/Mann-Whitney test) of VAF distribution between female and male individuals. The boxes represent the median and the spread. The lower and upper hinges correspond the 25th and 75th percentiles, respectively, and the upper and lower whisker extends to the largest and smaller values (no further than 1.5 times the total inter quartile range from the hinges).


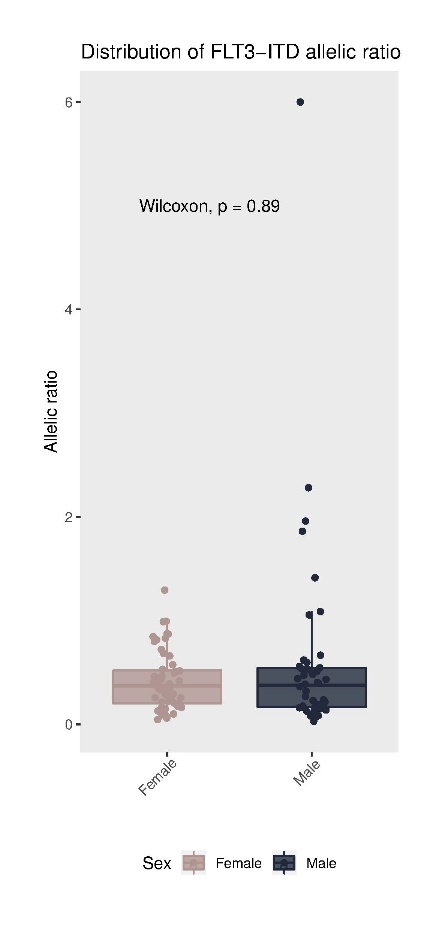


Supplementary figure 7: Box plot representing distribution of FLT3-ITD allelic ratio (AR) in females and males in the Beat AML cohort. The allelic ratio was calculated using the FLT3-ITD variant allele frequency (VAF) from next generation sequencing data by the following approach:

$VAF\times100=\% mutated allele$

$$100\div\% mutated allele=\% wt allele$$

$\frac{\% mutated allele}{\% wt allele}=AR$

Supplementary figure 8: Boxplot of the 17 mRNA transcripts that were identified as specifically differentially expressed in female and male FLT3-ITD positive AML.

Supplementary figure 9: Boxplot of the 17 mRNA transcripts that were identified as differentially expressed in female and male FLT3-ITD positive AML. Pairwise relationships comparing FLT3-ITD mutated samples and FLT3-ITD non-mutated samples demonstrates that most of the genes are differentially expressed.

| Supplementary table 8  BeatAML cohort DGE analysis | | | | | | | | | | |
| --- | --- | --- | --- | --- | --- | --- | --- | --- | --- | --- |
| Symbol | Gene | MAP | F:M (FLT3-ITD) | F:M | F:FLT3-ITD | M:FLT3-ITD | AveExpr | F | P.Value | adj.P.Val |
|  |  |  |  |  |  |  |  |  |  |  |
| AE000661.37 | ENSG00000251002 | NA | 1.196 | -0.023 | 2.445 | 1.225 | 2.925 | 15.800 | 0.000 | 0.000 |
| BCORP1 | ENSG00000215580 | Yq11.222 | -3.694 | -3.688 | -0.023 | -0.017 | -1.147 | 326.027 | 0.000 | 0.000 |
| CCL1 | ENSG00000108702 | 17q12 | 1.534 | -0.116 | 2.664 | 1.015 | -1.769 | 23.521 | 0.000 | 0.000 |
| DDX3Y | ENSG00000067048 | Yq11.221 | -10.307 | -9.977 | -0.048 | 0.282 | 3.480 | 2026.231 | 0.000 | 0.000 |
| DDX3YP1 | ENSG00000232928 | NA | -2.488 | -2.490 | 0.003 | 0.001 | -1.823 | 420.964 | 0.000 | 0.000 |
| EIF1AY | ENSG00000198692 | Yq11.223 | -7.771 | -8.011 | 0.037 | -0.202 | 1.574 | 1275.554 | 0.000 | 0.000 |
| FAT1 | ENSG00000083857 | 4q35.2 | -1.622 | -0.172 | -1.083 | 0.367 | -1.167 | 4.077 | 0.007 | 0.027 |
| GLI2 | ENSG00000074047 | 2q14.2 | 1.306 | -0.140 | 3.898 | 2.452 | -1.810 | 63.863 | 0.000 | 0.000 |
| GPR126 | ENSG00000112414 | NA | -1.697 | -0.225 | -1.548 | -0.076 | -0.189 | 6.232 | 0.000 | 0.003 |
| HMGA2 | ENSG00000149948 | 12q14.3 | -1.920 | -0.492 | -1.187 | 0.241 | -0.684 | 5.460 | 0.001 | 0.007 |
| HOXB-AS3 | ENSG00000233101 | 17q21.32 | 1.086 | 0.461 | 2.566 | 1.941 | 0.509 | 14.810 | 0.000 | 0.000 |
| IRX5 | ENSG00000176842 | 16q12.2 | 1.324 | 0.505 | 1.622 | 0.804 | -0.285 | 8.605 | 0.000 | 0.000 |
| JPH1 | ENSG00000104369 | 8q21.11 | 1.136 | 0.010 | 2.058 | 0.932 | -1.910 | 19.087 | 0.000 | 0.000 |
| KALP | ENSG00000241859 | NA | -4.431 | -4.177 | -0.049 | 0.205 | -0.844 | 710.174 | 0.000 | 0.000 |
| KDM5D | ENSG00000012817 | Yq11.223 | -10.018 | -9.835 | 0.038 | 0.221 | 2.791 | 2380.843 | 0.000 | 0.000 |
| LINC00278 | ENSG00000231535 | Yp11.2 | -3.636 | -3.933 | -0.027 | -0.324 | -1.076 | 593.646 | 0.000 | 0.000 |
| MDGA1 | ENSG00000112139 | 6p21.2 | 1.113 | -0.393 | 2.567 | 1.061 | 1.917 | 17.293 | 0.000 | 0.000 |
| MKRN3 | ENSG00000179455 | 15q11.2 | 1.365 | 0.615 | 0.488 | -0.262 | -0.493 | 3.784 | 0.011 | 0.037 |
| NETO1 | ENSG00000166342 | 18q22.3 | 1.624 | -0.312 | 1.461 | -0.475 | -1.562 | 5.970 | 0.001 | 0.004 |
| NLRP2 | ENSG00000022556 | 19q13.42 | 1.090 | 0.943 | 1.151 | 1.004 | 1.198 | 12.761 | 0.000 | 0.000 |
| PRKY | ENSG00000099725 | Yp11.2 | -6.664 | -6.403 | 0.076 | 0.337 | 2.761 | 1014.086 | 0.000 | 0.000 |
| RASGRF1 | ENSG00000058335 | 15q25.1 | 1.281 | 0.097 | 1.653 | 0.469 | -0.995 | 17.249 | 0.000 | 0.000 |
| RP11-424G14.1 | ENSG00000260197 | NA | -4.925 | -4.952 | -0.004 | -0.031 | -0.438 | 1080.247 | 0.000 | 0.000 |
| RP11-576C2.1 | ENSG00000267793 | NA | -2.482 | -2.171 | -0.020 | 0.291 | -2.028 | 251.441 | 0.000 | 0.000 |
| RP11-713C5.1 | ENSG00000265579 | NA | 1.167 | -0.198 | 1.235 | -0.130 | -2.356 | 6.523 | 0.000 | 0.002 |
| RPS4Y1 | ENSG00000129824 | Yp11.2 | -10.682 | -10.328 | -0.025 | 0.330 | 3.145 | 2281.647 | 0.000 | 0.000 |
| SCRN1 | ENSG00000136193 | 7p14.3 | -1.736 | -0.282 | -1.181 | 0.273 | 4.372 | 5.923 | 0.001 | 0.004 |
| SIGLEC6 | ENSG00000105492 | 19q13.41 | 1.107 | -0.060 | 1.053 | -0.114 | 0.797 | 4.193 | 0.006 | 0.024 |
| TMSB4Y | ENSG00000154620 | Yq11.221 | -4.487 | -4.015 | -0.291 | 0.180 | -0.296 | 732.730 | 0.000 | 0.000 |
| TSIX | ENSG00000270641 | Xq13.2 | 3.769 | 3.260 | 0.566 | 0.057 | -1.774 | 508.937 | 0.000 | 0.000 |
| TTTY10 | ENSG00000229236 | Yq11.223 | -2.627 | -2.801 | -0.023 | -0.197 | -1.712 | 288.157 | 0.000 | 0.000 |
| TTTY14 | ENSG00000176728 | Yq11.222 | -4.718 | -4.586 | -0.020 | 0.112 | -0.639 | 584.468 | 0.000 | 0.000 |
| TTTY15 | ENSG00000233864 | Yq11.221 | -7.444 | -7.077 | 0.027 | 0.393 | 0.960 | 1155.479 | 0.000 | 0.000 |
| TXLNG2P | ENSG00000131002 | NA | -9.381 | -9.205 | 0.020 | 0.196 | 2.327 | 1643.162 | 0.000 | 0.000 |
| UGT3A2 | ENSG00000168671 | 5p13.2 | 1.227 | -0.759 | 1.132 | -0.853 | -0.176 | 4.385 | 0.005 | 0.020 |
| USP9Y | ENSG00000114374 | Yq11.221 | -8.344 | -8.088 | 0.092 | 0.349 | 1.901 | 1455.333 | 0.000 | 0.000 |
| UTY | ENSG00000183878 | Yq11.221 | -8.374 | -8.196 | 0.055 | 0.234 | 1.815 | 2357.931 | 0.000 | 0.000 |
| XIST | ENSG00000229807 | Xq13.2 | 12.235 | 11.653 | 0.453 | -0.129 | 3.566 | 2346.787 | 0.000 | 0.000 |
| ZFY | ENSG00000067646 | Yp11.2 | -7.953 | -7.600 | 0.071 | 0.424 | 1.453 | 1832.711 | 0.000 | 0.000 |
|  |  |  |  |  |  |  |  |  |  |  |

| Supplementary table 9  Univariare Cox regression analysis (Beat AML sample cohort, n=303) | | | | |
| --- | --- | --- | --- | --- |
| **Gene** | **Beta** | **HR (95% CI for HR)** | **Wald Test** | **p-value** |
| AE000661_37 | -0.0467 | 0.954 (0.89-1) | 1.65 | 0.199 |
| BCORP1 | 0.0659 | 1.07 (0.98-1.2) | 2.38 | 0.123 |
| CCL1 | 0.0331 | 1.03 (0.94-1.1) | 0.49 | 0.486 |
| DDX3Y | 0.03 | 1.03 (0.99-1.1) | 2.52 | 0.113 |
| DDX3YP1 | 0.1 | 1.11 (0.97-1.3) | 2.15 | 0.143 |
| EIF1AY | 0.0449 | 1.05 (1-1.1) | 3.55 | 0.0595 |
| FAT1 | 0.122 | 1.13 (1.1-1.2) | 12.2 | 0.000482 |
| GLI2 | 0.00088 | 1 (0.93-1.1) | 0 | 0.982 |
| GPR126 | 0.0248 | 1.03 (0.96-1.1) | 0.48 | 0.487 |
| HMGA2 | 0.0902 | 1.09 (1-1.2) | 7.61 | 0.00579 |
| HOXB_AS3 | 0.0238 | 1.02 (0.97-1.1) | 0.69 | 0.405 |
| IRX5 | 0.00728 | 1.01 (0.94-1.1) | 0.04 | 0.847 |
| JPH1 | 0.0377 | 1.04 (0.95-1.1) | 0.77 | 0.38 |
| KALP | 0.0846 | 1.09 (1-1.2) | 3.73 | 0.0536 |
| KDM5D | 0.0332 | 1.03 (1-1.1) | 2.95 | 0.086 |
| LINC00278 | 0.0741 | 1.08 (0.99-1.2) | 2.71 | 0.0998 |
| MDGA1 | 0.00575 | 1.01 (0.93-1.1) | 0.02 | 0.891 |
| MKRN3 | -0.0107 | 0.989 (0.91-1.1) | 0.07 | 0.791 |
| NETO1 | 0.0788 | 1.08 (1-1.2) | 4.29 | 0.0383 |
| NLRP2 | 0.0739 | 1.08 (0.99-1.2) | 3.19 | 0.0742 |
| PRKY | 0.039 | 1.04 (0.98-1.1) | 1.81 | 0.179 |
| RASGRF1 | -0.0549 | 0.947 (0.83-1.1) | 0.66 | 0.416 |
| RP11_424G14_1 | 0.0547 | 1.06 (0.98-1.1) | 2.14 | 0.144 |
| RP11_576C2_1 | 0.0804 | 1.08 (0.95-1.2) | 1.33 | 0.249 |
| RP11_713C5_1 | 0.0791 | 1.08 (0.99-1.2) | 2.78 | 0.0956 |
| RPS4Y1 | 0.0298 | 1.03 (0.99-1.1) | 2.66 | 0.103 |
| SCRN1 | 0.0711 | 1.07 (0.99-1.2) | 2.81 | 0.0937 |
| SIGLEC6 | 0.0556 | 1.06 (0.94-1.2) | 0.86 | 0.354 |
| TMSB4Y | 0.0975 | 1.1 (1-1.2) | 4.52 | 0.0335 |
| TSIX | -0.0461 | 0.955 (0.86-1.1) | 0.7 | 0.402 |
| TTTY10 | 0.138 | 1.15 (1-1.3) | 5.11 | 0.0238 |
| TTTY14 | 0.0646 | 1.07 (0.99-1.2) | 2.81 | 0.0936 |
| TTTY15 | 0.0463 | 1.05 (0.99-1.1) | 3.07 | 0.08 |
| TXLNG2P | 0.0342 | 1.03 (0.99-1.1) | 2.84 | 0.092 |
| UGT3A2 | 0.0101 | 1.01 (0.94-1.1) | 0.08 | 0.778 |
| USP9Y | 0.0402 | 1.04 (0.99-1.1) | 3.01 | 0.0826 |
| UTY | 0.0377 | 1.04 (0.99-1.1) | 2.6 | 0.107 |
| XIST | -0.0202 | 0.98 (0.95-1) | 1.54 | 0.215 |
| ZFY | 0.0391 | 1.04 (0.99-1.1) | 2.49 | 0.114 |
|  |  |  |  |  |

 Supplementary figure 10: Forest plot showing results of Cox Proportional-Hazards model including the five genes where expression was identified as significantly correlated with outcome by univariate analysis


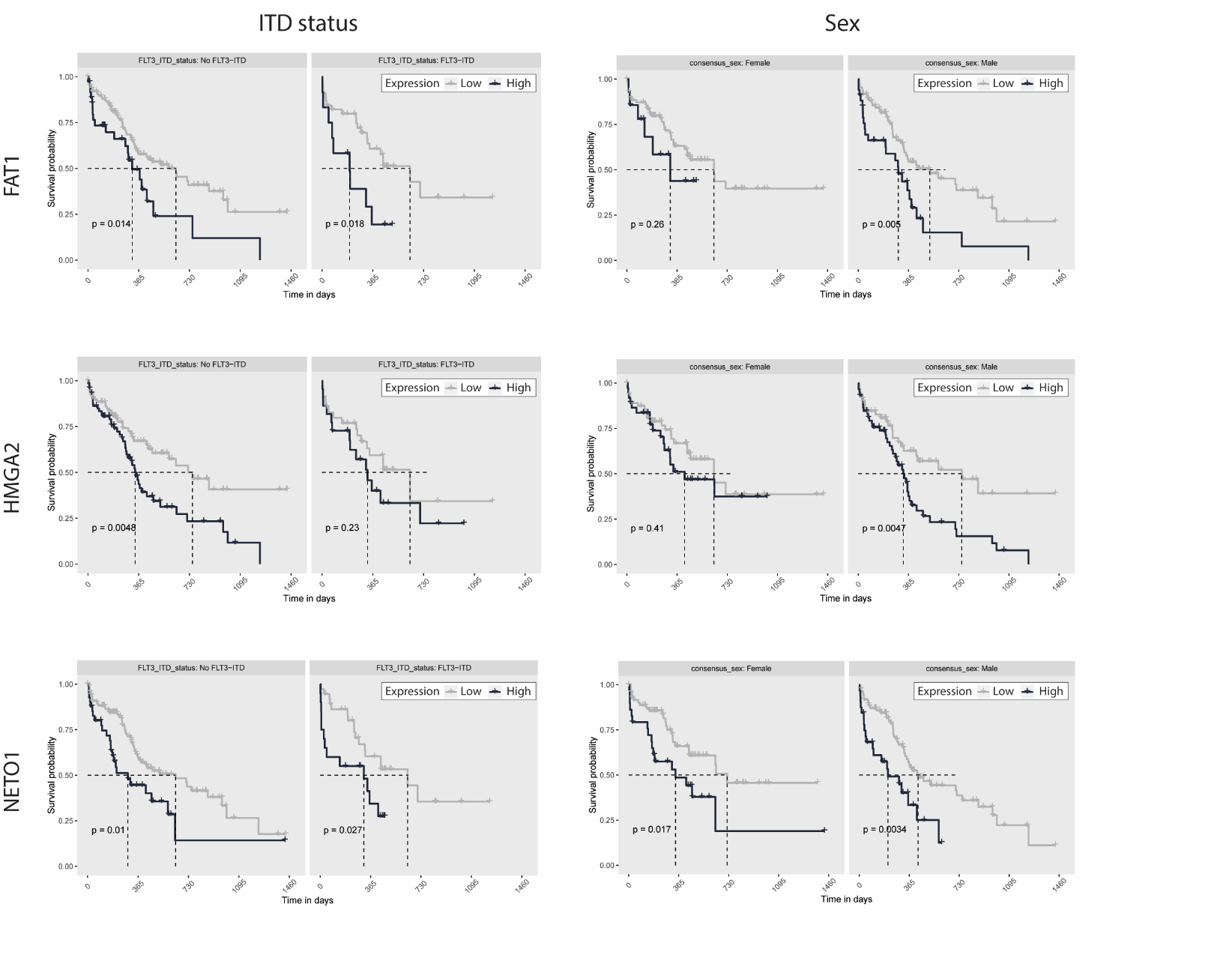


Supplementary figure 11: Kaplan-Meier curves comparing the outcome of patients with high vs low expression of NETO1 split by FLT3-ITD mutation status (left panels) and sex (right panels), respectively.

| Supplementary table 10 | | | | |
| --- | --- | --- | --- | --- |
| Number of samples in the drug screen | | | | |
| Inhibitor | Female - FLT3-ITD | Female - No FLT3-ITD | Male - FLT3-ITD | Male - No FLT3-ITD |
| 17-AAG (Tanespimycin) | 41 | 97 | 37 | 122 |
| A-674563 | 41 | 99 | 38 | 125 |
| ABT-737 | 29 | 71 | 31 | 95 |
| Afatinib (BIBW-2992) | 41 | 97 | 39 | 126 |
| Alisertib (MLN8237) | 40 | 97 | 40 | 126 |
| AT7519 | 39 | 95 | 34 | 124 |
| Axitinib (AG-013736) | 40 | 108 | 39 | 132 |
| AZD1480 | 40 | 96 | 38 | 123 |
| Barasertib (AZD1152-HQPA) | 41 | 94 | 40 | 123 |
| Bay 11-7085 | 29 | 69 | 26 | 84 |
| BEZ235 | 37 | 96 | 36 | 123 |
| BMS-345541 | 41 | 96 | 38 | 125 |
| Bortezomib (Velcade) | 42 | 98 | 42 | 125 |
| Bosutinib (SKI-606) | 40 | 98 | 38 | 124 |
| Cabozantinib | 41 | 94 | 37 | 123 |
| Canertinib (CI-1033) | 41 | 96 | 40 | 122 |
| Cediranib (AZD2171) | 40 | 97 | 38 | 122 |
| CHIR-99021 | 41 | 97 | 38 | 125 |
| CI-1040 (PD184352) | 41 | 97 | 39 | 124 |
| Crenolanib | 37 | 100 | 32 | 121 |
| Crizotinib (PF-2341066) | 44 | 109 | 39 | 137 |
| CYT387 | 41 | 96 | 39 | 126 |
| Dasatinib | 45 | 111 | 38 | 135 |
| DBZ | 32 | 72 | 29 | 94 |
| Doramapimod (BIRB 796) | 40 | 95 | 40 | 126 |
| Dovitinib (CHIR-258) | 40 | 97 | 38 | 125 |
| Elesclomol | 40 | 94 | 34 | 116 |
| Entospletinib (GS-9973) | 28 | 57 | 21 | 69 |
| Erlotinib | 43 | 103 | 40 | 137 |
| Flavopiridol | 40 | 97 | 39 | 125 |
| Foretinib (XL880) | 40 | 96 | 38 | 124 |
| GDC-0879 | 39 | 98 | 37 | 123 |
| GDC-0941 | 35 | 92 | 35 | 115 |
| Gefitinib | 41 | 109 | 40 | 133 |
| Gilteritinib (ASP-2215) | 23 | 43 | 14 | 54 |
| GSK-1838705A | 39 | 97 | 38 | 123 |
| GSK-1904529A | 38 | 96 | 37 | 126 |
| GSK690693 | 40 | 95 | 37 | 123 |
| GW-2580 | 37 | 96 | 35 | 119 |
| Ibrutinib (PCI-32765) | 39 | 95 | 34 | 115 |
| Idelalisib | 43 | 93 | 38 | 124 |
| Imatinib | 46 | 113 | 42 | 137 |
| INK-128 | 41 | 95 | 40 | 126 |
| JAK Inhibitor I | 39 | 97 | 40 | 125 |
| JNJ-28312141 | 41 | 93 | 35 | 122 |
| JNJ-38877605 | 40 | 97 | 38 | 124 |
| JNJ-7706621 | 41 | 97 | 40 | 127 |
| JQ1 | 31 | 70 | 26 | 84 |
| KI20227 | 38 | 98 | 37 | 123 |
| KU-55933 | 40 | 98 | 37 | 122 |
| KW-2449 | 39 | 94 | 38 | 125 |
| Lapatinib | 40 | 108 | 41 | 131 |
| Lenalidomide | 17 | 42 | 13 | 47 |
| Lenvatinib | 30 | 70 | 26 | 80 |
| Linifanib (ABT-869) | 41 | 97 | 38 | 124 |
| Lovastatin | 18 | 42 | 14 | 49 |
| LY-333531 | 41 | 98 | 40 | 126 |
| Masitinib (AB-1010) | 39 | 94 | 36 | 122 |
| MGCD-265 | 39 | 97 | 37 | 123 |
| Midostaurin | 40 | 90 | 39 | 126 |
| MK-2206 | 40 | 97 | 37 | 121 |
| MLN120B | 39 | 97 | 37 | 122 |
| MLN8054 | 41 | 97 | 38 | 123 |
| Motesanib (AMG-706) | 39 | 97 | 39 | 121 |
| Neratinib (HKI-272) | 40 | 97 | 38 | 124 |
| NF-kB Activation Inhibitor | 38 | 91 | 40 | 122 |
| Nilotinib | 46 | 110 | 39 | 131 |
| Nutlin 3a | 30 | 65 | 22 | 71 |
| NVP-ADW742 | 39 | 95 | 36 | 122 |
| NVP-TAE684 | 40 | 95 | 37 | 123 |
| Palbociclib | 22 | 52 | 19 | 59 |
| Panobinostat | 21 | 51 | 19 | 57 |
| Pazopanib (GW786034) | 37 | 110 | 41 | 129 |
| PD173955 | 40 | 99 | 36 | 126 |
| Pelitinib (EKB-569) | 40 | 96 | 39 | 122 |
| PHA-665752 | 39 | 98 | 38 | 124 |
| PHT-427 | 39 | 96 | 38 | 119 |
| PI-103 | 41 | 96 | 40 | 122 |
| Ponatinib (AP24534) | 44 | 100 | 41 | 121 |
| PP242 | 41 | 97 | 37 | 125 |
| PRT062607 | 40 | 96 | 37 | 124 |
| Quizartinib (AC220) | 40 | 97 | 38 | 125 |
| RAF265 (CHIR-265) | 40 | 97 | 38 | 120 |
| Rapamycin | 40 | 105 | 42 | 129 |
| Regorafenib (BAY 73-4506) | 41 | 96 | 37 | 122 |
| Roscovitine (CYC-202) | 40 | 98 | 40 | 127 |
| Ruxolitinib (INCB018424) | 46 | 109 | 35 | 127 |
| S31-201 | 41 | 95 | 37 | 121 |
| Saracatinib (AZD0530) | 40 | 96 | 37 | 122 |
| SB-431542 | 41 | 97 | 40 | 124 |
| Selumetinib (AZD6244) | 39 | 96 | 38 | 124 |
| SGX-523 | 40 | 95 | 38 | 122 |
| SNS-032 (BMS-387032) | 41 | 98 | 40 | 122 |
| Sorafenib | 46 | 110 | 42 | 136 |
| SR9011 | 18 | 42 | 14 | 48 |
| STO609 | 40 | 96 | 38 | 126 |
| SU11274 | 40 | 97 | 36 | 122 |
| Sunitinib | 44 | 110 | 43 | 133 |
| TG100-115 | 39 | 95 | 38 | 122 |
| Tivozanib (AV-951) | 40 | 96 | 38 | 124 |
| Tofacitinib (CP-690550) | 41 | 96 | 39 | 125 |
| Tozasertib (VX-680) | 40 | 98 | 39 | 126 |
| Trametinib (GSK1120212) | 45 | 103 | 39 | 129 |
| Vandetanib (ZD6474) | 40 | 106 | 40 | 136 |
| Vargetef | 39 | 97 | 38 | 121 |
| Vatalanib (PTK787) | 39 | 97 | 39 | 123 |
| Vemurafenib (PLX-4032) | 33 | 74 | 27 | 90 |
| Venetoclax | 35 | 72 | 26 | 84 |
| Vismodegib (GDC-0449) | 41 | 96 | 37 | 125 |
| Volasertib (BI-6727) | 32 | 68 | 24 | 85 |
| VX-745 | 41 | 96 | 40 | 125 |
| XAV-939 | 40 | 96 | 36 | 125 |
| YM-155 | 41 | 97 | 40 | 125 |
|  |  |  |  |  |

Supplementary figure 12: Overview of drugs in the drug screen that were assigned a class.


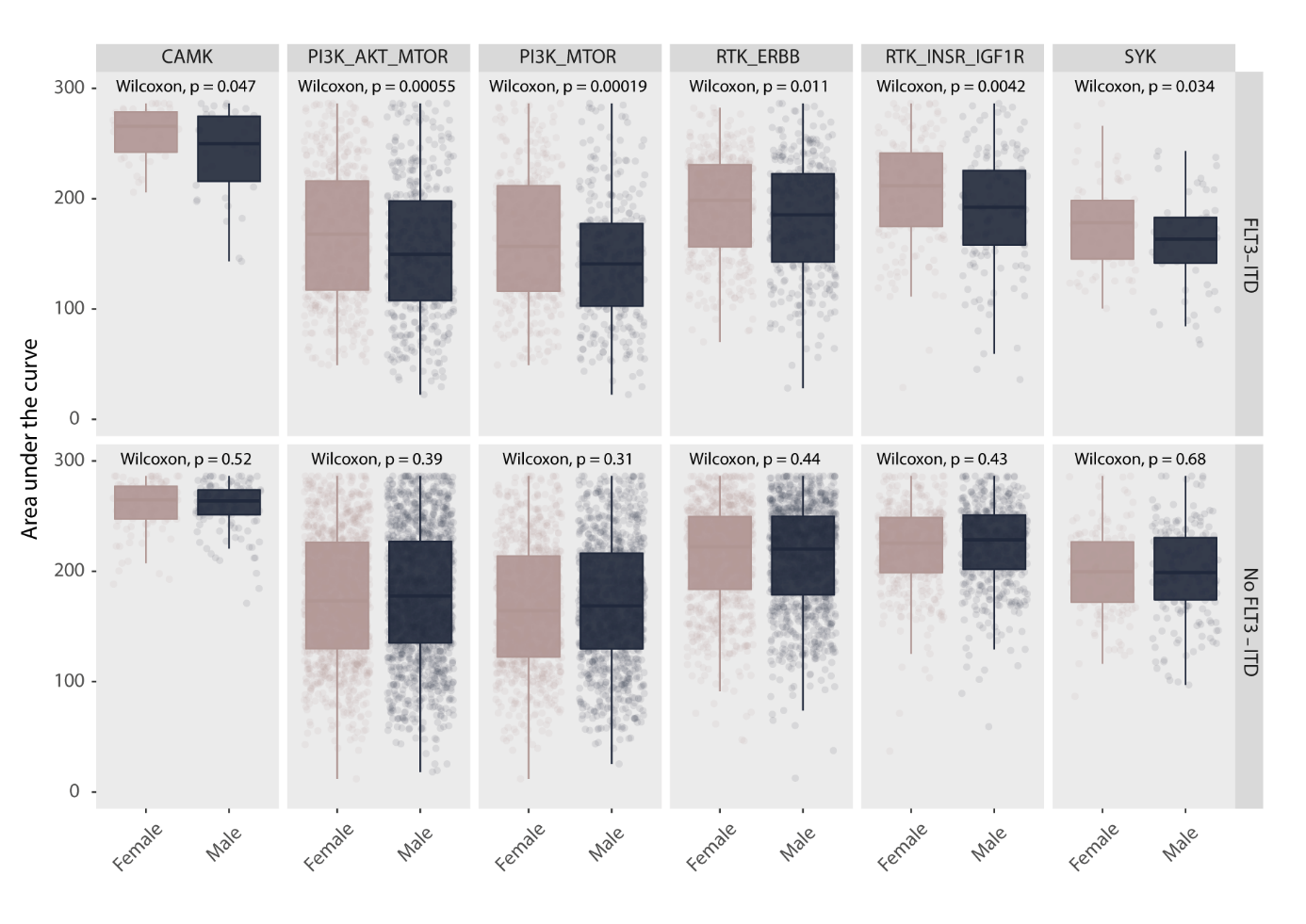


Supplementary figure 13: Comparison of area under the curve (AUC) between FLT3-ITD mutated female and male specimens for the various drug classes.

| Supplementary table 11  p-values of comparisons of drug sensitivity | | | | | | |
| --- | --- | --- | --- | --- | --- | --- |
| Inhibitor | FLT3-ITD vs no FLT3-ITD | Male: FLT3-ITD vs no FLT3-ITD | Female: FLT3-ITD vs no FLT3-ITD | Male vs female | FLT3-ITD: Male vs female | no FLT3-ITD: Male vs female |
|  |  |  |  |  |  |  |
| 17-AAG (Tanespimycin) | 0.000 | 0.000 | 0.000 | 0.149 | 0.564 | 0.234 |
| A-674563 | 0.000 | 0.000 | 0.000 | 0.375 | 0.819 | 0.604 |
| ABT-737 | 0.208 | 0.939 | 0.079 | 0.788 | 0.346 | 0.307 |
| Afatinib (BIBW-2992) | 0.007 | 0.004 | 0.250 | 0.113 | 0.035 | 0.402 |
| Alisertib (MLN8237) | 0.002 | 0.019 | 0.029 | 0.717 | 0.391 | 0.743 |
| AT7519 | 0.072 | 0.084 | 0.537 | 0.042 | 0.805 | 0.041 |
| Axitinib (AG-013736) | 0.000 | 0.000 | 0.100 | 0.560 | 0.049 | 0.783 |
| AZD1480 | 0.000 | 0.001 | 0.000 | 0.455 | 0.739 | 0.297 |
| Barasertib (AZD1152-HQPA) | 0.000 | 0.000 | 0.001 | 0.556 | 0.192 | 0.707 |
| Bay 11-7085 | 0.001 | 0.006 | 0.081 | 0.655 | 0.530 | 0.554 |
| BEZ235 | 0.110 | 0.012 | 0.742 | 0.359 | 0.012 | 0.846 |
| BMS-345541 | 0.342 | 0.097 | 0.805 | 0.304 | 0.116 | 0.769 |
| Bortezomib (Velcade) | 0.111 | 0.243 | 0.282 | 0.944 | 0.918 | 0.976 |
| Bosutinib (SKI-606) | 0.000 | 0.000 | 0.001 | 0.472 | 0.076 | 0.774 |
| Cabozantinib | 0.000 | 0.000 | 0.000 | 0.188 | 0.370 | 0.265 |
| Canertinib (CI-1033) | 0.001 | 0.002 | 0.066 | 0.210 | 0.070 | 0.438 |
| Cediranib (AZD2171) | 0.108 | 0.057 | 0.619 | 0.033 | 0.046 | 0.167 |
| CHIR-99021 | 0.606 | 0.312 | 0.959 | 0.057 | 0.121 | 0.166 |
| CI-1040 (PD184352) | 0.724 | 0.214 | 0.394 | 0.423 | 0.338 | 0.179 |
| Crenolanib | 0.000 | 0.000 | 0.000 | 0.120 | 0.862 | 0.220 |
| Crizotinib (PF-2341066) | 0.000 | 0.001 | 0.076 | 0.381 | 0.132 | 0.705 |
| CYT387 | 0.000 | 0.000 | 0.001 | 0.964 | 0.283 | 1.000 |
| Dasatinib | 0.005 | 0.014 | 0.115 | 0.809 | 0.699 | 0.719 |
| DBZ | 0.002 | 0.014 | 0.058 | 0.373 | 0.247 | 0.537 |
| Doramapimod (BIRB 796) | 0.340 | 0.324 | 0.695 | 0.974 | 0.749 | 0.905 |
| Dovitinib (CHIR-258) | 0.000 | 0.000 | 0.000 | 0.314 | 0.471 | 0.323 |
| Elesclomol | 0.011 | 0.211 | 0.037 | 0.820 | 0.542 | 0.410 |
| Entospletinib (GS-9973) | 0.000 | 0.002 | 0.003 | 0.610 | 0.452 | 0.513 |
| Erlotinib | 0.000 | 0.000 | 0.000 | 0.891 | 0.996 | 0.390 |
| Flavopiridol | 0.449 | 0.337 | 0.923 | 0.968 | 0.511 | 0.791 |
| Foretinib (XL880) | 0.000 | 0.000 | 0.000 | 0.067 | 0.941 | 0.096 |
| GDC-0879 | 0.937 | 0.472 | 0.555 | 0.367 | 0.125 | 0.839 |
| GDC-0941 | 0.042 | 0.016 | 0.920 | 0.640 | 0.350 | 0.285 |
| Gefitinib | 0.000 | 0.000 | 0.000 | 0.832 | 0.354 | 0.826 |
| Gilteritinib (ASP-2215) | 0.000 | 0.001 | 0.000 | 0.353 | 0.865 | 0.893 |
| GSK-1838705A | 0.105 | 0.049 | 0.765 | 0.496 | 0.185 | 0.966 |
| GSK-1904529A | 0.195 | 0.026 | 0.730 | 0.588 | 0.126 | 0.705 |
| GSK690693 | 0.010 | 0.054 | 0.107 | 0.359 | 0.939 | 0.353 |
| GW-2580 | 0.080 | 0.037 | 0.653 | 0.814 | 0.234 | 0.745 |
| Ibrutinib (PCI-32765) | 0.000 | 0.000 | 0.000 | 0.521 | 0.314 | 0.397 |
| Idelalisib | 0.057 | 0.003 | 0.996 | 0.077 | 0.009 | 0.537 |
| Imatinib | 0.006 | 0.076 | 0.027 | 0.406 | 0.871 | 0.253 |
| INK-128 | 0.000 | 0.000 | 0.009 | 0.519 | 0.290 | 0.407 |
| JAK Inhibitor I | 0.000 | 0.000 | 0.002 | 0.518 | 0.185 | 0.591 |
| JNJ-28312141 | 0.000 | 0.000 | 0.000 | 0.996 | 0.641 | 0.889 |
| JNJ-38877605 | 0.902 | 0.995 | 0.753 | 0.578 | 0.996 | 0.545 |
| JNJ-7706621 | 0.004 | 0.049 | 0.060 | 0.114 | 0.892 | 0.139 |
| JQ1 | 0.647 | 0.160 | 0.429 | 0.406 | 0.304 | 0.112 |
| KI20227 | 0.002 | 0.001 | 0.172 | 0.267 | 0.091 | 0.638 |
| KU-55933 | 0.663 | 0.512 | 0.987 | 0.280 | 0.328 | 0.435 |
| KW-2449 | 0.000 | 0.000 | 0.000 | 0.152 | 0.426 | 0.387 |
| Lapatinib | 0.001 | 0.000 | 0.472 | 0.621 | 0.046 | 0.594 |
| Lenalidomide | 0.008 | 0.092 | 0.039 | 0.981 | 0.967 | 0.752 |
| Lenvatinib | 0.000 | 0.000 | 0.002 | 0.333 | 0.726 | 0.356 |
| Linifanib (ABT-869) | 0.000 | 0.002 | 0.000 | 0.051 | 0.144 | 0.382 |
| Lovastatin | 0.651 | 0.800 | 0.434 | 0.669 | 0.442 | 0.978 |
| LY-333531 | 0.000 | 0.000 | 0.000 | 0.661 | 0.344 | 0.583 |
| Masitinib (AB-1010) | 0.003 | 0.000 | 0.561 | 0.404 | 0.019 | 0.872 |
| MGCD-265 | 0.000 | 0.000 | 0.000 | 0.246 | 0.013 | 0.586 |
| Midostaurin | 0.000 | 0.000 | 0.000 | 0.650 | 0.872 | 0.136 |
| MK-2206 | 0.397 | 0.539 | 0.538 | 0.522 | 0.870 | 0.512 |
| MLN120B | 0.013 | 0.014 | 0.250 | 0.814 | 0.480 | 0.942 |
| MLN8054 | 0.286 | 0.408 | 0.459 | 0.753 | 0.766 | 0.767 |
| Motesanib (AMG-706) | 0.002 | 0.042 | 0.015 | 0.844 | 0.796 | 0.974 |
| Neratinib (HKI-272) | 0.000 | 0.000 | 0.087 | 0.528 | 0.342 | 0.386 |
| NF-kB Activation Inhibitor | 0.000 | 0.000 | 0.007 | 0.355 | 0.901 | 0.337 |
| Nilotinib | 0.963 | 0.700 | 0.662 | 0.589 | 0.860 | 0.431 |
| Nutlin 3a | 0.002 | 0.088 | 0.019 | 0.093 | 0.147 | 0.303 |
| NVP-ADW742 | 0.000 | 0.000 | 0.041 | 0.335 | 0.022 | 0.977 |
| NVP-TAE684 | 0.000 | 0.000 | 0.000 | 0.453 | 0.202 | 0.344 |
| Palbociclib | 0.000 | 0.004 | 0.068 | 0.013 | 0.969 | 0.007 |
| Panobinostat | 0.177 | 0.900 | 0.069 | 0.633 | 0.616 | 0.276 |
| Pazopanib (GW786034) | 0.000 | 0.000 | 0.000 | 0.048 | 0.068 | 0.106 |
| PD173955 | 0.000 | 0.001 | 0.003 | 0.542 | 0.531 | 0.434 |
| Pelitinib (EKB-569) | 0.000 | 0.000 | 0.002 | 0.682 | 0.160 | 0.923 |
| PHA-665752 | 0.000 | 0.000 | 0.000 | 0.594 | 0.719 | 0.684 |
| PHT-427 | 0.733 | 0.345 | 0.652 | 0.535 | 0.663 | 0.316 |
| PI-103 | 0.012 | 0.001 | 0.801 | 0.693 | 0.040 | 0.447 |
| Ponatinib (AP24534) | 0.000 | 0.000 | 0.000 | 0.621 | 0.609 | 0.893 |
| PP242 | 0.000 | 0.000 | 0.048 | 0.626 | 0.148 | 0.938 |
| PRT062607 | 0.000 | 0.000 | 0.009 | 0.720 | 0.037 | 0.338 |
| Quizartinib (AC220) | 0.000 | 0.000 | 0.000 | 0.031 | 0.831 | 0.099 |
| RAF265 (CHIR-265) | 0.002 | 0.018 | 0.040 | 0.576 | 0.956 | 0.584 |
| Rapamycin | 0.090 | 0.016 | 0.981 | 0.918 | 0.094 | 0.459 |
| Regorafenib (BAY 73-4506) | 0.000 | 0.000 | 0.000 | 0.906 | 0.386 | 0.828 |
| Roscovitine (CYC-202) | 0.000 | 0.000 | 0.034 | 0.449 | 0.070 | 0.983 |
| Ruxolitinib (INCB018424) | 0.112 | 0.084 | 0.545 | 0.818 | 0.360 | 0.928 |
| S31-201 | 0.970 | 0.102 | 0.119 | 0.601 | 0.032 | 0.447 |
| Saracatinib (AZD0530) | 0.001 | 0.004 | 0.063 | 0.253 | 0.111 | 0.498 |
| SB-431542 | 0.858 | 0.382 | 0.628 | 0.316 | 0.176 | 0.756 |
| Selumetinib (AZD6244) | 0.373 | 0.802 | 0.111 | 0.574 | 0.412 | 0.220 |
| SGX-523 | 0.035 | 0.011 | 0.679 | 0.833 | 0.108 | 0.569 |
| SNS-032 (BMS-387032) | 0.142 | 0.261 | 0.436 | 0.070 | 0.526 | 0.105 |
| Sorafenib | 0.000 | 0.000 | 0.000 | 0.324 | 0.288 | 0.507 |
| SR9011 | 0.124 | 0.724 | 0.027 | 0.191 | 0.054 | 0.856 |
| STO609 | 0.178 | 0.027 | 0.977 | 0.132 | 0.047 | 0.519 |
| SU11274 | 0.003 | 0.072 | 0.017 | 0.185 | 0.485 | 0.397 |
| Sunitinib | 0.000 | 0.000 | 0.000 | 0.149 | 0.439 | 0.194 |
| TG100-115 | 0.323 | 0.086 | 0.628 | 0.617 | 0.235 | 0.151 |
| Tivozanib (AV-951) | 0.000 | 0.000 | 0.002 | 0.130 | 0.028 | 0.280 |
| Tofacitinib (CP-690550) | 0.168 | 0.340 | 0.399 | 0.125 | 0.484 | 0.205 |
| Tozasertib (VX-680) | 0.000 | 0.000 | 0.000 | 0.140 | 0.996 | 0.403 |
| Trametinib (GSK1120212) | 0.372 | 0.030 | 0.315 | 0.183 | 0.252 | 0.046 |
| Vandetanib (ZD6474) | 0.000 | 0.000 | 0.000 | 0.936 | 0.741 | 0.754 |
| Vargetef | 0.000 | 0.000 | 0.000 | 0.515 | 0.284 | 0.655 |
| Vatalanib (PTK787) | 0.001 | 0.048 | 0.004 | 0.573 | 0.758 | 0.301 |
| Vemurafenib (PLX-4032) | 0.000 | 0.000 | 0.007 | 0.422 | 0.375 | 0.450 |
| Venetoclax | 0.009 | 0.032 | 0.174 | 0.057 | 0.925 | 0.057 |
| Vismodegib (GDC-0449) | 0.139 | 0.196 | 0.351 | 0.374 | 0.410 | 0.400 |
| Volasertib (BI-6727) | 0.201 | 0.155 | 0.816 | 0.283 | 0.786 | 0.176 |
| VX-745 | 0.002 | 0.028 | 0.027 | 0.709 | 1.000 | 0.947 |
| XAV-939 | 0.070 | 0.484 | 0.082 | 0.452 | 0.328 | 0.940 |
| YM-155 | 0.992 | 0.542 | 0.459 | 0.515 | 0.681 | 0.287 |
|  |  |  |  |  |  |  |

Supplementary figure 14: Extension of figure 3A in the paper including the comparison between female and male not-FLT3-ITD positive samples.


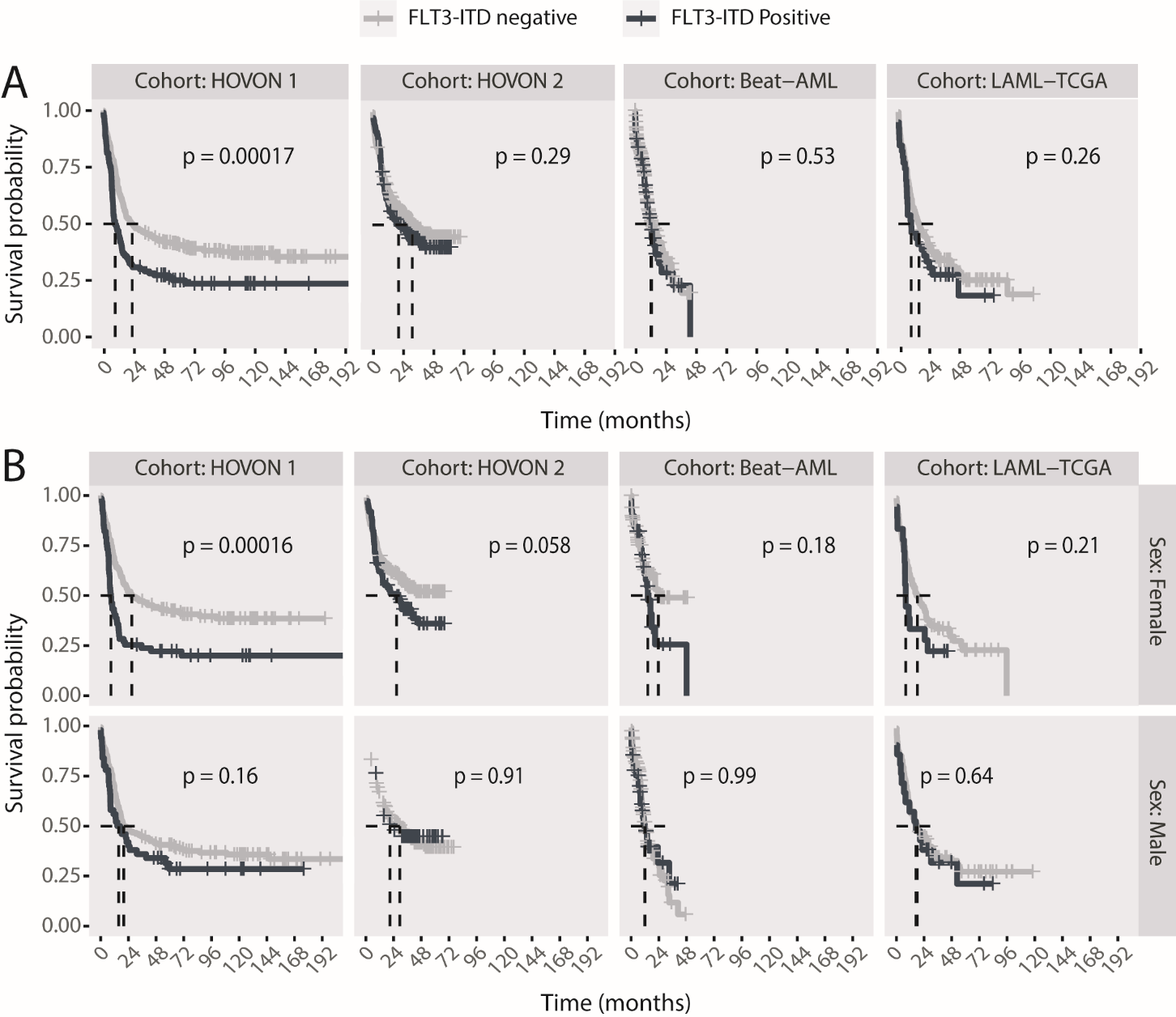


Supplementary figure 15: A. Kaplan-Meier curve comparing the outcome of FLT3-ITD mutated and FLT3 wt patients in each of the respective cohorts. B. Kaplan-Meier curve comparing the outcome of FLT3-ITD mutated and FLT3 wt patients in the four individual cohorts separated by sex. Number of individuals in each arm is summarized in Supplementary tables 12 b-e. The 2-sided log-rank test was applied to compare the Kaplan-Meier estimates. P-values indicated in each individual plot.

Supplementary figure 16: Kaplan-Meier curve comparing the outcome of female and male patients across the four cohorts separated by FLT3-ITD mutation status. Number of individuals in each arm is summarized in Supplementary tables 12 b-e. The 2-sided log-rank test was applied to compare the Kaplan-Meier estimates. P-values indicated in each individual plot.

| Supplementary table 12 a  Survival analysis of all cohorts combined | | | | | | | | | | | | | | |
| --- | --- | --- | --- | --- | --- | --- | --- | --- | --- | --- | --- | --- | --- | --- |
|  |  |  |  |  |  |  |  |  |  |  |  |  |  |  |
|  | FLT3-ITD negative female | | | | | |  | FLT3-ITD positive female | | | | | |  |
|  |  |  |  |  |  |  |  |  |  |  |  |  |  |  |
|  | Time (months) | Number at risk | Number of event | Survival | standard error | 95% CI |  | Time (months) | Number at risk | Number of event | Survival | standard error | 95% CI |  |
|  | Median | 523 | 268 | 32.1 months |  | (24.8-46.8) |  | Median | 198 | 133 | 13.9 months |  | (10.2-16.3) |  |
|  | 12 | 316 | 168 | 0.665 | 0.0212 | (0.625-0.708) |  | 12 | 97 | 94 | 0.517 | 0.0359 | (0.451-0.592) |  |
|  | 24 | 234 | 54 | 0.546 | 0.0228 | (0.503-0.592) |  | 24 | 62 | 25 | 0.375 | 0.0356 | (0.312-0.452) |  |
|  | 36 | 173 | 26 | 0.481 | 0.0234 | (0.437-0.529) |  | 36 | 41 | 8 | 0.323 | 0.0352 | (0.261-0.4) |  |
|  | 48 | 111 | 10 | 0.447 | 0.0241 | (0.402-0.497) |  | 48 | 27 | 4 | 0.287 | 0.0357 | (0.224-0.366) |  |
|  | 60 | 79 | 5 | 0.424 | 0.025 | (0.378-0.476) |  | 60 | 20 | 1 | 0.276 | 0.0359 | (0.214-0.356) |  |
|  |  |  |  |  |  |  |  |  |  |  |  |  |  |  |
|  |  |  |  |  |  |  |  |  |  |  |  |  |  |  |
|  |  |  |  |  |  |  |  |  |  |  |  |  |  |  |
|  | FLT3-ITD negative male | | | | | |  | FLT3-ITD positive male | | | | | |  |
|  |  |  |  |  |  |  |  |  |  |  |  |  |  |  |
|  | Time (months) | Number at risk | Number of event | Survival | standard error | 95% CI |  | Time (months) | Number at risk | Number of event | Survival | standard error | 95% CI |  |
|  | Median | 655 | 390 | 19.5 months |  | (17.3-27) |  | Median | 184 | 112 | 16.9 months |  | (11.9-24.1) |  |
|  | 12 | 384 | 235 | 0.63 | 0.0192 | (0.593-0.669) |  | 12 | 97 | 78 | 0.565 | 0.0372 | (0.496-0.642) |  |
|  | 24 | 267 | 91 | 0.475 | 0.0202 | (0.437-0.517) |  | 24 | 65 | 23 | 0.421 | 0.038 | (0.353-0.503) |  |
|  | 36 | 186 | 37 | 0.404 | 0.0203 | (0.366-0.446) |  | 36 | 49 | 7 | 0.373 | 0.0378 | (0.306-0.455) |  |
|  | 48 | 127 | 14 | 0.369 | 0.0206 | (0.331-0.412) |  | 48 | 28 | 1 | 0.365 | 0.0379 | (0.297-0.447) |  |
|  | 60 | 86 | 6 | 0.35 | 0.021 | (0.311-0.394) |  | 60 | 15 | 3 | 0.313 | 0.0428 | (0.24-0.409) |  |
|  |  |  |  |  |  |  |  |  |  |  |  |  |  |  |
|  |  |  |  |  |  |  |  |  |  |  |  |  |  |  |

| Supplementary table 12 b  Survival analysis of the Beat AML cohort | | | | | | | | | | | | | | |
| --- | --- | --- | --- | --- | --- | --- | --- | --- | --- | --- | --- | --- | --- | --- |
|  |  |  |  |  |  |  |  |  |  |  |  |  |  |  |
|  | FLT3-ITD negative female | | | | | |  | FLT3-ITD positive female | | | | | |  |
|  |  |  |  |  |  |  |  |  |  |  |  |  |  |  |
|  | Time (months) | Number at risk | Number of event | Survival | standard error | 95% CI |  | Time (months) | Number at risk | Number of event | Survival | standard error | 95% CI |  |
|  | Median | 102 | 34 | 23.6 months |  | (20.8-NA) |  | Median | 39 | 22 | 14.4 months |  | (11.24-NA) |  |
|  | 12 | 32 | 30 | 0.629 | 0.0571 | (0.526-0.751) |  | 12 | 19 | 14 | 0.61 | 0.0826 | (0.468-0.795) |  |
|  | 24 | 12 | 4 | 0.49 | 0.0772 | (0.36-0.668) |  | 24 | 3 | 7 | 0.256 | 0.1067 | (0.113-0.579) |  |
|  | 36 | 4 | 0 | 0.49 | 0.0772 | (0.36-0.668) |  | 36 | 1 | 0 | 0.256 | 0.1067 | (0.113-0.579) |  |
|  | 48 | 1 | 0 | 0.49 | 0.0772 | (0.36-0.668) |  | 48 | 1 | 0 | 0.256 | 0.1067 | (0.113-0.579) |  |
|  | 60 |  |  |  |  |  |  | 60 |  |  |  |  |  |  |
|  |  |  |  |  |  |  |  |  |  |  |  |  |  |  |
|  |  |  |  |  |  |  |  |  |  |  |  |  |  |  |
|  |  |  |  |  |  |  |  |  |  |  |  |  |  |  |
|  | FLT3-ITD negative male | | | | | |  | FLT3-ITD positive male | | | | | |  |
|  |  |  |  |  |  |  |  |  |  |  |  |  |  |  |
|  | Time (months) | Number at risk | Number of event | Survival | standard error | 95% CI |  | Time (months) | Number at risk | Number of event | Survival months | standard error | 95% CI |  |
|  | Median | 121 | 69 | 12 months |  | (10.15-17.1) |  | Median | 41 | 23 | 11.7 months |  | (8.12-NA) |  |
|  | 12 | 38 | 50 | 0.494 | 0.0523 | (0.4014-0.608) |  | 12 | 14 | 19 | 0.475 | 0.086 | (0.333-0.677) |  |
|  | 24 | 15 | 11 | 0.317 | 0.0554 | (0.2252-0.447) |  | 24 | 4 | 3 | 0.317 | 0.0999 | (0.1706-0.587) |  |
|  | 36 | 2 | 7 | 0.117 | 0.0545 | (0.0473-0.292) |  | 36 | 2 | 1 | 0.211 | 0.1089 | (0.0768-0.58) |  |
|  | 48 |  |  |  |  |  |  | 48 |  |  |  |  |  |  |
|  | 60 |  |  |  |  |  |  | 60 |  |  |  |  |  |  |
|  |  |  |  |  |  |  |  |  |  |  |  |  |  |  |
|  |  |  |  |  |  |  |  |  |  |  |  |  |  |  |

|  | Supplementary table 12 c  Survival analysis of the LAML-TCGA cohort | | | | | | | | | | | | |  |
| --- | --- | --- | --- | --- | --- | --- | --- | --- | --- | --- | --- | --- | --- | --- |
|  |  |  |  |  |  |  |  |  |  |  |  |  |  |  |
|  | FLT3-ITD negative female | | | | | |  | FLT3-ITD positive female | | | | | |  |
|  |  |  |  |  |  |  |  |  |  |  |  |  |  |  |
|  | Time (months) | Number at risk | Number of event | Survival | standard error | 95% CI |  | Time (months) | Number at risk | Number of event | Survival | standard error | 95% CI |  |
|  | Median | 74 | 55 | 18.3 months |  | (11.8-28.4) |  | Median | 18 | 14 | 8.1 months |  | (7.1-NA) |  |
|  | 12 | 44 | 30 | 0.595 0 | 0.0571 | (0.493-0.718) |  | 12 | 6 | 12 | 0.333 | 0.111 | (0.1734-0.641) |  |
|  | 24 | 33 | 11 | 0.446 | 0.0578 | (0.346-0.575) |  | 24 | 6 | 0 | 0.333 | 0.111 | (0.1734-0.641) |  |
|  | 36 | 22 | 8 | 0.335 | 0.0552 | (0.243-0.463) |  | 36 | 3 | 2 | 0.222 | 0.098 | (0.0936-0.527) |  |
|  | 48 | 14 | 2 | 0.296 | 0.0553 | (0.205-0.427) |  | 48 |  |  |  |  |  |  |
|  | 60 | 7 | 3 | 0.227 | 0.0548 | (0.142-0.365) |  | 60 |  |  |  |  |  |  |
|  |  |  |  |  |  |  |  |  |  |  |  |  |  |  |
|  |  |  |  |  |  |  |  |  |  |  |  |  |  |  |
|  |  |  |  |  |  |  |  |  |  |  |  |  |  |  |
|  | FLT3-ITD negative male | | | | | |  | FLT3-ITD positive male | | | | | |  |
|  |  |  |  |  |  |  |  |  |  |  |  |  |  |  |
|  | Time (months) | Number at risk | Number of event | Survival | standard error | 95% CI |  | Time (months) | Number at risk | Number of event | Survival | standard error | 95% CI |  |
|  | Median | 87 | 60 | 18.1 months |  | (11.9-32.3) |  | Median | 21 | 15 | 17 months |  | (6.6-NA) |  |
|  | 12 | 51 | 37 | 0.575 | 0.053 | (0.48-0.689) |  | 12 | 12 | 9 | 0.571 | 0.108 | (0.3945-0.828) |  |
|  | 24 | 38 | 11 | 0.448 | 0.0534 | (0.354-0.566) |  | 24 | 8 | 4 | 0.381 | 0.106 | (0.2208-0.657) |  |
|  | 36 | 24 | 8 | 0.344 | 0.0522 | (0.255-0.463) |  | 36 | 5 | 1 | 0.317 | 0.106 | (0.1654-0.609) |  |
|  | 48 | 18 | 1 | 0.327 | 0.0523 | (0.239-0.447) |  | 48 | 3 | 0 | 0.317 | 0.106 | (0.1654-0.609) |  |
|  | 60 | 14 | 3 | 0.272 | 0.0522 | (0.187-0.396) |  | 60 | 2 | 1 | 0.212 | 0.111 | (0.0754-0.594) |  |
|  |  |  |  |  |  |  |  |  |  |  |  |  |  |  |
|  |  |  |  |  |  |  |  |  |  |  |  |  |  |  |

| Supplementary table 12 d  Survival analysis of the HOVON1 cohort | | | | | | | | | | | | | |  |
| --- | --- | --- | --- | --- | --- | --- | --- | --- | --- | --- | --- | --- | --- | --- |
|  |  |  |  |  |  |  |  |  |  |  |  |  |  |  |
|  | FLT3-ITD negative female | | | | | |  | FLT3-ITD positive female | | | | | |  |
|  |  |  |  |  |  |  |  |  |  |  |  |  |  |  |
|  | Time (months) | Number at risk | Number of event | Survival | standard error | 95% CI |  | Time (months) | Number at risk | Number of event | Survival | standard error | 95% CI |  |
|  | Median | 148 | 89 | 27 months |  | (19.19-69.6) |  | Median | 67 | 53 | 8.9 months |  | (7.62-14.2) |  |
|  | 12 | 100 | 48 | 0.676 | 0.0385 | (0.604-0.755) |  | 12 | 27 | 40 | 0.403 0 | 0.0599 | (0.301-0.539) |  |
|  | 24 | 77 | 23 | 0.52 | 0.0411 | (0.446-0.607) |  | 24 | 17 | 10 | 0.254 | 0.0532 | (0.168-0.383) |  |
|  | 36 | 69 | 7 | 0.473 | 0.041 | (0.399-0.561) |  | 36 | 15 | 1 | 0.238 | 0.0522 | (0.155-0.366) |  |
|  | 48 | 63 | 5 | 0.438 | 0.0408 | (0.365-0.526) |  | 48 | 14 | 1 | 0.222 | 0.051 | (0.141-0.348) |  |
|  | 60 | 57 | 2 | 0.424 | 0.0408 | (0.351-0.512) |  | 60 | 12 | 0 | 0.222 | 0.051 | (0.141-0.348) |  |
|  |  |  |  |  |  |  |  |  |  |  |  |  |  |  |
|  |  |  |  |  |  |  |  |  |  |  |  |  |  |  |
|  | FLT3-ITD negative male | | | | | |  | FLT3-ITD positive male | | | | | |  |
|  |  |  |  |  |  |  |  |  |  |  |  |  |  |  |
|  | Time (months) | Number at risk | Number of event | Survival | standard error | 95% CI |  | Time (months) | Number at risk | Number of event | Survival | standard error | 95% CI |  |
|  | Median | 167 | 106 | 20.1 months |  | (16.59-47.3) |  | Median | 50 | 35 | 15.6 months |  | (8.38-56.1) |  |
|  | 12 | 113 | 54 | 0.677 | 0.0362 | (0.609-0.751) |  | 12 | 28 | 22 | 0.56 | 0.0702 | (0.438-0.716) |  |
|  | 24 | 82 | 31 | 0.491 | 0.0387 | (0.421-0.573) |  | 24 | 21 | 7 | 0.42 | 0.0698 | (0.303-0.582) |  |
|  | 36 | 74 | 7 | 0.449 | 0.0385 | (0.38-0.531) |  | 36 | 18 | 3 | 0.36 | 0.0679 | (0.249-0.521) |  |
|  | 48 | 66 | 6 | 0.412 | 0.0382 | (0.344-0.494) |  | 48 | 16 | 1 | 0.34 | 0.067 | (0.231-0.5) |  |
|  | 60 | 59 | 1 | 0.406 | 0.0381 | (0.338-0.488) |  | 60 | 9 | 2 | 0.285 | 0.0665 | (0.181-0.451) |  |
|  |  |  |  |  |  |  |  |  |  |  |  |  |  |  |
|  |  |  |  |  |  |  |  |  |  |  |  |  |  |  |

| Supplementary table 12 e  Survival analysis of the HOVON2 cohort | | | | | | | | | | | | | | |  |
| --- | --- | --- | --- | --- | --- | --- | --- | --- | --- | --- | --- | --- | --- | --- | --- |
|  |  | |  |  |  |  |  |  |  |  |  |  |  | |  |
| FLT3-ITD negative female | | | | | | |  | FLT3-ITD positive female | | | | | | |  |
|  |  | |  |  |  |  |  |  |  |  |  |  |  | |  |
| Time (months) | Number at risk | | Number of event | Survival | standard error | 95% CI |  | Time (months) | Number at risk | Number of event | Survival | standard error | 95% CI | |  |
| Median | 199 | | 90 | NA months |  | (33.2-NA) |  | Median | 74 | 44 | 26.6 months |  | (14.4-NA) | |  |
| 12 | 140 | | 60 | 0.698 | 0.0325 | (0.638-0.765) |  | 12 | 45 | 28 | 0.621 | 0.0565 | (0.519-0.742) | |  |
| 24 | 112 | | 16 | 0.616 | 0.0346 | (0.552-0.688) |  | 24 | 36 | 8 | 0.509 | 0.0585 | (0.407-0.638) | |  |
| 36 | 78 | | 11 | 0.548 | 0.0364 | (0.482-0.625) |  | 36 | 22 | 5 | 0.432 | 0.0591 | (0.33-0.564) | |  |
| 48 | 33 | | 3 | 0.52 | 0.0381 | (0.45-0.6) |  | 48 | 12 | 3 | 0.36 | 0.0621 | (0.257-0.505) | |  |
| 60 | 15 | | 0 | 0.52 | 0.0381 | (0.45-0.6) |  | 60 | 8 | 0 | 0.36 | 0.0621 | (0.257-0.505) | |  |
|  |  | |  |  |  |  |  |  |  |  |  |  |  | |  |
|  |  | |  |  |  |  |  |  |  |  |  |  |  | |  |
|  |  | |  |  |  |  |  |  |  |  |  |  |  | |  |
| FLT3-ITD negative male | | | | | | |  | FLT3-ITD positive male | | | | | | |  |
|  |  | |  |  |  |  |  |  |  |  |  |  |  | |  |
| Time (months) | Number at risk | | Number of event | Survival | standard error | 95% CI |  | Time (months) | Number at risk | Number of event | Survival | standard error | 95% CI | |  |
| Median | 280 | | 155 | 29.4 months |  | (18.7-41.6) |  | Median | 72 | 39 | 20.8 months |  | (13-NA) | |  |
| 12 | 182 | | 94 | 0.663 | 0.0283 | (0.61-0.721) |  | 12 | 43 | 28 | 0.608 | 0.0578 | (0.505-0.733) | |  |
| 24 | 132 | | 38 | 0.52 | 0.0303 | (0.464-0.583) |  | 24 | 32 | 9 | 0.478 | 0.0596 | (0.375-0.61) | |  |
| 36 | 86 | | 15 | 0.453 | 0.031 | (0.397-0.518) |  | 36 | 24 | 2 | 0.447 | 0.0596 | (0.345-0.581) | |  |
| 48 | 43 | | 6 | 0.415 | 0.0321 | (0.357-0.483) |  | 48 | 9 | 0 | 0.447 | 0.0596 | (0.345-0.581) | |  |
| 60 | 13 | | 2 | 0.395 | 0.0336 | (0.334-0.467) |  | 60 | 4 | 0 | 0.447 | 0.0596 | (0.345-0.581) | |  |
|  |  | |  |  |  |  |  |  |  |  |  |  |  | |  |
|  |  | |  |  |  |  |  |  |  |  |  |  |  | |  |
|  | | |  |  |  |  |  |  |  |  |  |  |  | |  |
|  | |  | | | | | | | | | | | |  |  |
|  | |  | | | | | | | | | | | |  |  |
|  | |  | | | | | | | | | | | |  |  |
